# Phenylseleno trifluoromethoxylation of alkenes

**DOI:** 10.3762/bjoc.20.207

**Published:** 2024-09-26

**Authors:** Clément Delobel, Armen Panossian, Gilles Hanquet, Frédéric R Leroux, Fabien Toulgoat, Thierry Billard

**Affiliations:** 1 Institute of Chemistry and Biochemistry (ICBMS - UMR 5246), CNRS, University Claude Bernard-Lyon 1, CPE Lyon, Lyon, France; 2 Université de Strasbourg, Université de Haute-Alsace, CNRS, UMR 7042-LIMA, ECPM, Strasbourg, France; 3 CPE Lyon, Lyon, France

**Keywords:** DNTFB, electrophilic addition, fluorine, selenium, trifluoromethoxy

## Abstract

Trifluoromethoxylated molecules and selenylated compounds find a wide range of interesting applications, but separately. In order to combine the potential of these two motifs and to propose a new class of compounds, we have developed an electrophilic phenylseleno trifluoromethoxylation of alkenes, which leads to β-selenylated trifluoromethoxylated compounds or, upon subsequent reduction, to the trifluoromethoxylated ones.

## Introduction

**Figure 1:**
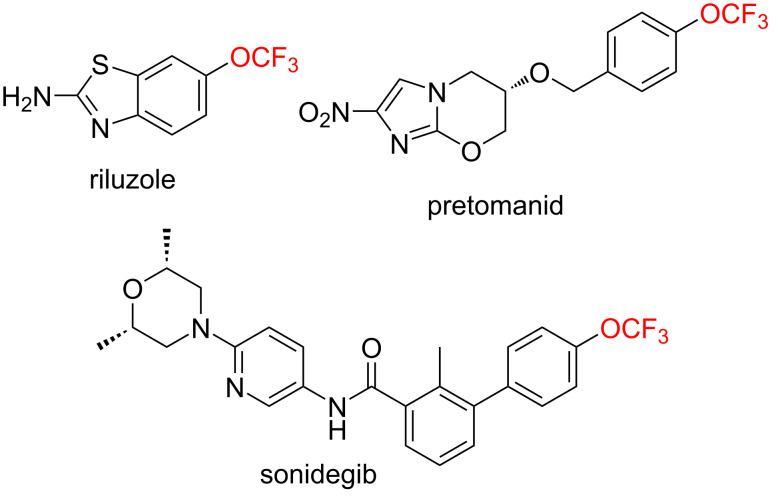
Examples of trifluoromethoxylated drugs.

Due to the specific properties of the fluorine atom [1–3], fluorinated compounds are now present in a wide range of applications, from materials to life sciences [4–11]. In order to propose new molecules with specific properties for targeted applications, the development of new fluorinated moieties is an active research field [12]. Among these emerging fluorinated groups, the association of the CF_3_ moiety with chalcogens is an interesting approach. In particular, the trifluoromethoxy group (CF_3_O) possesses valuable properties such as electronegativity [13–14], lipophilicity [15–16], electronic effects [17–18], and conformation [19–21]. Some trifluoromethoxylated molecules can be used as drugs in the treatment of various pathologies (Figure 1) [22–29].

On the other hand, despite its toxicity at higher doses, selenium is also an essential trace element in human physiology [30–31]. Moreover, several selenylated molecules have found applications in various fields such as materials or bioactive compounds [32–43]. Some selenylated compounds exhibit fascinating biological properties.

Despite these separate converging interests, no methods have been described to synthesize both trifluoromethoxylated and phenylselenylated molecules.

The introduction of a CF_3_O moiety into organic molecules remains poorly described in the literature, especially the direct trifluoromethoxylation [44–46]. Only few radical trifluoromethoxylations of (hetero)aromatics [47–52], enol carbonates [53] or silyl enol ethers and allyl silanes [54] have been reported. Most of the methods described have used the trifluoromethoxide anion (CF_3_O^−^) [45]. Many sources of the CF_3_O^−^ anion have been described, but with certain drawbacks such as their volatility, their tedious and expensive synthesis and the use of toxic reagents [55–65]. Recently, we reported the preparation of a stable solution of the CF_3_O^−^ anion (**DDPyOCF_3_**) from the cheap and commercially available 2,4-dinitro(trifluoromethoxy)benzene (**DNTFB**) [66–67]. This **DDPyOCF_3_** solution has shown a good reactivity to obtain various fluorinated compounds and especially trifluoromethoxylated molecules [68–71].

As another chapter of this research program, we propose here an easy and complementary access to CF_3_O-substituted alkyl compounds from alkenes and **DDPyOCF_3_**, more precisely to α-trifluoromethoxylated, β-phenylselenylated compounds.

## Results and Discussion

The electrophilic addition of phenylselenyl halides to alkenes to form a selenonium intermediate that can be intercepted by an external nucleophile is a well-known method to obtain 1,2-disubstituted compounds [72–74]. Therefore, the reaction of alkenes with electrophilic sources of phenylselenyl in presence of **DDPyOCF_3_** as a nucleophilic source of the CF_3_O group was studied (Table 1).

**Table 1:** Reaction of **1a** with PhSeX and **DDPyOCF_3_**.^a^

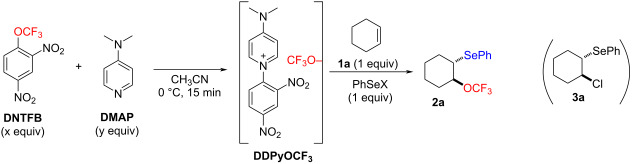					

Entry	**DNTFB** (equiv)	**DMAP** (equiv)	PhSeX	Method	**2a** (%)

1	2	1	PhSeCl	**1a** then PhSeCl; 20 °C, 24 h	12
2	3	2	PhSeCl	**1a** then PhSeCl; 20 °C, 24 h	30
3	4	3	PhSeCl	**1a** then PhSeCl; 20 °C, 24 h	32
4	3	2	PhSeCl	**1a** then PhSeCl; 0 °C, 24 h	30
5	3	2	PhSeCl	1) PhSeCl, 15 min, 0 °C 2) **1a**; 20 °C, 24 h	44
6	3	2	PhSeBr	1) PhSeBr, 15 min, 0 °C 2) **1a**; 20 °C, 24 h	93
7	2	2	PhSeBr	1) PhSeBr, 15 min, 0 °C 2) **1a**; 20 °C, 24 h	85
8	2	2	PhSeBr	1) PhSeBr, 15 min, 0 °C 2) **1a**; 20 °C, 2.5 h	88

^a^Yields determined by ^19^F NMR spectroscopy with PhCF_3_ as internal standard.

First, we started from the optimal conditions previously established for the trifluoromethoxylation by nucleophilic substitution, using an excess of **DNTFB** as a reservoir of CF_3_O^−^ [68]. Thus, by adding cyclohexene (**1a**) to the preformed mixture of **DNTFB** (2 equiv) and DMAP (1 equiv), followed by the addition of PhSeCl, only a low yield of the expected α-trifluoromethoxylated,β-phenylselenylated compound **2a** was observed (Table 1, entry 1). By increasing the amount of **DNTFB** and DMAP, the yield was doubled but remained low (entry 2 in Table 1) and did not evolve with a higher excess of reagents (entry 3). A reaction at lower temperature to possibly slow down the degradation of the CF_3_O^−^ anion did not improve the results (Table 1, entry 4). A better yield was obtained by adding first the phenylselenyl chloride and stirring the mixture for 15 min at 0 °C before adding **1a** (Table 1, entry 5). During all these reactions the formation of compound **3a** was observed as by-product. This compound results from the competitive opening of the transient episelenonium by the more nucleophilic chloride anion competing with the less nucleophilic CF_3_O^−^ anion.

To avoid this side reaction, the phenylselenyl chloride was replaced by phenylselenyl bromide, assuming that the bromide anion released is less nucleophilic than chloride in an aprotic solvent such as acetonitrile. Gratifyingly, an excellent yield was obtained (Table 1, entry 6) without detection of the brominated by-product. To facilitate purification, the amount of **DNTFB** was reduced to 2 equiv without significantly changing the result (Table 1, entry 7). Finally, the reaction time was reduced from 24 h to 2.5 h without affecting the yield (Table 1, entry 8).

**Scheme 1:**
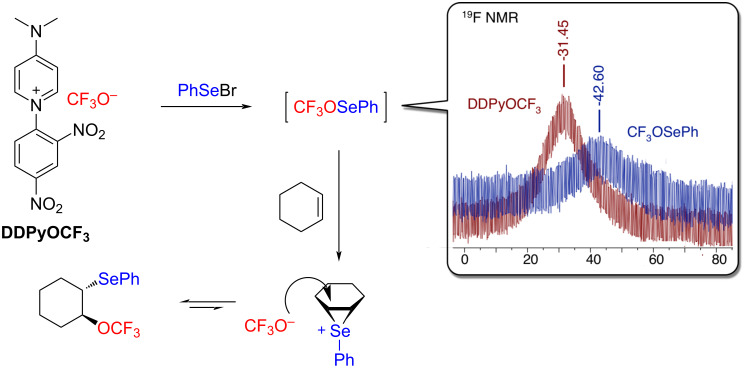
Proposed mechanism of the reaction and ^19^F NMR of the **DDPYOCF_3_**/PhSeBr mixture.

In order to better understand the mechanism of the reaction, an NMR study of the premixing of **DDPyOCF_3_** with PhSeBr was performed. The disappearance of the broad signal of CF_3_O^−^ and the appearance of a new broad signal in the upper field were observed (Scheme 1). This suggests the formation of the highly reactive CF_3_OSePh species, which cannot be isolated. Furthermore, since only the *trans* stereomer **2a** was observed, the transient formation of an episelenonium can be reasonably assumed. Consequently, the mechanism described in Scheme 1 can be proposed.

**Scheme 2:**
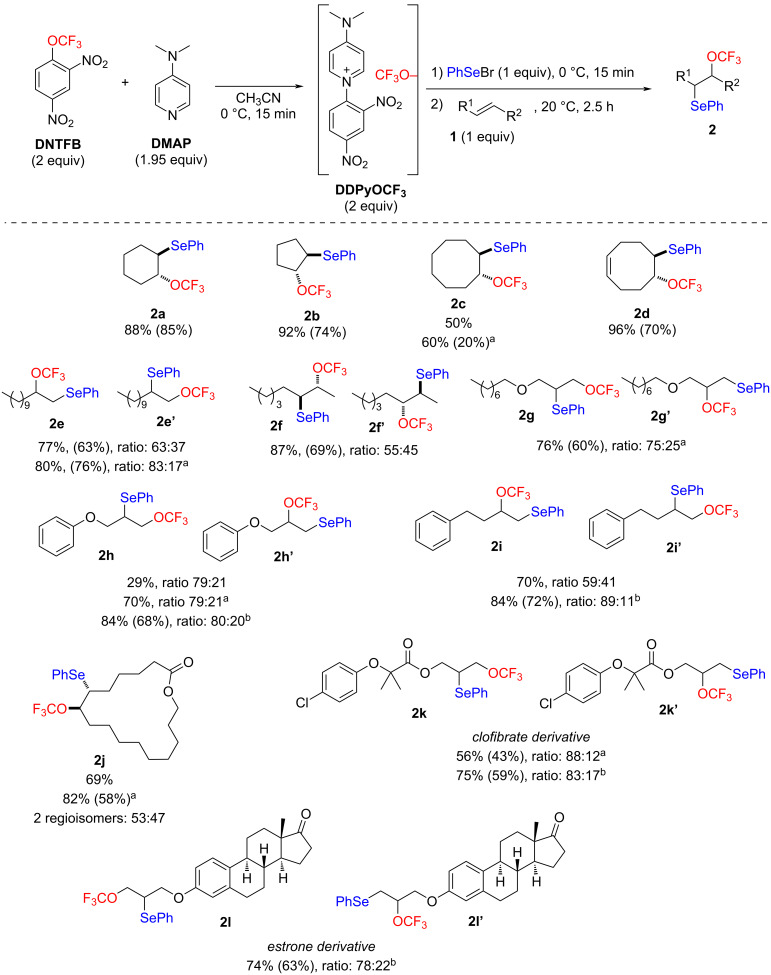
Phenylseleno trifluoromethoxylation of various alkenes. Yields determined by ^19^F NMR spectroscopy with PhCF_3_ as internal standard (in parentheses isolated yields). ^a^24 h. ^b^48 h.

Under optimal conditions (Table 1, entry 8), various alkenes were functionalized (Scheme 2).

In general, the reaction gave good yields for both cyclic (**2a**–**d**, **2j**) and aliphatic alkenes (**2e**–**2i**, **2k**, **l**). Similar results were observed regardless of the position of the double bond in the molecule. Notably, a longer reaction time was required for less reactive or more hindered substrates. The reaction was stereoselective as only the *anti* products were obtained. A good regioselectivity was generally observed, as at least 80% of the main regioisomer were usually obtained when the substituents at the double bond differed significantly. When the substituent hindrance was less pronounced, the ratio was less significant (**2f**, **2j**). Interestingly, a reverse regioselectivity was observed depending on the starting alkenes. For the terminal alkenes, the Markovnikov product (i.e. with the CF_3_O in the “internal” position) was predominant, whereas for the allylic alcohol derivatives, the anti-Markovnikov addition (i.e. with the CF_3_O in the “terminal” position) was predominant (**2e** vs **2g** and **2h** vs **2i**). This could be rationalized by the electronic effect of the oxygen atom which disfavors the episelenonium opening with the CF_3_O^−^ anion in the closest position to the oxygen atom. It is noteworthy that the regioisomeric ratio of terminal alkenes (**2e**, **2i**) evolved with the reaction time. The amount of the kinetic terminal regioisomer (anti-Markovnikov – **2e’**, **2i’**) decreases with time in favor of the thermodynamic Markovnikov regioisomer (**2e**, **2i**). This phenomenon cannot occur for products **2g** and **2h** because the kinetic and thermodynamic products are the same. This observation confirms the existence of an equilibrium between the episelenonium and the final products **2** (Scheme 1). It should be noted that the reaction with styrene gave low yields and the resulting products appeared very unstable. Finally, the tri-substituted alkene 1-methylcyclohexene did not give the expected products.

Finally, some more elaborated molecules such as the macrolactone **2j**, the clofibrate derivative **2k**, and the estrone derivative **2l** were also successfully bis-functionalized.

**Scheme 3:**
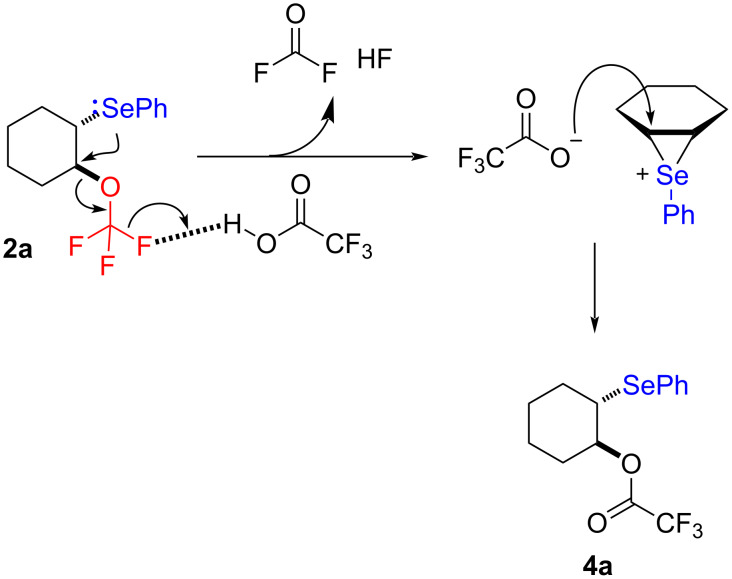
Degradation of **2a** under acidic conditions.

Some products appeared to be sensitive during purification by chromatography on silica gel. Suspecting acid sensitivity, compound **2a** was treated with trifluoroacetic acid to confirm this hypothesis (Scheme 3). The product resulting from the substitution of the CF_3_O group by the trifluoroacetoxy group was then observed. The activation of a fluorine atom from the CF_3_O group by H^+^ could be envisaged, which would then trigger the selenium attack to release difluorophosgene and HF, thus generating an episelenonium, which would finally be reopened by trifluoroacetate (Scheme 3).

Although the selenylated compounds **2** are of interest, the presence of the PhSe moiety allows other transformations to be considered. First, the oxidative elimination of the selenyl moiety to generate a double bond was first studied. However, regardless of the oxidative conditions used (mCPBA [75], H_2_O_2_ [75], selectfluor^®^/H_2_O [76], SO_2_Cl_2_/NaHCO_3_ (aq) [77–78]), in most cases a complex mixture was observed and no corresponding vinylic compound was detected by NMR.

**Scheme 4:**
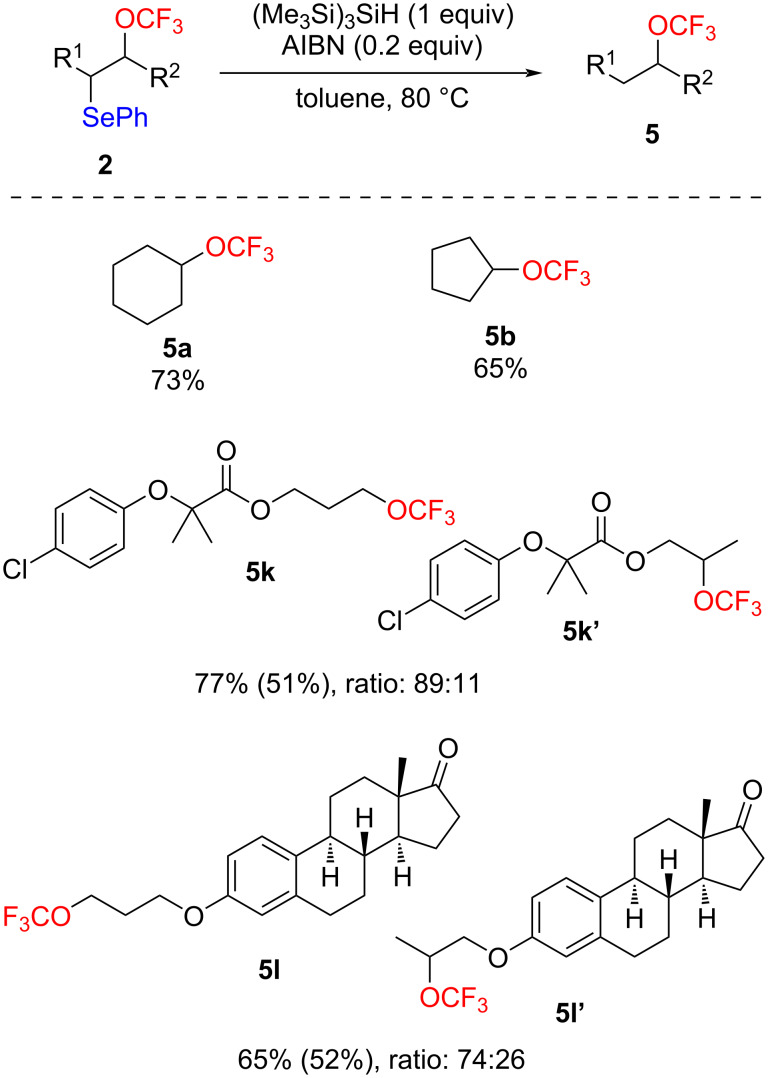
Radical deselenylation of **2**. Yields determined by ^19^F NMR spectroscopy with PhCF_3_ as internal standard (in parentheses isolated yields).

The phenylselenyl moiety could also undergo radical reduction to produce trifluoromethoxylated molecules [79]. Using tris(trimethylsilyl)silane in the presence of AIBN [80], some compounds were successfully reduced to give the corresponding trifluoromethoxylated products with good yields (Scheme 4). This approach could be a complementary method to obtain trifluoromethoxylated compounds that are difficult to synthesize by nucleophilic substitution, such as products **5a** and **5b** [68].

## Conclusion

In this work, an efficient phenylseleno trifluoromethoxylation of alkenes has been developed to readily obtain β-selenylated trifluoromethoxylated compounds. These compounds can also undergo radical deselenylation to provide trifluoromethoxylated molecules that can be difficult to access by nucleophilic substitution. These results contribute to the further valorization of the **DDPyOCF_3_** salt (arising from **DNTFB**/DMAP) as an efficient tool in organic fluorine chemistry.

## Experimental

**Typical procedure**: Synthesis of **2**. In a 10 mL vial, **DNTFB** (160 µL, 1 mmol, 2 equiv) is added in one portion to a stirred solution of **DMAP** (119 mg, 0.975 mmol, 1.95 equiv) in anhydrous MeCN (1.5 mL). The vial is closed and the reaction mixture is stirred in an ice bath for 15 minutes (the reaction rapidly turns orange after the addition of **DNTFB** and quickly turns yellow). Then, the tube is opened and PhSeBr (118 mg, 0.5 mmol, 1 equiv) is added in one portion. The resulting reaction mixture is stirred in the same ice bath for 15 minutes. Then, the tube is opened and the alkene (**1**, 0.5 mmol, 1 equiv) is added. The reaction is stirred at room temperature for 2.5 h (unless otherwise stated). Note that a yellowish precipitate is formed during the reaction for high yielding substrates. The reaction is monitored by ^19^F NMR (PhCF_3_ as internal standard). At the end of the reaction, the content of the vial is transferred to a separatory funnel and 10 mL of water are added. The aqueous layer is extracted three times with 10 mL of diethyl ether. The organic layers are combined and washed with 10 mL of water. The organic layer is dried with MgSO_4_, filtered, and concentrated under vacuum. Compounds **2** are obtained after purification by chromatography.

## Supporting Information

File 1 Additional experimental and analytical data and NMR spectra.

## Data Availability

The data that supports the findings of this study is available from the corresponding author upon reasonable request.

## References

[1] Smart B E (2001). J Fluorine Chem.

[2] Kirsch P (2013). Modern Fluoroorganic Chemistry.

[3] Szabó K J, Selander N (2021). Organofluorine Chemistry: Synthesis, Modeling, and Applications.

[4] Dolui S, Kumar D, Banerjee S, Ameduri B (2021). Acc Mater Res.

[5] Wang Y, Yang X, Meng Y, Wen Z, Han R, Hu X, Sun B, Kang F, Li B, Zhou D (2024). Chem Rev.

[6] Meanwell N A (2018). J Med Chem.

[7] Inoue M, Sumii Y, Shibata N (2020). ACS Omega.

[8] Zhang C, Yan K, Fu C, Peng H, Hawker C J, Whittaker A K (2022). Chem Rev.

[9] Sheikhi N, Bahraminejad M, Saeedi M, Mirfazli S S (2023). Eur J Med Chem.

[10] Ogawa Y, Tokunaga E, Kobayashi O, Hirai K, Shibata N (2020). iScience.

[11] Jeschke P (2022). Eur J Org Chem.

[12] Ma J A, Cahard D (2020). Emerging Fluorinated Motifs: Synthesis, Properties and Applications.

[13] Huheey J E (1965). J Phys Chem.

[14] Hansch C, Leo A, Taft R W (1991). Chem Rev.

[15] Leo A, Hansch C, Elkins D (1971). Chem Rev.

[16] Hansch C, Leo A, Unger S H, Kim K H, Nikaitani D, Lien E J (1973). J Med Chem.

[17] Taft R W, Price E, Fox I R, Lewis I C, Andersen K K, Davis G T (1963). J Am Chem Soc.

[18] Taft R W, Price E, Fox I R, Lewis I C, Andersen K K, Davis G T (1963). J Am Chem Soc.

[19] Shishkov I F, Khristenko L V, Vilkov L V, Oberhammer H (2004). J Phys Chem A.

[20] Manteau B, Genix P, Brelot L, Vors J-P, Pazenok S, Giornal F, Leuenberger C, Leroux F R (2010). Eur J Org Chem.

[21] Kang L, Novick S E, Gou Q, Spada L, Vallejo-López M, Caminati W (2014). J Mol Spectrosc.

[22] Liu J, Lin W, Sorochinsky A E, Butler G, Landa A, Han J, Soloshonok V A (2022). J Fluorine Chem.

[23] Bensimon G, Lacomblez L, Meininger V (1994). N Engl J Med.

[24] Fang T, Al Khleifat A, Meurgey J-H, Jones A, Leigh P N, Bensimon G, Al-Chalabi A (2018). Lancet Neurol.

[25] Stover C K, Warrener P, VanDevanter D R, Sherman D R, Arain T M, Langhorne M H, Anderson S W, Towell J A, Yuan Y, McMurray D N (2000). Nature.

[26] Conradie F, Diacon A H, Ngubane N, Howell P, Everitt D, Crook A M, Mendel C M, Egizi E, Moreira J, Timm J (2020). N Engl J Med.

[27] Buonamici S, Williams J, Morrissey M, Wang A, Guo R, Vattay A, Hsiao K, Yuan J, Green J, Ospina B (2010). Sci Transl Med.

[28] Pan S, Wu X, Jiang J, Gao W, Wan Y, Cheng D, Han D, Liu J, Englund N P, Wang Y (2010). ACS Med Chem Lett.

[29] Migden M R, Guminski A, Gutzmer R, Dirix L, Lewis K D, Combemale P, Herd R M, Kudchadkar R, Trefzer U, Gogov S (2015). Lancet Oncol.

[30] European Food Safety Authority (2017). EFSA Supporting Publ.

[31] EFSA Panel on Dietetic Products, Nutrition and Allergies (2014). EFSA J.

[32] Hoover G C, Seferos D S (2019). Chem Sci.

[33] Fan B, Lin F, Wu X, Zhu Z, Jen A K-Y (2021). Acc Chem Res.

[34] Rayman M P (2000). Lancet.

[35] Brown K M, Arthur J R (2001). Public Health Nutr.

[36] Singh N, Halliday A C, Thomas J M, Kuznetsova O V, Baldwin R, Woon E C Y, Aley P K, Antoniadou I, Sharp T, Vasudevan S R (2013). Nat Commun.

[37] Mousa R, Notis Dardashti R, Metanis N (2017). Angew Chem, Int Ed.

[38] Rocha J B T, Piccoli B C, Oliveira C S (2017). ARKIVOC.

[39] Jain V K, Priyadarsini K I (2017). Organoselenium Compounds in Biology and Medicine: Synthesis, Biological and Therapeutic Treatments.

[40] Chen Z, Lai H, Hou L, Chen T (2020). Chem Commun.

[41] Chuai H, Zhang S-Q, Bai H, Li J, Wang Y, Sun J, Wen E, Zhang J, Xin M (2021). Eur J Med Chem.

[42] Morán-Serradilla C, Plano D, Sanmartín C, Sharma A K (2024). J Med Chem.

[43] Li Q, Zhang Y, Chen Z, Pan X, Zhang Z, Zhu J, Zhu X (2020). Org Chem Front.

[44] Tang P, Jiang X, Cahard D, Ma J A (2020). Reagents for Direct Trifluoromethoxylation. Emerging Fluorinated Motifs.

[45] Toulgoat F, Liger F, Billard T, Szabó K, Selander N (2021). Chemistry of OCF3, SCF3, and SeCF3 Functional Groups. Organofluorine Chemistry: Synthesis, Modeling, and Applications.

[46] Hao B-Y, Han Y-P, Zhang Y, Liang Y-M (2023). Org Biomol Chem.

[47] Jelier B J, Tripet P F, Pietrasiak E, Franzoni I, Jeschke G, Togni A (2018). Angew Chem, Int Ed.

[48] Zheng W, Morales-Rivera C A, Lee J W, Liu P, Ngai M-Y (2018). Angew Chem, Int Ed.

[49] Zheng W, Lee J W, Morales-Rivera C A, Liu P, Ngai M-Y (2018). Angew Chem, Int Ed.

[50] Lee J W, Lee K N, Ngai M-Y (2019). Angew Chem, Int Ed.

[51] Lee J W, Lim S, Maienshein D N, Liu P, Ngai M-Y (2020). Angew Chem, Int Ed.

[52] Dix S, Golz P, Schmid J R, Riedel S, Hopkinson M N (2021). Chem – Eur J.

[53] Duhail T, Bortolato T, Mateos J, Anselmi E, Jelier B, Togni A, Magnier E, Dagousset G, Dell’Amico L (2021). Org Lett.

[54] Maas L M, Fasting C, Voßnacker P, Limberg N, Golz P, Müller C, Riedel S, Hopkinson M N (2024). Angew Chem, Int Ed.

[55] Billard T (2016). Methanesulfonic Acid, 1,1,1-Trifluoro-, Trifluoromethyl Ester. Encyclopedia of Reagents for Organic Synthesis.

[56] Zhou M, Ni C, Zeng Y, Hu J (2018). J Am Chem Soc.

[57] Lei M, Miao H, Wang X, Zhang W, Zhu C, Lu X, Shen J, Qin Y, Zhang H, Sha S (2019). Tetrahedron Lett.

[58] Li Y, Yang Y, Xin J, Tang P (2020). Nat Commun.

[59] Newton J J, Jelier B J, Meanwell M, Martin R E, Britton R, Friesen C M (2020). Org Lett.

[60] Turksoy A, Scattolin T, Bouayad-Gervais S, Schoenebeck F (2020). Chem – Eur J.

[61] Jiang X, Tang P (2021). Chin J Chem.

[62] Lu Z, Kumon T, Hammond G B, Umemoto T (2021). Angew Chem, Int Ed.

[63] Saiter J, Guérin T, Donnard M, Panossian A, Hanquet G, Leroux F R (2021). Eur J Org Chem.

[64] Chen L-Y, Pan P-F, Lin J-H, Jin C-M, Xiao J-C (2023). J Org Chem.

[65] Yuan W-J, Tong C-L, Xu X-H, Qing F-L (2023). J Org Chem.

[66] Bonnefoy C, Chefdeville E, Panosian A, Hanquet G, Leroux F R, Toulgoat F, Billard T (2021). Chem – Eur J.

[67] Duran-Camacho G, Ferguson D M, Kampf J W, Bland D C, Sanford M S (2021). Org Lett.

[68] Bonnefoy C, Panossian A, Hanquet G, Leroux F R, Toulgoat F, Billard T (2023). Chem – Eur J.

[69] Bonnefoy C, Chefdeville E, Tourvieille C, Panossian A, Hanquet G, Leroux F, Toulgoat F, Billard T (2022). Chem – Eur J.

[70] Bonnefoy C, Gallego A, Delobel C, Raynal B, Decourt M, Chefdeville E, Hanquet G, Panossian A, Leroux F R, Toulgoat F (2024). Eur J Org Chem.

[71] Wisson L, Hanquet G, Toulgoat F, Billard T, Panossian A, Leroux F R (2024). Eur J Org Chem.

[72] Paulmier C (1986). Selenium Reagents & Intermediates in Organic Synthesis.

[73] Krief A, Abel E W, Stone F G A, Wilkinson G (1995). Selenium. Comprehensive Organometallic Chemistry II.

[74] Preedy V R (2015). Selenium: Chemistry, Analysis, Function and Effects.

[75] Billard T, Langlois B R (1999). Tetrahedron.

[76] Guo X, Sun X, Jiang M, Zhao Y (2022). Synthesis.

[77] Engman L (1987). J Org Chem.

[78] Engman L (1987). Tetrahedron Lett.

[79] Renaud P, Wirth T (2000). Radical Reactions Using Selenium Precursors. Organoselenium Chemistry: Modern Developments in Organic Synthesis.

[80] Ballestri M, Chatgilialoglu C, Clark K B, Griller D, Giese B, Kopping B (1991). J Org Chem.

