# Supplementary material for: Phenylseleno trifluoromethoxylation of alkenes
Source: Beilstein J Org Chem. 2024 Sep 26;20:2434–41. doi: 10.3762/bjoc.20.207 (PMC11443662; doi:10.3762/bjoc.20.207)
Supplement: File 1 — Additional experimental and analytical data and NMR spectra. [file Beilstein_J_Org_Chem-20-2434-s001.pdf]

BEILSTEIN JOURNAL OF ORGANIC CHEMISTRY

Supporting Information

for

Phenylseleno trifluoromethoxylation of alkenes

Clément Delobel, Armen Panossian, Gilles Hanquet, Frédéric R. Leroux, Fabien Toulgoat and Thierry Billard

Beilstein J. Org. Chem. **2024,** *20,* 2434–2441. doi:10.3762/bjoc.20.207

Additional experimental and analytical data and NMR spectra

License and Terms: This is a supporting information file under the terms of the Creative Commons Attribution License (https://creativecommons.org/licenses/by/4.0). Please note that the reuse, redistribution and reproduction in particular requires that the author(s) and source are credited and that individual graphics may be subject to special legal provisions.
The license is subject to the *Beilstein Journal of Organic Chemistry* terms and conditions: (https://www.beilstein-journals.org/bjoc/terms)

Table of contents

General information S3

General procedures S3

Procedure A S3

Purification of compounds **2** on alumina S4

Purification of compounds **2** on silica S4

Procedure B S4

Procedure C: preparation of the clofibrate derivative **1k** S4

Procedure D: preparation of the estrone derivative **1l** S4

Procedure E S4

Products characterization S5

Allyl 2-(4-chlorophenoxy)-2-methylpropanoate (**1k**) S5

(8*R*,9*S*,13*S*,14*S*)-3-(Allyloxy)-13-methyl-6,7,8,9,11,12,13,14,15,16-decahydro-17*H*-cyclopenta[*a*]phenanthren-17-one (**1l**) S5

Phenyl(2-(trifluoromethoxy)cyclohexyl)selane (**2a**) S5

Phenyl(2-(trifluoromethoxy)cyclopentyl)selane (**2b**) S6

Phenyl(2-(trifluoromethoxy)cyclooctyl)selane (**2c**) S6

Phenyl(Z)-8-(trifluoromethoxy)cyclooct-4-en-1-yl)selane (**2d**) S6

Phenyl(2-(trifluoromethoxy)dodecyl)selane (**2e**) S7

Phenyl(1-(trifluoromethoxy)dodecan-2-yl)selane (**2e'**) S7

Phenyl(2-(trifluoromethoxy)octan-3-yl)selane (**2f**) S7

Phenyl(3-(trifluoromethoxy)octan-2-yl)selane (**2f'**) S7

(1-((2-Methylheptyl)oxy)-3-(trifluoromethoxy)propan-2-yl)(phenyl)selane (**2g**) S8

(3-((2-Methylheptyl)oxy)-2-(trifluoromethoxy)propyl)(phenyl)selane (**2g'**) S8

(1-Phenoxy-3-(trifluoromethoxy)propan-2-yl)(phenyl)selane (**2h**) S8

(3-Phenoxy-2-(trifluoromethoxy)propyl)(phenyl)selane (**2h'**) S8

Phenyl(4-phenyl-2-(trifluoromethoxy)butyl)selane (**2i**) S9

Phenyl(4-phenyl-1-(trifluoromethoxy)butan-2-yl)selane (**2i'**) S9

7-(Phenylselanyl)-8-(trifluoromethoxy)-1-oxacycloheptadecan-2-one (**2j**/**2j'**) S9

8-(Phenylselanyl)-7-(trifluoromethoxy)-1-oxacycloheptadecan-2-one (**2j**/**2j'**) S9

2-(Phenylselanyl)-3-(trifluoromethoxy)propyl 2-(4-chlorophenoxy)-2-methylpropanoate (**2k**) S10

3-(Phenylselanyl)-2-(trifluoromethoxy)propyl 2-(4-chlorophenoxy)-2-methylpropanoate (**2k'**) S10

(3a*S*,3b*R*,9b*S*,11a*S*)-11a-Methyl-7-[2-(phenylselanyl)-3-(trifluoromethoxy)propoxy]-1*H*,2*H*,3*H*,3a*H*,3b*H*,4*H*,5*H*,9b*H*,10*H*,11*H*,11a*H*-cyclopenta[*a*]phenanthren-1-one (**2l**) S10

(3a*S*,3b*R*,9b*S*,11a*S*)-11a-Methyl-7-[3-(phenylselanyl)-2-(trifluoromethoxy)propoxy]-1*H*,2*H*,3*H*,3a*H*,3b*H*,4*H*,5*H*,9b*H*,10*H*,11*H*,11a*H*-cyclopenta[*a*]phenanthren-1-one (**2l'**) S10

[(2-Chlorocyclohexyl)selanyl]benzene (**3a**) S11

2-(Phenylselanyl)cyclohexyl 2,2,2-trifluoroacetate (**4a**) S11

3-(Trifluoromethoxy)propyl 2-(4-chlorophenoxy)-2-methylpropanoate (**5k**) S11

2-(Trifluoromethoxy)propyl 2-(4-chlorophenoxy)-2-methylpropanoate (**5k'**) S11

(3b*R*,9b*S*,11a*S*)-11a-Methyl-7-[3-(trifluoromethoxy)propoxy]-1*H*,2*H*,3*H*,3a*H*,3b*H*,4*H*,5*H*,9b*H*,10*H*,11*H*,11a*H*-cyclopenta[*a*]phenanthren-1-one (**5l**) S12

(3b*R*,9b*S*,11a*S*)-11a-Methyl-7-[2-(trifluoromethoxy)propoxy]-1*H*,2*H*,3*H*,3a*H*,3b*H*,4*H*,5*H*,9b*H*,10*H*,11*H*,11a*H*-cyclopenta[*a*]phenanthren-1-one (**5l'**) S12

References S13

NMR Spectra S14

General information

All reactions were carried out in a sealed vial under a nitrogen atmosphere with dry solvents under anhydrous conditions, unless otherwise noted. Acetonitrile (MeCN, 99.8%, anhydrous in a Sure/SealTM bottle), dimethylformamide (DMF anhydrous, >99.8%), and toluene (THF anhydrous, >99.9%, inhibitor free) were purchased from Merck Sigma Aldrich.
Reagents and solvents were purchased at commercial quality available and used without further purification, unless otherwise stated.
Thin-layer chromatography analyses were realized using ALUGRAM SIL G/UV254, 0.20 mm on an aluminium support (Macherey-Nagel) and revealed with UV irradiation (254 nm). Flash chromatography purifications were made with Silica 60M, 0.04–0.063 mm supplied from Macherey-Nagel Kieselgel.
NMR spectra were recorded on a Bruker AV 500 spectrometer at 500 MHz (^{1}H NMR), 126 MHz (^{13}C NMR), 471 MHz (^{19}F NMR), 95 MHz (^{77}Se), on a Jeol ECZ400 spectrometer at 400 MHz (^{1}H NMR), 101 MHz (^{13}C NMR), 376 MHz (^{19}F NMR), 76 MHz (^{77}Se), or on a Bruker AV 300 spectrometer at 300 MHz (^{1}H NMR), 282 MHz (^{19}F NMR). Coupling constants (J) are reported in hertz (Hz), and the following abbreviations were used to designate multiplicities: s = singlet, d = doublet, t = triplet, q= quartet, quint = quintet, m = multiplet, br = broad.
^{13}C NMR spectra and ^{77}Se NMR spectra were recorded with complete proton decoupling. Chemical shifts for ^{1}H and ^{13}C are reported in ppm from tetramethylsilane. Chemical shifts for ^{19}F NMR spectra are recorded in ppm from $CFCl_3$.
Assignments of NMR signals were made by homonuclear (COSY) and heteronuclear (HSQC, HMBC, and ^{19}F gc2HSQC) two-dimensional correlation spectroscopy
Melting points were determined using a Kofler bench apparatus (calibration substances were specified).
HRMS measurements were performed on a Bruker MicrOTOFQ II (electrospray ionization, ESI) and an Agilent 7200 GC/Q-TOF (electron ionization, EI) mass spectrometers.
Caution: Reactions involving **DNTFB** (2,4-dinitro-1-(trifluoromethoxy)benzene) may generate difluorophosgene, which is a very toxic gas. As such, reactions must be carried out in a well-ventilated fume hood.

General procedures

Procedure A

In a 10 mL vial, **DNTFB** (160 µL, 1 mmol, 2 equiv) was added in one portion into a stirred solution of **DMAP** (119 mg, 0.975 mmol, 1.95 equiv) in anhydrous MeCN (1.5 mL). The vial is closed and the reaction is stirred in an ice bath for 15 minutes (of note, the reaction quickly becomes orange after **DNTFB** addition and quickly turns yellow). Then, the tube was opened and **PhSeBr** (118 mg, 0.5 mmol, 1 equiv) is added in one portion. The reaction mixture is stirred in the same ice bath for 15 minutes. Then, the tube is opened and the alkene **1** is added (0.5 mmol, 1 equiv). The reaction mixure is stirred at room temperature for 2.5 h (unless otherwise stated). Of note, a yellowish precipitate was formed during the reaction for high-yielding substrates. The yield of the reaction was determined by ^{19}F NMR using trifluoromethylbenzene as an internal standard. After the end of the reaction, the content of the vial is transferred to a separatory funnel and 10 mL of water are added. The aqueous layer is extracted three times with 10 mL of diethyl ether. The organic layers are combined and washed with 10 mL of water. The organic layer is dried using $MgSO_4$, filtered, and then concentrated under vacuum. Two different purification processes can be used to get the pure compounds. Compounds **2** are obtained after purification.

Purification of compounds 2 on alumina

For the purification process, a mix of 3.5 mL pentane and 0.5 mL DCM is added to the crude in a flask (an ultrasonic bath can be used to obtain a homogenous solution). The mix is collected using a syringe. The needle of the syringe is removed, and the syringe is fixed to an aluminium oxide cartridge (Waters Sep-Pak Alumina B Plus Long Cartridge) for filtration. The crude flask is rinsed using 4 mL pentane and the same process is repeated using the same cartridge. The filtrate obtained is concentrated under vacuum. The isolated compound is stored under nitrogen in a fridge.

Purification of compounds 2 on silica

Before the flash chromatography, the silica underwent a basic treatment. In a large beaker, a suspension of silica in diethyl ether is prepared. Then, Et_3N is added to the beaker (about 5% of the volume of diethyl ether) and the mixture is stirred for approximatively 2 hours. The silica is then filtered and washed abundantly with diethyl ether. The silica is then dried and can be easily stored and used for future flash chromatography.

Procedure B

In a 10 mL vial, compound **2** (mixture of regioisomers, 0.3 mmol, 1 equiv) is dissolved in anhydrous toluene (1.5 mL). The reaction vial is flushed with N_2 and degassed. AIBN (10 mg, 0.06 mmol, 0.2 equiv) and $(TMS)_3SiH$ (95 µL, 0.3 mmol, 1 equiv) are added to the vial. The reaction mixture is stirred at 80 °C for 2.5 h. The yield of the reaction was determined by ^{19}F NMR using trifluoromethylbenzene as an internal standard. For non-volatile compounds (**5k** and **5l**), water (10 mL) is added to the vial and the reaction mixture is transferred to a separatory funnel. The aqueous layer is extracted with diethyl ether (3 × 10 mL). The organic layers are combined, dried using $MgSO_4$, filtered, and concentrated under vacuum. The crude mixture is purified by flash chromatography.

Procedure C: preparation of the clofibrate derivative 1k

The clofibrate derivative was prepared according to a procedure described in the literature[1]. Allyl bromide (2.8 mL, 32 mmol, 8 equiv) was slowly added to a solution of clofibric acid (854 mg, 4 mmol, 1 equiv) and $NaHCO_3$ (1.3 g, 16 mmol, 4 equiv) in dry DMF. The reaction mixture was stirred at room temperature for 24 h. The mixture was then washed with water and extracted three times with DCM. The organic layers were combined, dried using $MgSO_4$, filtered, and concentrated under vacuum.

Procedure D: preparation of the estrone derivative 1l

The estrone derivative was prepared according to a procedure described in the literature[2]. Estrone (811 mg, 3 mmol, 1 equiv) was dissolved in 45 mL of acetone. Then, allyl bromide (520 µL, 6 mmol, 2 equiv) and K_2CO_3 (2.9 g, 21 mmol, 7 equiv) are added under stirring. The reaction mixture was stirred at 50 °C for 48 h. The acetone is evaporated under vacuum. Water is added and the aqueous layer is extracted three times with DCM. The organic layers were combined, dried with $MgSO_4$, filtered, and concentrated under vacuum. The crude product is purified by flash chromatography.

Procedure E

In a 10 mL vial, compound **2a** (135 mg, 0.39 mmol, 1 equiv) is dissolved in anhydrous DCM (0.5 mL). Then, trifluoroacetic acid (40 µL, 0.52 mmol, 1.3 equiv) is added and the mixture is stirred at room temperature for 16 h and analyzed by ^{19}F NMR with $PhCF_3$ as an internal standard. The mixture is then concentrated under vacuum using a rotary evaporator. The resulting product was analyzed by ^{19}F NMR and by ^{1}H NMR. Compound **4a** was obtained in accordance with literature data. [3]

Products characterization

Allyl 2-(4-chlorophenoxy)-2-methylpropanoate (1k)

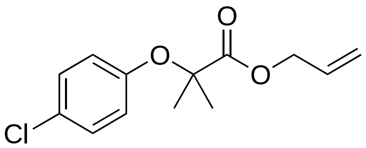

Procedure C
Light-yellow liquid, 696 mg, 68%
^{1}H NMR (300 MHz, $CDCl_3$) δ 7.19 (m, 2H), 6.79 (m, 2H), 5.87 (m, 1H), 5.28 (m, 2H), 4.66 (dt, $J_{H\text{-}H}$ = 5.8, 5.8, 1.4 Hz, 2H), 1.59 (s, 6H).
^{13}C NMR (101 MHz, $CDCl_3$) δ 173.68, 154.05, 131.52, 129.20, 127.42, 120.81, 119.11, 79.64, 66.07, 25.40.
HRMS (ESI): Calc. for [M+Na^+] $C_{13}H_{15}ClNaO_3$: 277.0602, measured : 277.0607

(8*R*,9*S*,13*S*,14*S*)-3-(Allyloxy)-13-methyl-6,7,8,9,11,12,13,14,15,16-decahydro-17*H*-cyclopenta[*a*]phenanthren-17-one (1l)

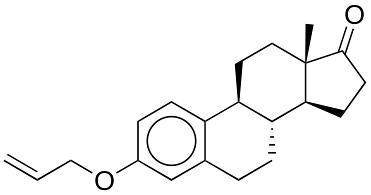

Procedure D
White solid, 747 mg, 80%
Eluant for flash chromatography: 100%DCM
^{1}H NMR (300 MHz, $CDCl_3$) δ 7.20 (dd, $J_{H\text{-}H}$ = 8.6 Hz, 1.1 Hz, 1H), 6.73 (dd, $J_{H\text{-}H}$ = 8.6 Hz, 2.8 Hz, 1H), 6.66 (dd, $J_{H\text{-}H}$ = 2.7 Hz, 1.1 Hz, 1H), 6.05 (ddt, $J_{H\text{-}H}$ = 17.3, 10.5, 5.3 Hz, 1H), 5.40 (ddt, $J_{H\text{-}H}$ = 17.3 Hz, 1.6 Hz, 1.6Hz, 1H), 5.27 (ddt, $J_{H\text{-}H}$ = 10.5 Hz, 1.6 Hz, 1.6 Hz, 1H), 4.51 (dt, $J_{H\text{-}H}$ = 5.3 Hz, 1.6 Hz, 2H), 3.01 – 2.84 (massif, 2H), 2.62 – 1.91 (massif, 7H), 1.75 – 1.34 (massif, 6H), 0.91 (s, 3H).
The results are in accordance with the literature[2]

Phenyl(2-(trifluoromethoxy)cyclohexyl)selane (2a)

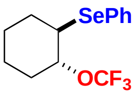

Procedure A
Purification on alumina
Light-yellow oil, 136 mg, 85%
^{19}F NMR (376 MHz, $CDCl_3$) δ -57.35 (s).
^{1}H NMR (300 MHz, $CDCl_3$) δ 7.58 (m, 2H), 7.35 – 7.24 (massif, 3H), 4.31 (ddd, $J_{H\text{-}H}$ = 7.3, 7.3, 3.6 Hz, 1H), 3.37 (ddd, $J_{H\text{-}H}$ = 7.3, 7.3, 4.1 Hz, 1H), 2.20 – 2.16 (massif, 2H), 1.82 – 1.52 (massif, 4H), 1.50 – 1.33 (massif, 2H).
^{13}C NMR (101 MHz, $CDCl_3$) δ 134.81, 129.11, 128.53, 127.85, 121.60 (q, $J_{C\text{-}F}$ = 254.6 Hz, Cq), 79.49, 45.31, 30.37, 30.29, 24.04, 22.10.
HRMS (EI): calc. for [$M^{+\bullet}$] [$C_{13}H_{15}F_3OSe$]: 324.0235, measured 324.0221

Phenyl(2-(trifluoromethoxy)cyclopentyl)selane (2b)

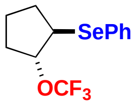

Procedure A
Purification on alumina
Light-yellow oil, 116 mg, 74%
^{19}F NMR (376 MHz, $CDCl_3$) δ -58.01 (s).
^{1}H NMR (300 MHz, $CDCl_3$) δ 7.56 (m, 2H), 7.36 – 7.27 (massif, 3H), 4.64 (ddd, J_{H-H} = 5.3, 2.3, 2.3 Hz, 1H), 3.74 (m, 1H), 2.34 – 2.13 (massif, 2H), 1.90 – 1.62 (massif, 4H).
^{13}C NMR (101 MHz, $CDCl_3$) δ 134.3, 129.34, 128.88, 128.02, 121.50 (q, J_{C-F} = 255.1 Hz), 85.81 (q, J_{C-F} = 2.2 Hz), 46.14, 31.21, 30.69, 22.64.
^{77}Se NMR (76 MHz, $CDCl_3$) δ 366.51 (s).
HRMS (EI): calc. for [$M^{+\bullet}$] [C12H13F3OSe]: 310.0079, measured 310.0079

Phenyl(2-(trifluoromethoxy)cyclooctyl)selane (2c)

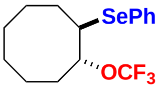

Procedure A
Purification on treated silica
Eluent for flash chromatography:100% pentane
Light-yellow oil, 36 mg, 20% (24h)
^{19}F NMR (376 MHz, $CDCl_3$) δ -57.38 (s).
^{1}H NMR (300 MHz, $CDCl_3$) δ 7.55 (m, 2H), 7.28 – 7.22 (massif, 3H), 4.48 (ddd, J_{H-H} = 8.7, 6.4, 2.5 Hz, 1H), 3.51 (ddd, J_{H-H} = 8.7, 7.9, 2.6 Hz, 1H), 2.20 – 1.93 (massif, 3H), 1.87 – 1.30 (massif, 9H).
^{13}C NMR (101 MHz, $CDCl_3$) δ 134.93, 129.49, 129.19, 127.85, 121.67 (q, J_{C-F} = 255.0 Hz), 83.13 (q, J_{C-F} = 1.8 Hz), 48.71, 30.78 5, 28.77, 26.19, 25.98, 25.68, 23.92.
^{77}Se NMR (76 MHz, $CDCl_3$) δ 386.46.
HRMS (APCI): Calc. for [$M^{+\bullet}$] [$C_{15}H_{19}F_3OSe$]: 352.0548, measured: 352.0550

Phenyl(*Z*)-8-(trifluoromethoxy)cyclooct-4-en-1-yl)selane (2d)

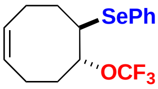

Procedure A
Purification on alumina
Light-yellow oil, 120 mg, 70%
^{19}F NMR (376 MHz, $CDCl_3$) δ -57.98 (s).
^{1}H NMR (300 MHz, $CDCl_3$) δ 7.55 (m, 2H), 7.32 – 7.24 (massif, 3H), 5.79 – 5.63 (massif, 2H), 4.60 (ddd, J_{H-H} = 8.3, 6.6, 3.1 Hz, 1H), 3.72 (ddd, J_{H-H} = 8.3, 8.3, 3.4 Hz, 1H), 2.48 – 2.13 (massif, 6H), 2.10 – 1.93 (massif, 2H).
^{13}C NMR (101 MHz, $CDCl_3$) δ 134.46, 130.51, 130.33, 129.56, 129.27, 127.78, 121.64 (q, J_{C-F} = 254.7 Hz), 81.24 (d, J_{C-F} = 2.0 Hz), 46.90, 33.38, 32.96, 25.14, 22.34.
^{77}Se NMR (76 MHz, $CDCl_3$) δ 381.00 (q, J = 5.7 Hz)
HRMS (ESI): calc. for [$M+H^{+}$] [$C_{15}H_{18}F_3OSe$]: 350.0397, measured 350.0472

Phenyl(2-(trifluoromethoxy)dodecyl)selane (2e)

Phenyl(1-(trifluoromethoxy)dodecan-2-yl)selane (2e')

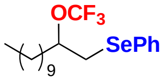

2e

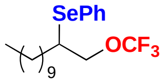

2e'

Procedure A
Purification on alumina
Light-yellow oil, 155 mg, 76%, Ratio **2e**/**2e'**: 83/17 (24h)
^{19}F NMR (376 MHz, $CDCl_3$) δ -57.65 (s, **2e**, 0.83), -60.30 (s, **2e'**, 0.17).
^{1}H NMR (400 MHz, $CDCl_3$) δ 7.58 – 7.49 (massif, 2H), 7.32 – 7.26 (massif, 3H), 4.32 (dddd, $J_{H\text{-}H}$ = 7.9, 7.9, 4.6, 4.6 Hz, 0.83H), 4.13 (dd, $J_{H\text{-}H}$ = 10.0 Hz, 4.7 Hz, 0.17H), 3.98 (dd, $J_{H\text{-}H}$ = 10.0 Hz, 8.9 Hz, 0.17H), 3.28 (dddd, $J_{H\text{-}H}$ = 8.9, 8.9, 4.7, 4.7 Hz, 0.17H), 3.20 (dd, $J_{H\text{-}H}$ = 12.8 Hz, 4.6 Hz, 0.83H), 3.06 (dd, $J_{H\text{-}H}$=12.8 Hz, 7.9 Hz, 0.83H), 1.90 – 1.77 (massif, 1H), 1.75 – 1.60 (massif, 1H), 1.34 – 1.18 (massif, 16H), 0.92 – 0.86 (massif, 3H).
^{13}C NMR (101 MHz, $CDCl_3$) δ 135.29, 133.18, 129.40, 129.37, 128.29, 127.71, 127.62, 121.76 (q, $J_{C\text{-}F}$ = 254.8 Hz), 79.15 (q, $J_{C\text{-}F}$ = 1.9 Hz), 70.05 (q, $J_{C\text{-}F}$ = 3.1 Hz), 42.91, 33.69, 32.05, 31.57, 31.34, 29.78 – 29.23 (massif), 27.48, 24.69, 22.84, 14.27.
^{77}Se NMR (76 MHz, $CDCl_3$) δ 337.89 (s, **2e'**, 0.17), 264.44 (s, **2e**, 0.83).
HRMS (EI): calc. for [$M^{+\bullet}$] [$C_{19}H_{29}F_3OSe$]: 410.1331, measured 410.1337

Phenyl(2-(trifluoromethoxy)octan-3-yl)selane (2f)

Phenyl(3-(trifluoromethoxy)octan-2-yl)selane (2f')

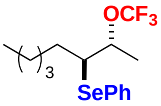

2f

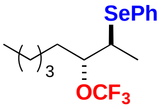

2f'

Procedure A
Purification on treated silica, eluent for flash chromatography: Pentane
Colorless oil, 125 mg, 69%, Ratio **2f**/**2f'**: 55/45
^{19}F NMR (376 MHz, $CDCl_3$) δ -57.00 (s, **2f'**, 0.45), -57.69 (s, **2f**, 0.55).
^{1}H NMR (300 MHz, $CDCl_3$) δ 7.65 – 7.53 (massif, 2H), 7.34 – 7.24 (massif, 3H), 4.44 (m, 0.55H), 4.26 (ddd, $J_{H\text{-}H}$ = 7.1, 5.1, 5.1 Hz, 0.45H), 3.44 (qd, $J_{H\text{-}H}$ = 7.1, 4.8 Hz, 0.45H), 3.17 (ddd, $J_{H\text{-}H}$ = 9.5, 5.4, 4.1 Hz, 0.55H), 1.86 – 1.21 (massif, 11H), 0.92-0.86 (massif, 3H).
^{13}C NMR (101 MHz, $CDCl_3$) δ 135.48, 135.15, 129.25, 129.21, 128.70, 128.15, 127.93, 121.79 (q, $J_{C\text{-}F}$ = 254.4 Hz), 82.87 (q, $J_{C\text{-}F}$ = 1.7 Hz), 78.47 (q, $J_{C\text{-}F}$ = 2.0 Hz), 52.06, 42.28, 32.18, 31.60, 31.57, 31.54, 27.71, 24.48, 22.60, 22.57, 18.97, 17.82, 14.12, 14.05.
^{77}Se NMR (95 MHz, $CDCl_3$) δ 382.70 (s, **2f'**,0.45), 333.43 (s, **2f**, 0.55).
HRMS (EI): calc. for [$M^{+\bullet}$] [$C_{15}H_{21}F_3OSe$]: 354.0704, measured 354.0685

(1-(Octyloxy)-3-(trifluoromethoxy)propan-2-yl)(phenyl)selane (2g)

(3-(Octyloxy)-2-(trifluoromethoxy)propyl)(phenyl)selane (2g')

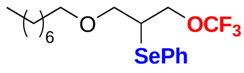

2g

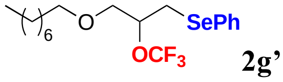

2g'

Procedure A
Purification on treated silica, eluent for flash chromatography: Pentane to Pentane/Diethylether 9/1
Light-yellow oil, 123 mg, 60%, Ratio **2g**/**2g'**: 75/25 (24h)
^{19}F NMR (282 MHz, $CDCl_3$) δ -58.17 (s, **2g'**,0.25), -60.47 (s, **2g**, 0.75).
^{1}H NMR (300 MHz, $CDCl_3$) δ 7.65 – 7.45 (massif, 2H), 7.32 – 7.22 (massif, 3H), 4.41 (m, 0.25H), 4.29 (dd, $J_{H\text{-}H}$ = 10.0, 8.0 Hz, 0.75H), 4.16 (dd, $J_{H\text{-}H}$ = 10.0, 4.8 Hz, 0.75H), 3.85 – 3.58 (massif, 2H), 3.48 – 3.11 (massif, 3.25H), 1.62 – 1.45 (massif, 2H), 1.40 – 1.14 (massif, 10H), 0.91 – 0.83 (massif, 3H).
^{13}C NMR (126 MHz, $CDCl_3$) δ 134.74, 132.95, 129.43, 129.38, 128.18, 128.16, 127.54, 121.70 (q, $J_{C\text{-}F}$ = 255.5 Hz), 121.63 (q, $J_{C\text{-}F}$ = 254.9 Hz), 77.52 (q, $J_{C\text{-}F}$ = 2.1 Hz), 71.90, 71.61, 70.26, 69.55, 67.00 (q, $J_{C\text{-}F}$ = 2.9 Hz), 42.38, 31.96, 29.64, 29.52, 29.39, 27.97, 26.19, 26.13, 22.80, 14.22.
^{77}Se NMR (76 MHz, $CDCl_3$) δ 341.35 (s, **2g**, 0.75), 264.21 (s, **2g'**, 0.25).
HRMS (ESI) : Calc. for [M+Na$^+$] $C_{18}H_{27}F_3NaO_2Se$: 435.1021, measured: 435.1015

(1-Phenoxy-3-(trifluoromethoxy)propan-2-yl)(phenyl)selane (2h)

(3-Phenoxy-2-(trifluoromethoxy)propyl)(phenyl)selane (2h')

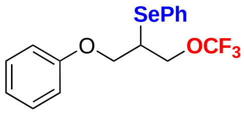

2h

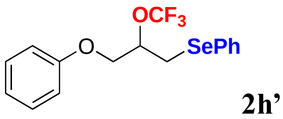

2h'

Procedure A
Purification on alumina
Colorless oil, 130 mg, 68%, Ratio **2h**/**2h'**: 80/20 (48h)
^{19}F NMR (376 MHz, $CDCl_3$) δ -58.24 (s, **2h'**, 0.21), -60.50 (s, **2h**, 0.79).
^{1}H NMR (300 MHz, $CDCl_3$) δ 7.67 – 7.48 (massif, 2H), 7.42 – 7.19 (massif, 5H), 7.11 – 6.77 (massif, 3H), 4.63 (m, 0.21H), 4.43 – 4.20 (massif, 3.58H), 3.60 (m, 0.79H), 3.32 (m, 0.42H).
^{13}C NMR (101 MHz, $CDCl_3$) δ 158.30, 158.09, 135.16, 133.09, 129.67, 129.64, 129.53, 129.47, 128.91, 128.57, 127.76, 127.51, 121.68 (q, $J_{C\text{-}F}$ = 255.9 Hz), 121.60 (q, $J_{C\text{-}F}$ = 255.6 Hz), 121.58, 121.51, 114.78, 114.70, 76.70 (q, $J_{C\text{-}F}$ = 2.1 Hz), 67.46, 66.96, 66.74 (q, $J_{C\text{-}F}$ = 3.0 Hz), 41.37, 27.80.
^{77}Se NMR (76 MHz, $CDCl_3$) δ 344.06 (s, **2h**, 0.79), 265.11 (s, **2h'**, 0.21).
HRMS (APCI): Calc. for [M+$^\bullet$] $C_{16}H_{15}F_3O_2Se$: 376.0184, measured: 376.0180

Phenyl(4-phenyl-2-(trifluoromethoxy)butyl)selane (2i)

Phenyl(4-phenyl-1-(trifluoromethoxy)butan-2-yl)selane (2i’)

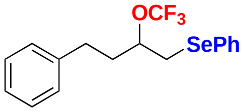

2i

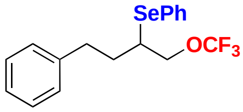

2i’

Procedure A
Purification on alumina
Light-yellow oil, 134mg, 72%, Ratio **2i**/**2i’**: 89/11 (48h)
^{19}F NMR (282 MHz, $CDCl_3$) δ -57.50 (s, **2i**, 0.89), -60.33 (s, **2i’**, 0.11).
^{1}H NMR (500 MHz, $CDCl_3$) δ 7.46 – 7.40 (massif, 2H), 7.29 – 7.05 (massif, 8H), 4.30 (m, 0.89H), 4.13 (dd, $J_{H\text{-}H}$ = 10.1, 4.7 Hz, 0.11H), 3.97 (dd, $J_{H\text{-}H}$ = 10.1, 8.8 Hz, 0.11H), 3.22 (m, 0.11H), 3.18 (dd, $J_{H\text{-}H}$ = 12.9, 4.3 Hz, 0.89H), 3.01 (dd, $J_{H\text{-}H}$ = 12.9, 8.3 Hz, 0.89H), 2.95 (m, 0.11H), 2.77 (m, 0.11H), 2.71 (m, 0.89H), 2.59 (m, 0.89H), 2.20 – 2.11 (massif, 1H), 2.00 (m, 0.89H), 1.81 (m, 0.11H).
^{13}C NMR (126 MHz, $CDCl_3$) δ 141.03, 140.77, 135.42, 133.40, 129.4, 129.06, 128.63, 128.59, 128.50, 128.42, 127.72, 126.28, 124.84, 121.80 (q, $J_{C\text{-}F}$ = 255.1 Hz), 121.62 (q, $J_{C\text{-}F}$= 255.1 Hz), 78.43 (q, $J_{C\text{-}F}$ = 1.9 Hz), 70.03 (q, $J_{C\text{-}F}$ = 2.8 Hz), 42.03, 35.23, 33.59, 32.93, 31.44, 30.94.
^{77}Se NMR (76 MHz, $CDCl_3$) δ 335.87 (s, **2i’**, 0.11), 267.01 (s, **2i**, 0.89).
HRMS (APCI) : Calc. for [M+H$^+$] [$C_{17}H_{18}F_3OSe$]: 375.0469, measured: 375.0465

7-(Phenylselanyl)-8-(trifluoromethoxy)-1-oxacycloheptadecan-2-one (2j/2j’)

8-(Phenylselanyl)-7-(trifluoromethoxy)-1-oxacycloheptadecan-2-one (2j/2j’)

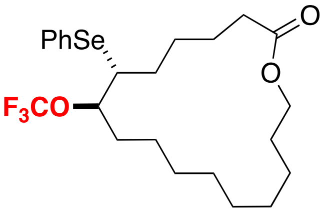

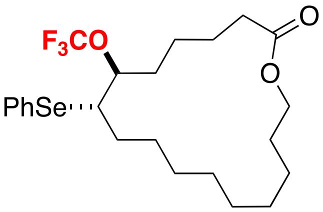

Procedure A
Purification on treated silica, eluent for flash chromatography: Pentane/Diethylether 96/4
Colorless oil, 142 mg, 58%, Ratio: 53/47 (24h)
^{19}F NMR (376 MHz, $CDCl_3$) δ -57.07 (s, 0.47), -57.23 (s, 0.53).
^{1}H NMR (400 MHz, $CDCl_3$) δ 7.62 – 7.51 (massif, 2H), 7.32 – 7.19 (massif, 3H), 4.38 – 4.31 (massif, 1H), 4.24 – 4.14 (massif, 1H), 4.07 – 3.97 (massif, 1H), 3.35 – 3.29 (massif, 1H), 2.43 – 2.20 (massif, 2H), 1.86 – 1.55 (massif, 8H), 1.52 – 1.17 (massif, 14H).
^{13}C NMR (101 MHz, $CDCl_3$) δ 173.98, 173.91, 135.05, 135.02, 129.67, 129.65, 129.19, 129.17, 127.85, 127.80, 121.81 (q, $J_{C\text{-}F}$ = 254.6 Hz), 121.80 (q, $J_{C\text{-}F}$ = 254.6 Hz), 81.15, 80.99, 64.24, 64.06, 49.95, 49.06, 34.70, 30.60, 30.30, 28.61 , 28.54, 28.38, 28.36, 28.17, 27.82, 27.79, 27.78, 27.61, 27.50, 26.65, 25.68, 25.52, 25.25, 25.15, 24.66, 23.73.
^{77}Se NMR (76 MHz, $CDCl_3$) δ 354.28 (s, 0.47), 343.14 (s, 0.53).
HRMS (APCI) : Calc. for [M+$^{\bullet}$] [$C_{23}H_{33}F_3O_3Se$]: 494.1547, measured: 494.1541

2-(Phenylselanyl)-3-(trifluoromethoxy)propyl 2-(4-chlorophenoxy)-2-methylpropanoate (2k)

3-(Phenylselanyl)-2-(trifluoromethoxy)propyl 2-(4-chlorophenoxy)-2-methylpropanoate (2k')

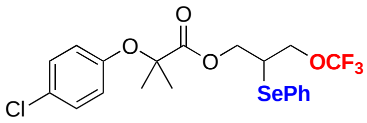

2k

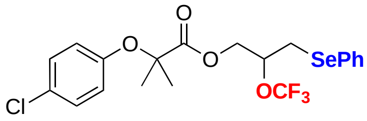

2k'

Procedure A
Purification on treated silica, eluent for flash chromatography: Pentane/ Diethylether 90/10
Colorless oil, 105 mg, 43%, Ratio **2k**/**2k'**: 88/12 (24h)
^{19}F NMR (282 MHz, $CDCl_3$) δ -58.43 (s, **2k'**, 0.12), -60.73 (s, **2k**, 0.88).
^{1}H NMR (500 MHz, $CDCl_3$) δ 7.60 – 7.49 (massif, 2H), 7.39 – 7.28 (massif, 3H), 7.23 – 7.16 (massif, 2H), 6.83 – 6.74 (massif, 2H), 4.54 – 4.35 (massif, 2.12H), 4.12 (dd, J_{H-H} = 10.3 Hz, 4.9 Hz, 0.88H), 4.06 (dd, J_{H-H} = 10.3 Hz, 7.9 Hz, 0.88H), 3.45 (m, 0.88H), 3.12 (dd, J_{H-H} = 13.3 Hz, 4.7 Hz, 0.12H), 2.98 (dd, J_{H-H} = 13.3, 8.6 Hz, 0.12H), 1.60 (s, 5.28H), 1.57 (s, 0.72H_6).
^{13}C NMR (126 MHz, $CDCl_3$) δ 173.57, 173.52, 154.08, 154.00, 135.22, 133.37, 129.64, 129.60, 129.35, 128.83, 128.40, 128.08, 127.63, 127.53, 126.75, 121.44 (q, J_{C-F} = 255.7 Hz), 120.73, 120.50, 79.62, 75.97 (q, J_{C-F} = 2.2 Hz), 66.63 (q, J_{C-F} = 3.1 Hz), 64.53, 64.31, 40.06, 27.35, 25.50, 25.44, 25.40, 25.33.
^{77}Se NMR (95 MHz, $CDCl_3$) δ 342.44 (s, **2k**, 0.88), 268.20 (s, **2k'**, 0.12).
HRMS (ESI): Calc. for [M+Na$^+$] $C_{20}H_{20}ClF_3NaO_4Se$: 519.0059, measured: 519.0063

((8*R*,9*S*,13*S*,14*S*)-13-Methyl-3-(2-(phenylselanyl)-3-(trifluoromethoxy)propoxy)-6,7,8,9,11,12,13,14,15,16-decahydro-17*H*-cyclopenta[*a*]phenanthren-17-one (2l)

(8*R*,9*S*,13*S*,14*S*)-13-Methyl-3-(3-(phenylselanyl)-2-(trifluoromethoxy)propoxy)-6,7,8,9,11,12,13,14,15,16-decahydro-17*H*-cyclopenta[*a*]phenanthren-17-one (2l')

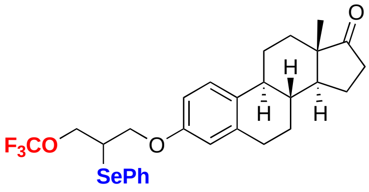

2l

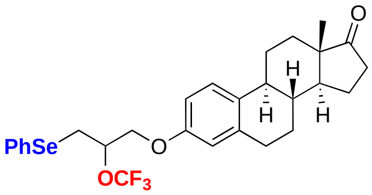

2l'

Procedure A
Purification on treated silica, eluent for flash chromatography: Pentane/Diethylether 70/30
Light-yellow oil, 171 mg, 63%, Ratio **2l**/**2l'**: 78/22, (48h)
^{19}F NMR (282 MHz, $CDCl_3$) δ -58.21 (s, **2l'**, 0.22), -60.47 (s, **2l**, 0.78).
^{1}H NMR (500 MHz, $CDCl_3$) δ 7.53 (m, 1.56H), 7.44 (m, 0.44H), 7.29 – 7.14 (massif, 3H), 7.12 – 7.08 (massif, 1H), 6.62 – 6.56 (massif, 1H), 6.54 – 6.50 (massif, 1H), 4.52 (m, 0.22H), 4.29 (dd, J_{H-H} = 10.1 Hz, 7.5 Hz, 0.78H), 4.22 (m, 1.56H), 4.16 (dd, J_{H-H} = 10.1 Hz, 2.7 Hz, 0.78H), 4.11 (d, J_{H-H} = 4.5 Hz, 0.44H), 3.49 (tt, J_{H-H} = 7.5, 4.5 Hz, 0.78H), 3.25 (dd, J_{H-H} = 13.3, 7.8 Hz, 0.22H), 3.17 (dd, J_{H-H} = 13.2, 5.0 Hz, 0.22H), 2.86 – 2.73 (massif, 2H), 2.47 – 2.36 (massif, 1H), 2.33 – 2.27 (massif, 1H), 2.19 – 1.84 (massif, 5H), 1.59 – 1.28 (massif, 6H), 0.82 (massif, 3H).

^{13}C NMR (126 MHz, $CDCl_3$) δ 221.03, 156.32 (d, $J_{C\text{-}F}$ = 1.7 Hz), 156.18, 138.05, 138.02, 135.09, 133.09, 133.02, 132.93, 129.53, 129.47, 129.00, 128.54, 127.73, 127.62, 126.56, 126.54, 121.66 (q, $J_{C\text{-}F}$ = 255.6 Hz), 121.58 (q, $J_{C\text{-}F}$ = 255.3 Hz), 114.82, 114.80, 112.37 (d, $J_{C\text{-}F}$ = 3.6 Hz), 112.30, 76.72 (q, $J_{C\text{-}F}$ = 2.2 Hz), 67.64, 66.95 (d, $J_{C\text{-}F}$ = 2.3 Hz), 66.70 (q, $J_{C\text{-}F}$ = 3.1 Hz), 50.53, 48.13, 44.11, 41.44, 38.45, 36.00, 31.70, 29.75, 27.85, 26.63, 26.05, 21.72, 13.98.
^{77}Se NMR (95 MHz, $CDCl_3$) δ 342.67 (d, J = 5.5 Hz, **2l**, 0.78), 264.25 (s, **2l'**, 0.22).
HRMS (APCI) : Calc. for [M+H$^+$] $C_{28}H_{32}F_3O_3Se$: 553.1463, measured: 553.1463

[(2-Chlorocyclohexyl)selanyl]benzene (3a)

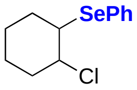

Colorless oil, 107 mg, 79%
^{1}H NMR (300 MHz, $CDCl_3$) δ 7.59 (m, 2H), 7.35 – 7.23 (massif, 3H), 4.15 (ddd, $J_{H\text{-}H}$ = 6.7, 6.7, 3.8 Hz, 1H), 3.47 (ddd, $J_{H\text{-}H}$ = 6.7, 6.7, 3.8 Hz, 1H), 2.46 – 2.21 (massif, 2H), 1.86 – 1.73 (massif, 4H), 1.36 – 1.51 (massif, 2H).
The results are in accordance with literature data [4]

2-(Phenylselanyl)cyclohexyl 2,2,2-trifluoroacetate (4a)

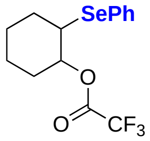

Procedure E
Colorless oil, 56% (NMR yield with PhCF3 as an internal standard)
^{19}F NMR (282 MHz, $CDCl_3$) δ -75.11 (s).
^{1}H NMR (300 MHz, $CDCl_3$) δ 7.59 (m, 2H), 7.34 – 7.27 (massif, 3H), 5.00 (ddd, $J_{H\text{-}H}$ = 9.1, 9.1, 4.2 Hz, 1H), 3.22 (ddd, $J_{H\text{-}H}$ = 9.1, 9.1, 4.2 Hz, 1H), 2.27 – 2.10 (massif, 2H), 1.69 – 1.54 (massif, 4H), 1.43 – 1.35 (massif, 2H).
The results are in accordance with the literature [3]

3-(Trifluoromethoxy)propyl 2-(4-chlorophenoxy)-2-methylpropanoate (5k)

2-(Trifluoromethoxy)propyl 2-(4-chlorophenoxy)-2-methylpropanoate (5k')

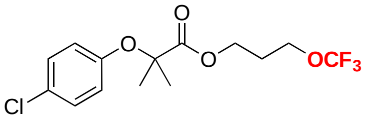

5k

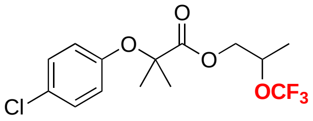

5k'

Procedure B
Eluent for flash chromatography: Cyclohexane, then Pentane/Diethylether 95/5
Colorless oil, 70 mg, 51%, Ratio **5k**/**5k'**: 89/11
^{19}F NMR (282 MHz, $CDCl_3$) δ -58.49 (s, **5k'**, 0.11), -61.03 (s, **5k**, 0.89).
^{1}H NMR (500 MHz, $CDCl_3$) δ 7.23 – 7.16 (massif, 2H), 6.82 – 6.73 (massif, 2H), 4.53 (m, 0.11H), 4.26 (t, $J_{H\text{-}H}$ = 6.1 Hz, 1.78H), 4.20 (m, 0.22H), 3.91 (t, $J_{H\text{-}H}$ = 6.1 Hz, 1.78H), 2.00 (quint, $J_{H\text{-}H}$ = 6.1 Hz, 1.78H), 1.63 - 1.54 (massif, 6H), 1.32 (d, $J_{H\text{-}H}$ = 6.5 Hz, 0.33H).
^{13}C NMR (126 MHz, $CDCl_3$) δ 173.97, 173.71, 154.14, 154.03, 129.33, 129.29, 127.45, 126.67 (q, $J_{C\text{-}F}$ = 253.5 Hz), 121.64 (q, $J_{C\text{-}F}$ = 254.4 Hz), 120.97, 120.31, 79.68, 79.57, 73.10 (q, $J_{C\text{-}F}$ = 2.6 Hz), 66.73, 63.70 (q, $J_{C\text{-}F}$ = 3.3 Hz), 61.40, 28.00, 25.43, 17.37.
Calc. for [M+Na$^+$] [$C_{14}H_{16}ClF_3NaO_4$]: 363.0581, measured: 363.0581

(8*R*,9*S*,13*S*,14*S*)-13-Methyl-3-(3-(trifluoromethoxy)propoxy)-6,7,8,9,11,12,13,14,15,16-decahydro-17*H*-cyclopenta[*a*]phenanthren-17-one (5l)

(8*R*,9*S*,13*S*,14*S*)-13-Methyl-3-(2-(trifluoromethoxy)propoxy)-6,7,8,9,11,12,13,14,15,16-decahydro-17*H*-cyclopenta[*a*]phenanthren-17-one (5l')

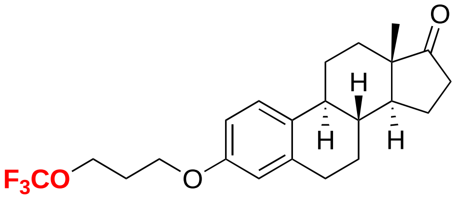

5l

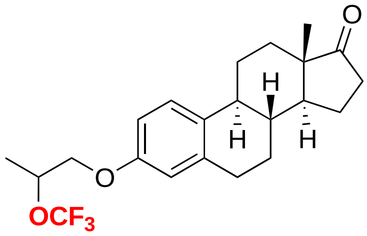

5l'

Procedure B
Eluent for flash chromatography: Cyclohexane, then Pentane/Diethylether 90/10
Slightly yellow oil, 113 mg, 52%, Ratio **5l**/**5l'**: 74/26
^{19}F NMR (471 MHz, $CDCl_3$) δ -58.24 (s, **5l'**, 0.26), -60.71 (s, **5l**, 0.74).
^{1}H NMR (500 MHz, $CDCl_3$) δ 7.23 – 7.19 (massif, 1H), 6.74 – 6.70 (massif, 1H), 6.67 - 6.64 (massif, 1H), 4.68 – 4.62 (m, 0.26H), 4.18 (t, $J_{H\text{-}H}$ = 6.2 Hz, 1.48H), 4.08 – 4.01 (massif, 1.74H), 3.95 (m, 0.26H), 2.93 – 2.86 (massif, 2H), 2.57 – 2.47 (massif, 1H), 2.43 – 2.38 (massif, 1H), 2.32 – 2.23 (massif, 1H), 2.20 – 1.93 (massif, 5.48H), 1.69 – 1.43 (massif, 6.78H), 0.93-0.84 (massif, 3H).
^{13}C NMR (126 MHz, $CDCl_3$) δ 221.01, 220.98, 156.75, 156.41, 138.04, 137.95, 132.89, 132.50, 126.54, 126.50, 121.79 (q, $J_{C\text{-}F}$ = 254.5 Hz), 121.76 (q, $J_{C\text{-}F}$ = 250.5 Hz), 114.81 (d, $J_{C\text{-}F}$ = 2.3 Hz), 114.64, 112.24, 112.22, 73.82 (q, $J_{C\text{-}F}$ = 2.4 Hz), 70.11, 64.24 (q, $J_{C\text{-}F}$ = 3.3 Hz), 63.40, 50.50, 48.10, 44.08, 38.45, 38.42, 35.96, 31.68, 29.74, 29.71, 28.91, 26.63, 26.60, 26.02, 21.68, 17.77, 13.94.
HRMS (ESI): Calc. for $[M+H^+]$ $[C_{22}H_{28}F_3O_3]$: 397.1985, measured: 397.1987

References

[1] C. Guo, X. Han, Y. Feng, Z. Liu, Y. Li, H. Liu, L. Zhang, Y. Dong, X. Li, *J. Org. Chem.* **2022**, *87*, 9232-9241.

[2] T. Fujihara, K. Semba, J. Terao, Y. Tsuji, *Angew. Chem. Int. Ed.* **2010**, *49*, 1472-1476.

[3] J. M. White, J. B. Lambert, M. Spiniello, S. A. Jones, R. W. Gable, *Chem. Eur. J.* **2002**, *8 12*, 2799-2811.

[4] S. E. Denmark, W. R. Collins, M. D. Cullen, *J. Am. Chem. Soc.* **2009**, *131*, 3490-3492.

NMR Spectra

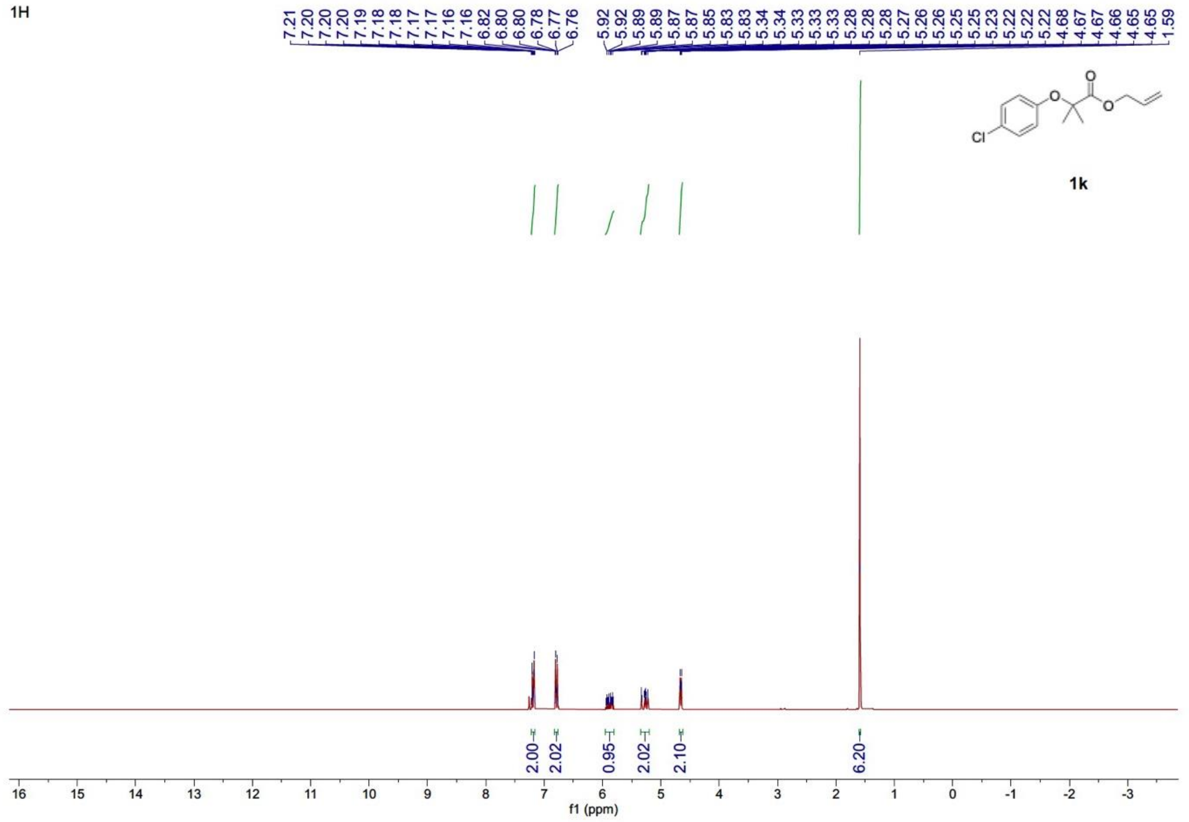
1H
7.21 7.20 7.20 7.19 7.18 7.17 7.17 7.16 7.16 6.82 6.80 6.80 6.78 6.77 6.76
5.92 5.92 5.89 5.89 5.87 5.87 5.85 5.83 5.83 5.34 5.33 5.33 5.33 5.28 5.28 5.28 5.27 5.26 5.26 5.25 5.25 5.23 5.22 5.22 5.22 4.68 4.67 4.67 4.66 4.65 4.65 1.59
Cl
O
1k
2.00 2.02 0.95 2.02 2.10 6.20
f1 (ppm)

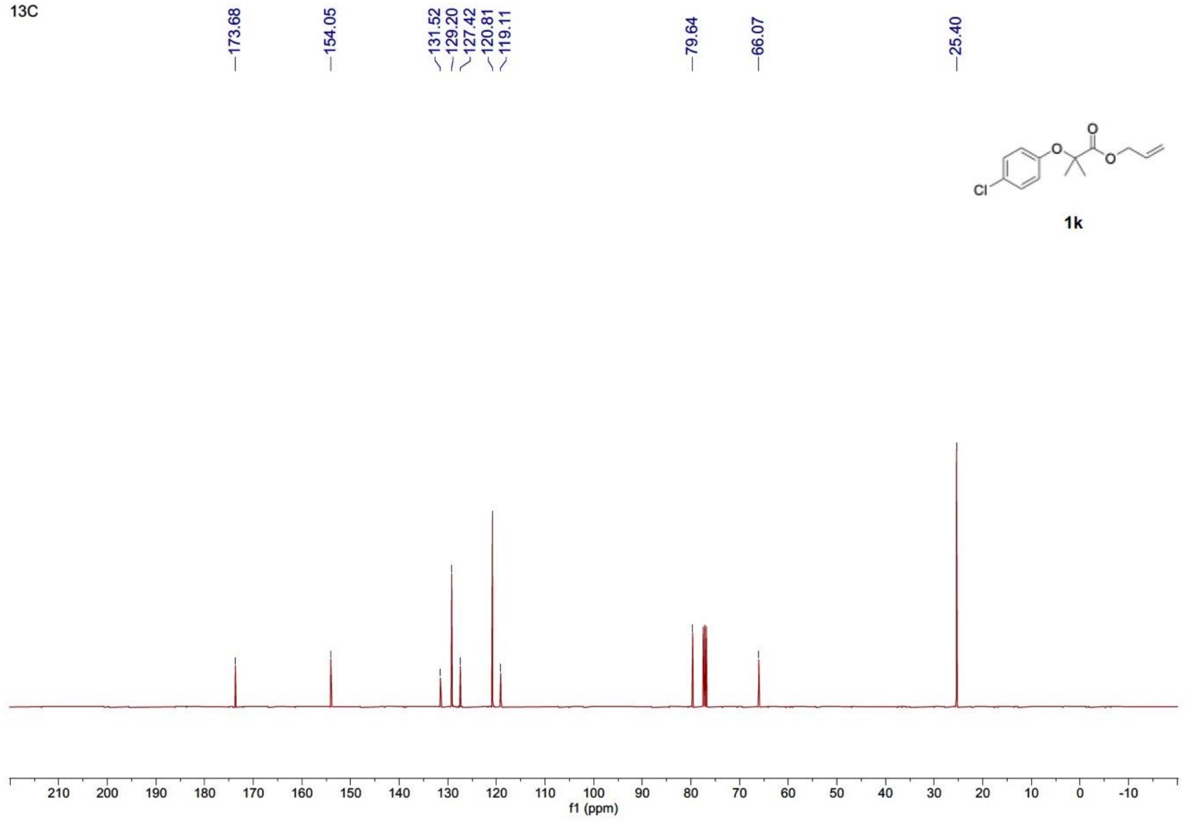
13C
173.68
154.05
131.52 129.20 127.42 120.81 119.11
79.64
66.07
25.40
Cl
O
1k
f1 (ppm)

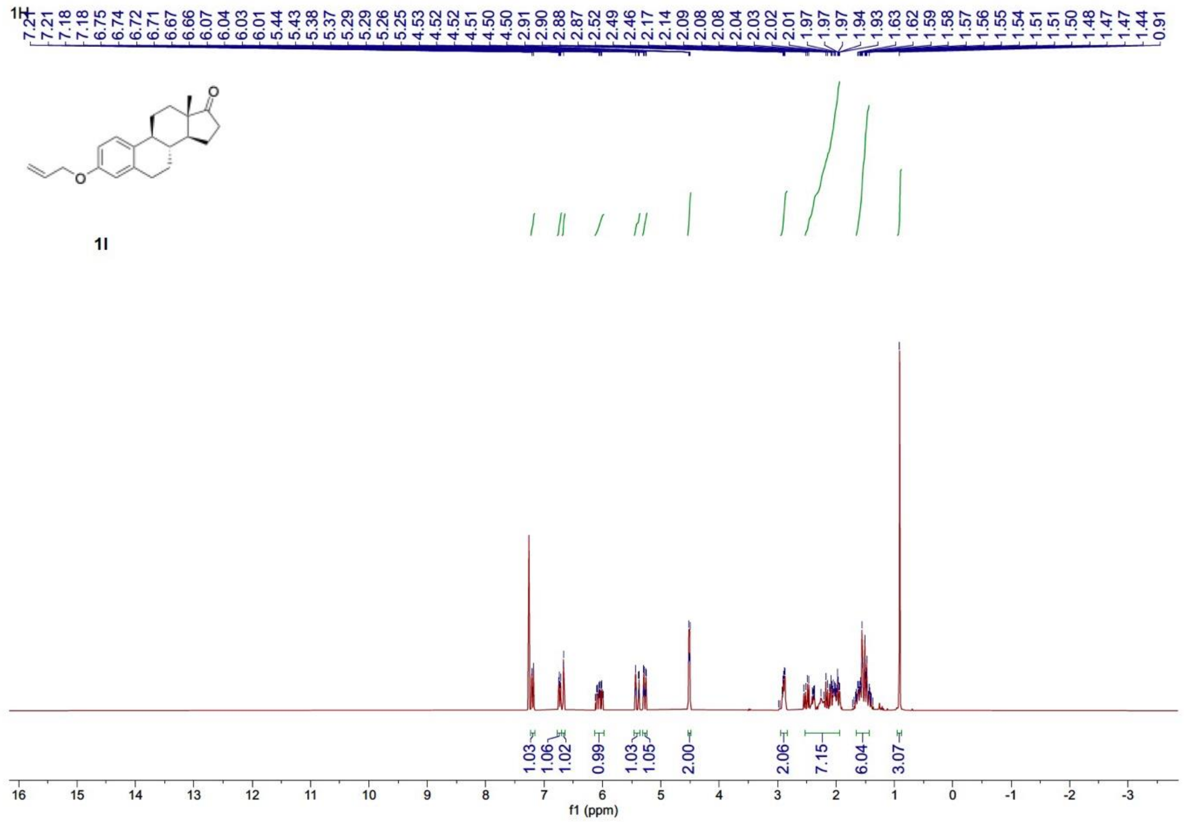
1H
1l
f1 (ppm)

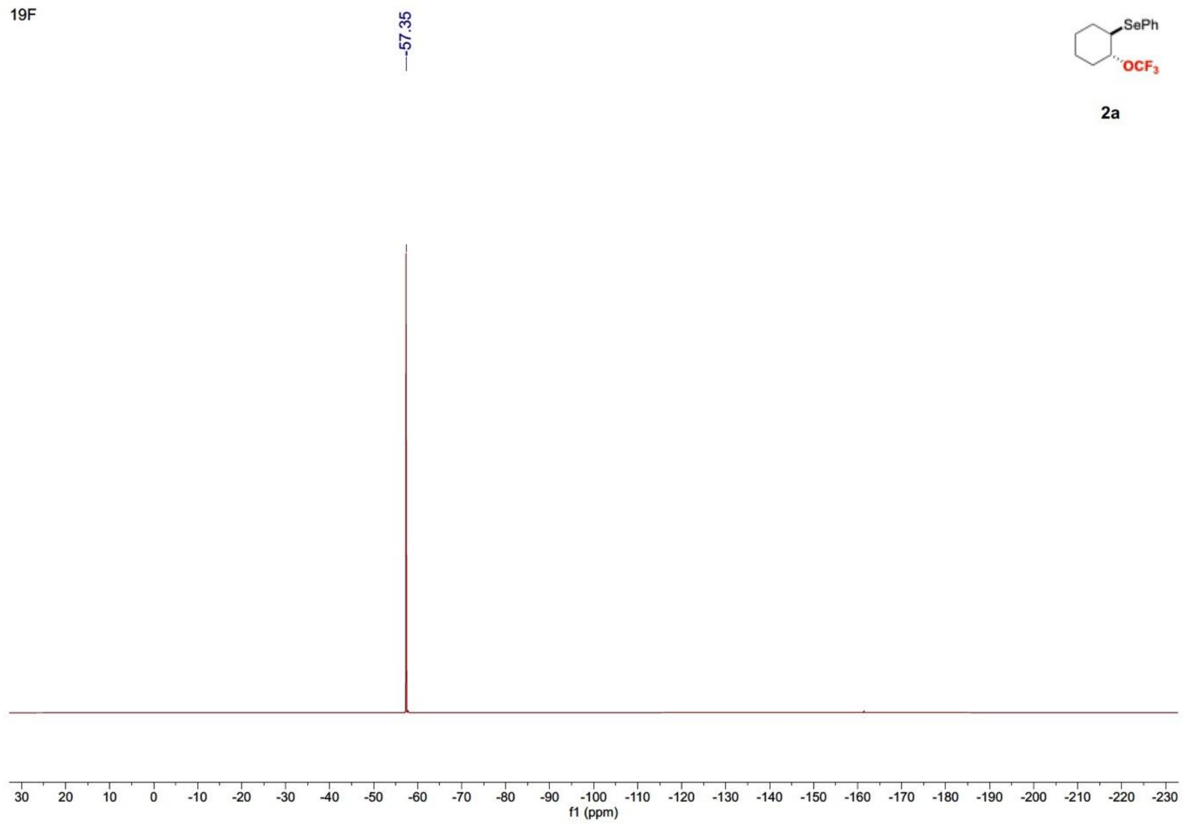
19F
-57.35
SePh
OCF3
2a
f1 (ppm)

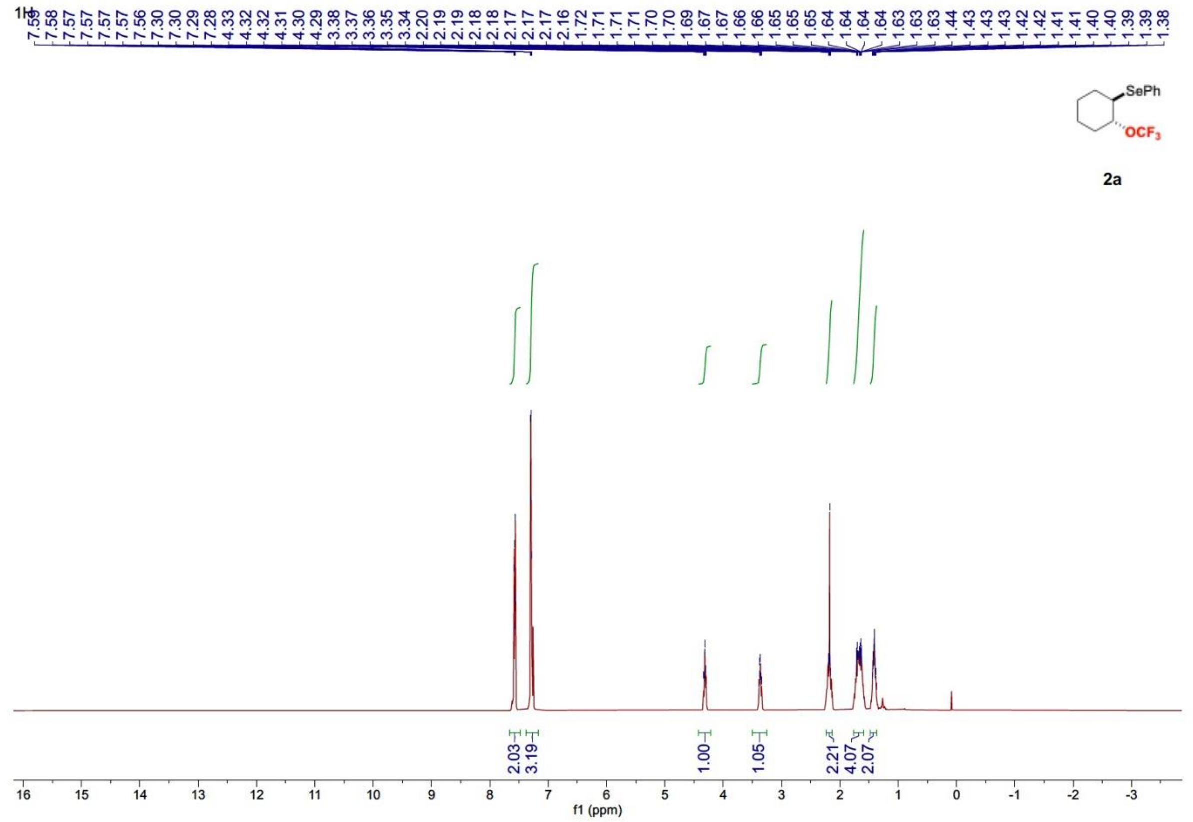
1H
7.59
7.58
7.57
7.57
7.57
7.56
7.30
7.30
7.29
7.28
4.33
4.32
4.31
4.30
4.29
3.38
3.37
3.36
3.35
3.34
2.20
2.19
2.19
2.18
2.18
2.17
2.17
2.17
2.16
1.72
1.71
1.71
1.71
1.70
1.69
1.67
1.67
1.66
1.65
1.65
1.65
1.64
1.64
1.64
1.64
1.63
1.63
1.44
1.43
1.43
1.43
1.42
1.42
1.41
1.41
1.40
1.39
1.39
1.38
SePh
OCF3
2a
2.03
3.19
1.00
1.05
2.21
4.07
2.07
f1 (ppm)

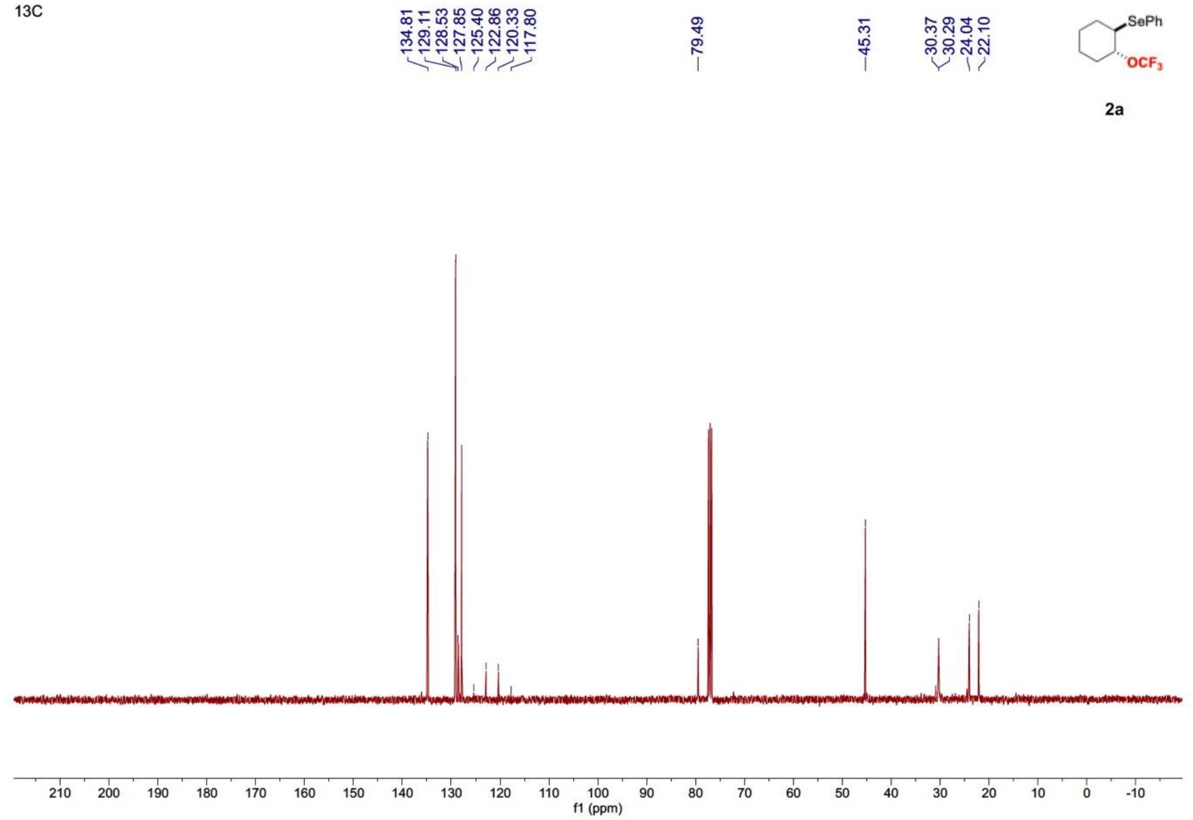
13C
134.81
129.11
128.53
127.85
125.40
122.86
120.33
117.80
79.49
45.31
30.37
30.29
24.04
22.10
SePh
OCF3
2a
f1 (ppm)

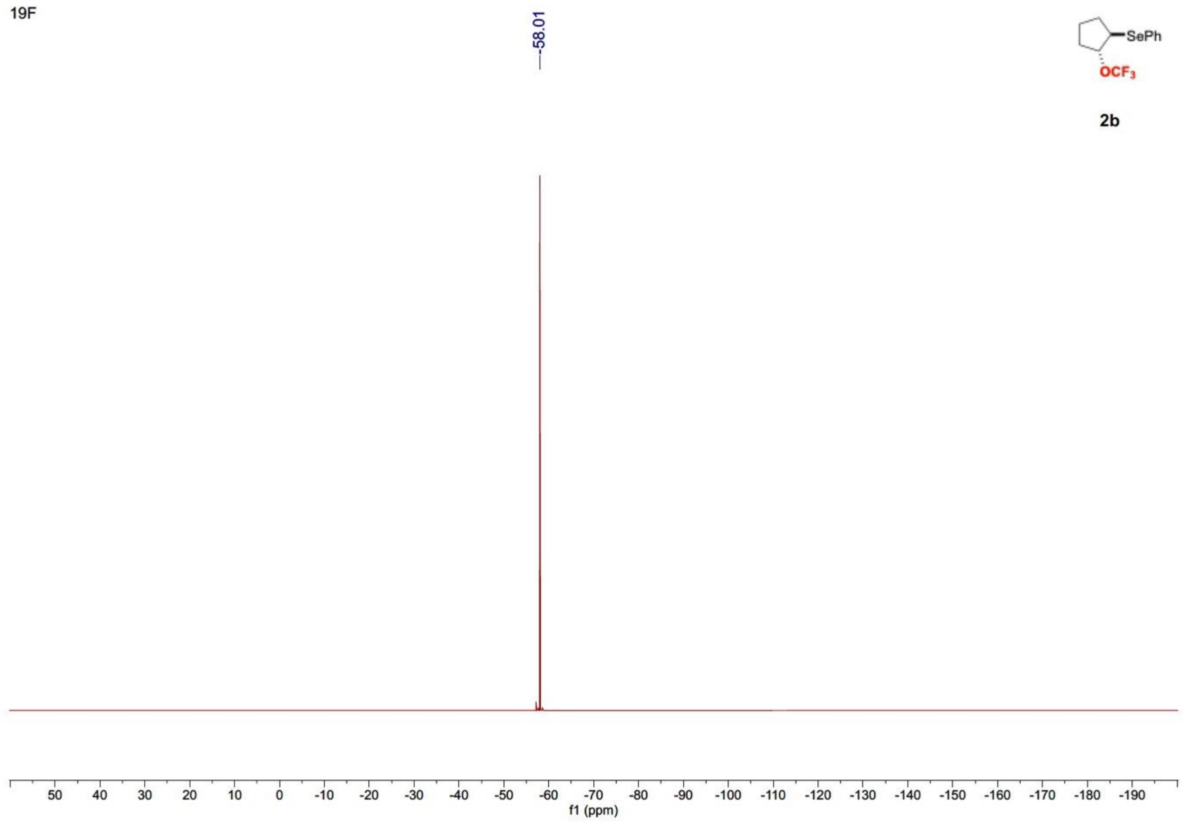
19F
-58.01
SePh
OCF3
2b
50 40 30 20 10 0 -10 -20 -30 -40 -50 -60 -70 -80 -90 -100 -110 -120 -130 -140 -150 -160 -170 -180 -190
f1 (ppm)

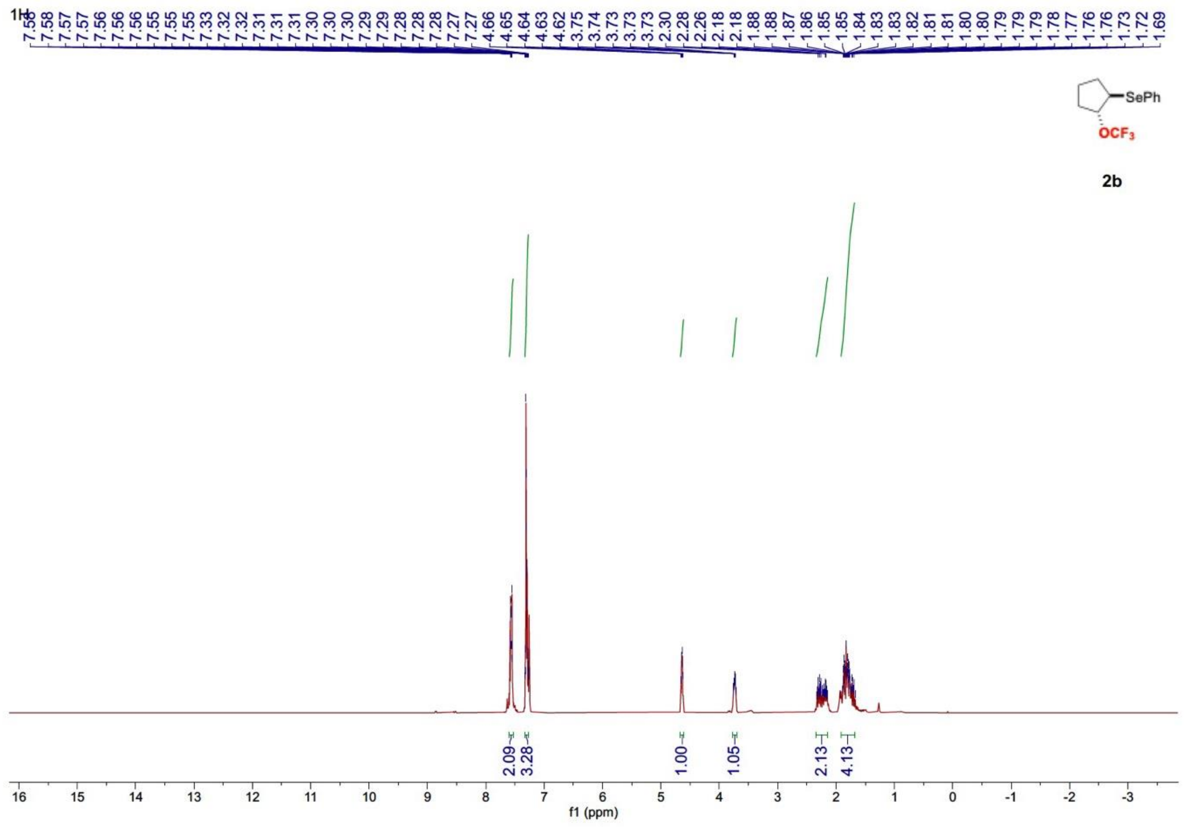
1H
7.58 7.58 7.57 7.56 7.56 7.55 7.55 7.33 7.32 7.32 7.31 7.31 7.31 7.30 7.30 7.29 7.29 7.28 7.28 7.28 7.27 4.66 4.65 4.64 4.63 4.62 3.75 3.74 3.73 3.73 3.73 2.30 2.28 2.26 2.18 2.18 1.88 1.88 1.87 1.86 1.85 1.85 1.84 1.83 1.83 1.82 1.81 1.81 1.80 1.80 1.79 1.79 1.78 1.77 1.76 1.76 1.73 1.72 1.69
SePh
OCF3
2b
2.09
3.28
1.00
1.05
2.13
4.13
16 15 14 13 12 11 10 9 8 7 6 5 4 3 2 1 0 -1 -2 -3
f1 (ppm)

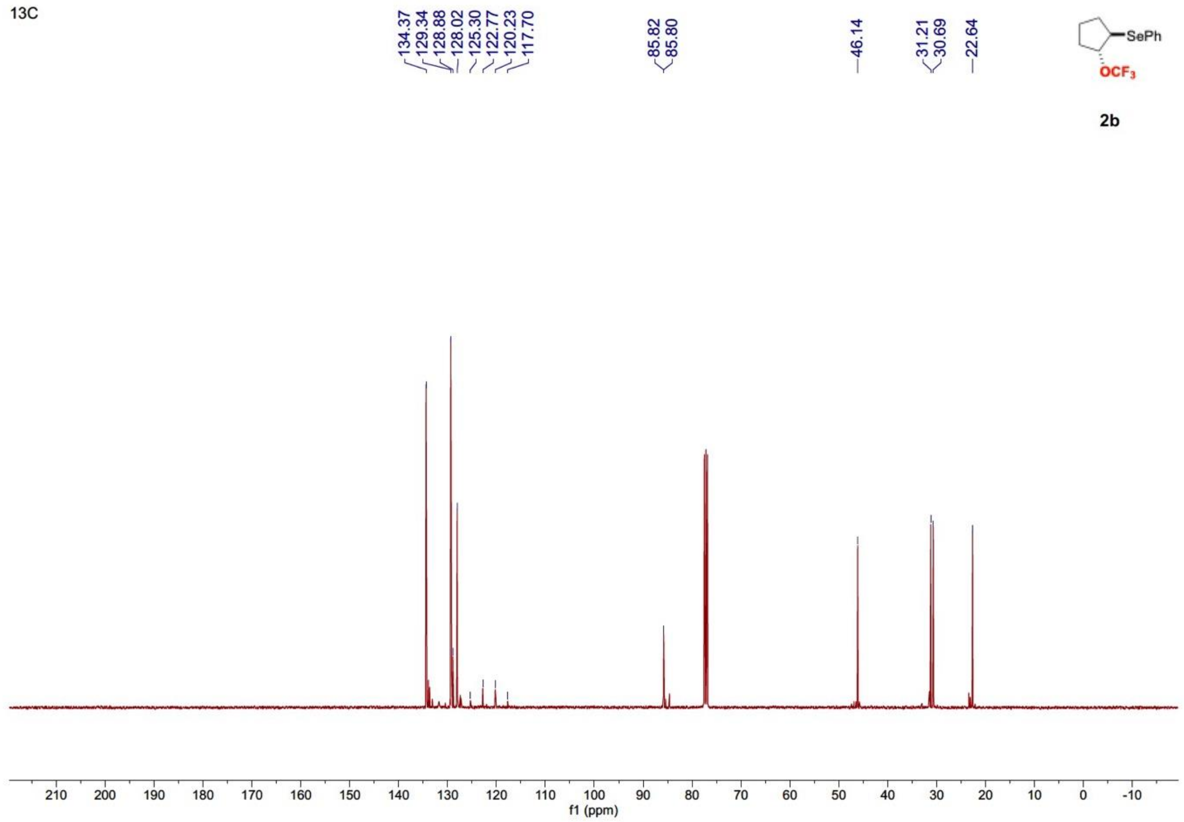
13C
134.37
129.34
128.88
128.02
125.30
122.77
120.23
117.70
85.82
85.80
46.14
31.21
30.69
22.64
SePh
OCF3
2b
210 200 190 180 170 160 150 140 130 120 110 100 90 80 70 60 50 40 30 20 10 0 -10
f1 (ppm)

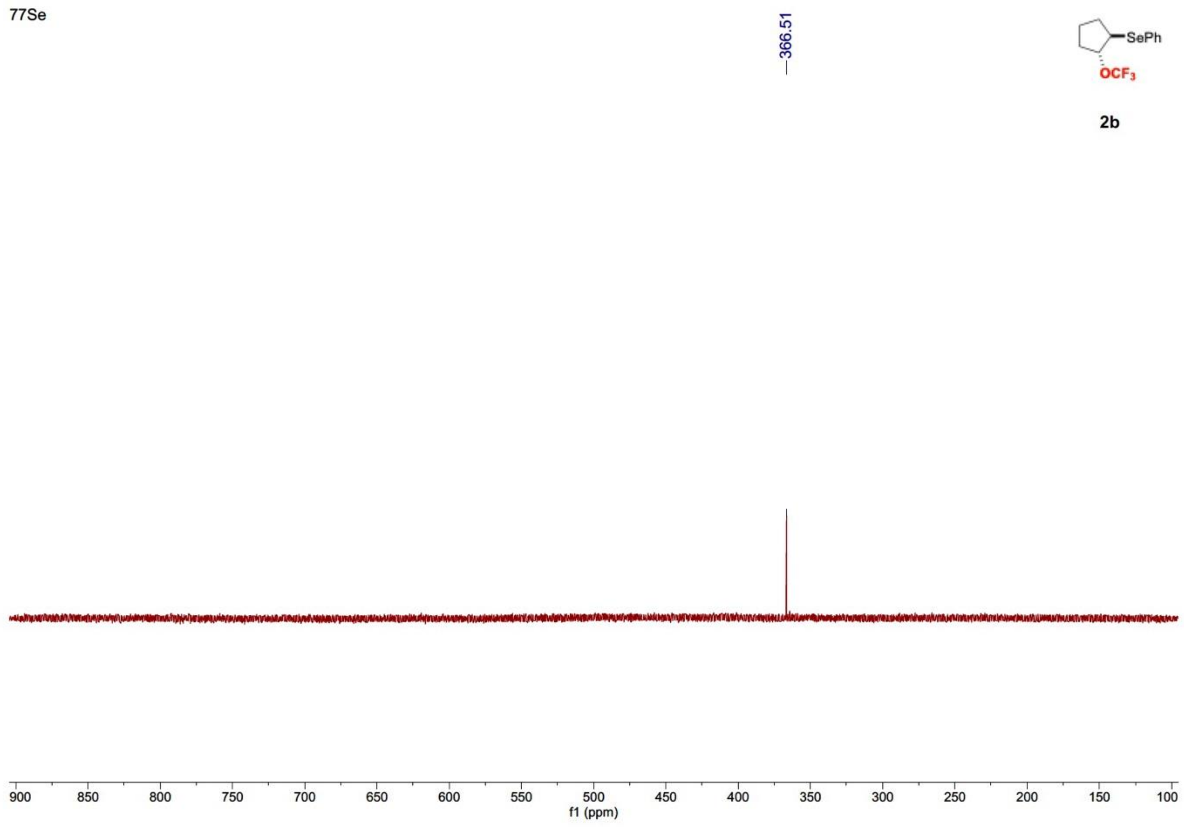
77Se
366.51
SePh
OCF3
2b
900 850 800 750 700 650 600 550 500 450 400 350 300 250 200 150 100
f1 (ppm)

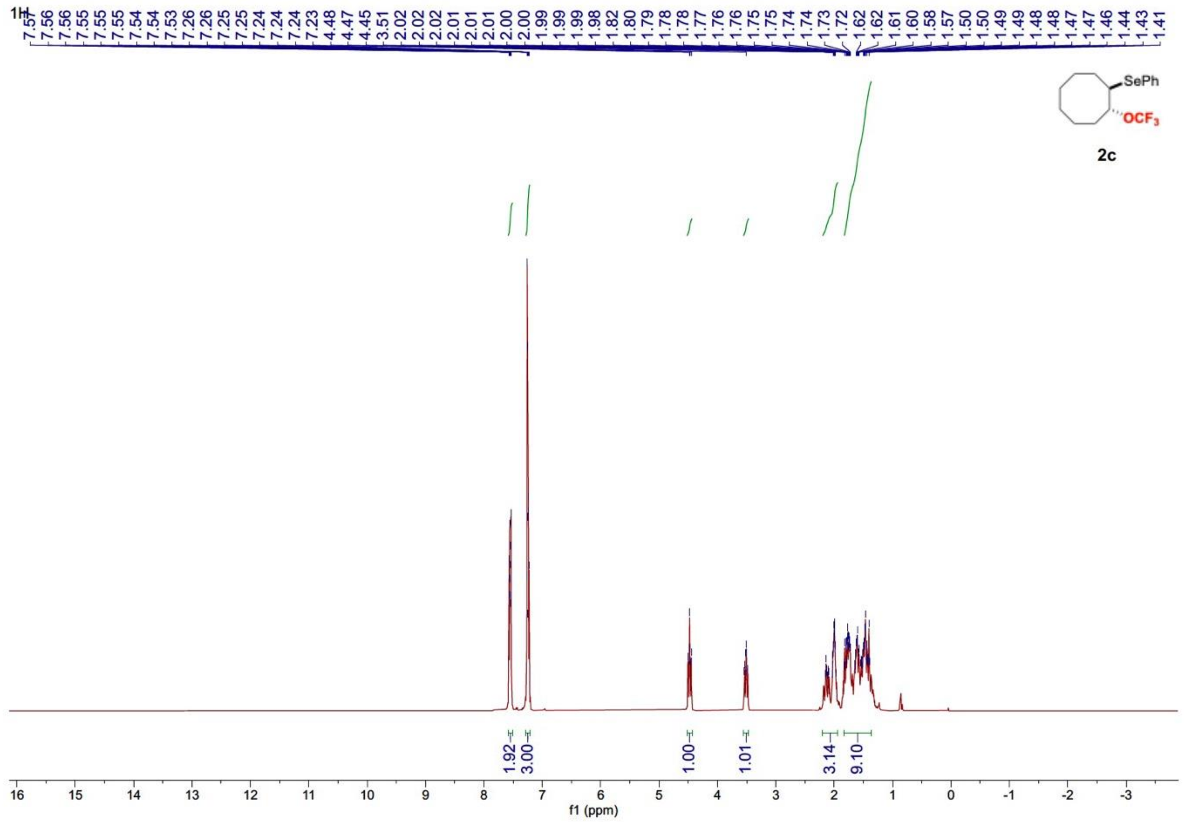
1H
7.57 7.56 7.56 7.55 7.55 7.55 7.54 7.54 7.53 7.26 7.26 7.25 7.25 7.24 7.24 7.24 7.23 4.48 4.47 4.45 3.51 2.02 2.02 2.02 2.01 2.01 2.01 2.00 2.00 1.99 1.99 1.99 1.98 1.82 1.80 1.79 1.78 1.78 1.77 1.76 1.76 1.75 1.75 1.74 1.74 1.73 1.72 1.62 1.62 1.61 1.60 1.58 1.57 1.50 1.49 1.49 1.48 1.48 1.47 1.47 1.46 1.44 1.43 1.41
SePh
OCF3
2c
1.92
3.00
1.00
1.01
3.14
9.10
f1 (ppm)

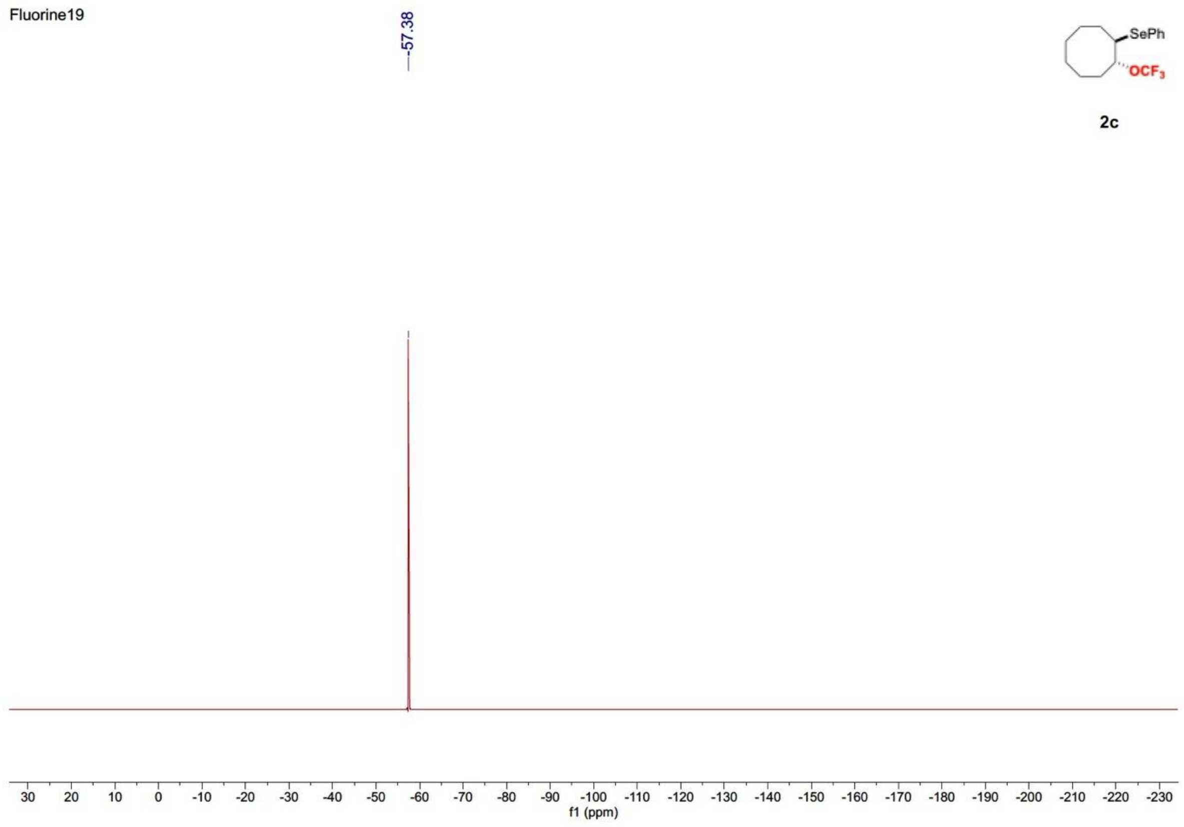
Fluorine19
-57.38
SePh
OCF3
2c
f1 (ppm)

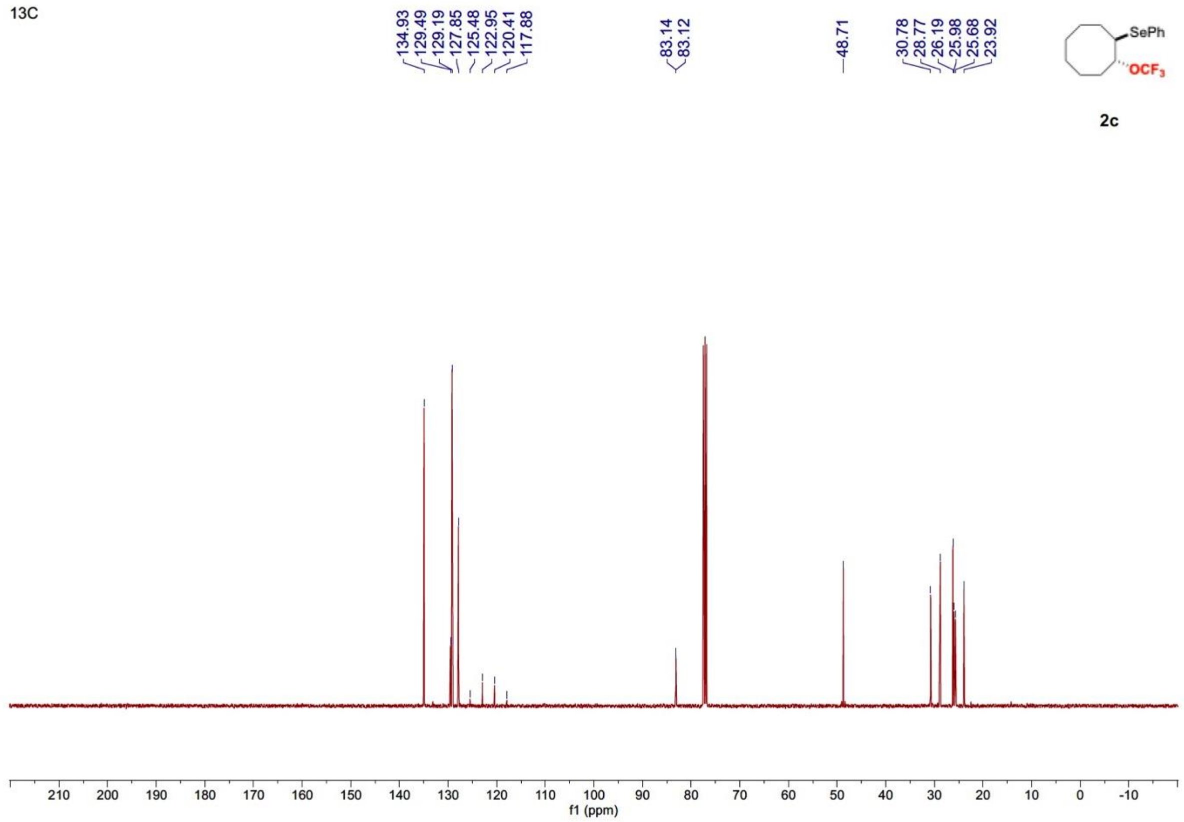
13C
134.93
129.49
129.19
127.85
125.48
122.95
120.41
117.88
83.14
83.12
48.71
30.78
28.77
26.19
25.98
25.68
23.92
SePh
OCF3
2c
210 200 190 180 170 160 150 140 130 120 110 100 90 80 70 60 50 40 30 20 10 0 -10
f1 (ppm)

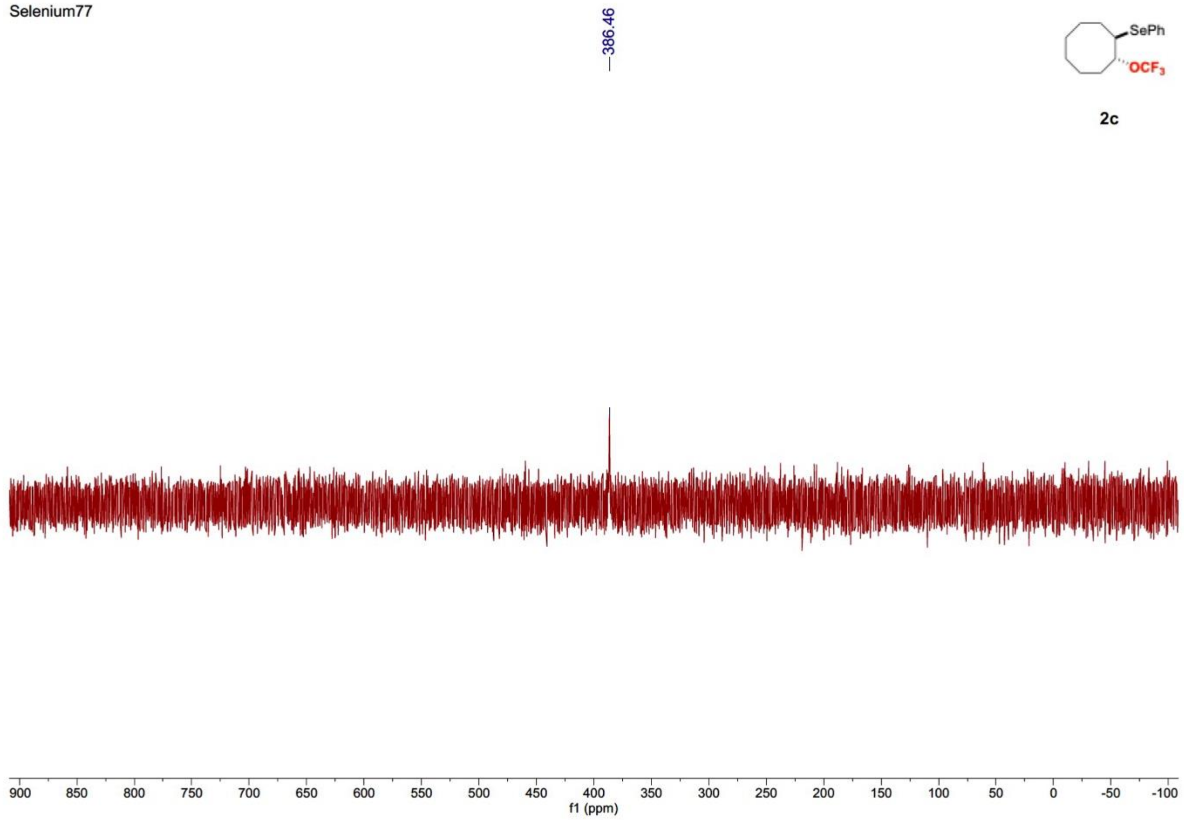
Selenium77
386.46
SePh
OCF3
2c
900 850 800 750 700 650 600 550 500 450 400 350 300 250 200 150 100 50 0 -50 -100
f1 (ppm)

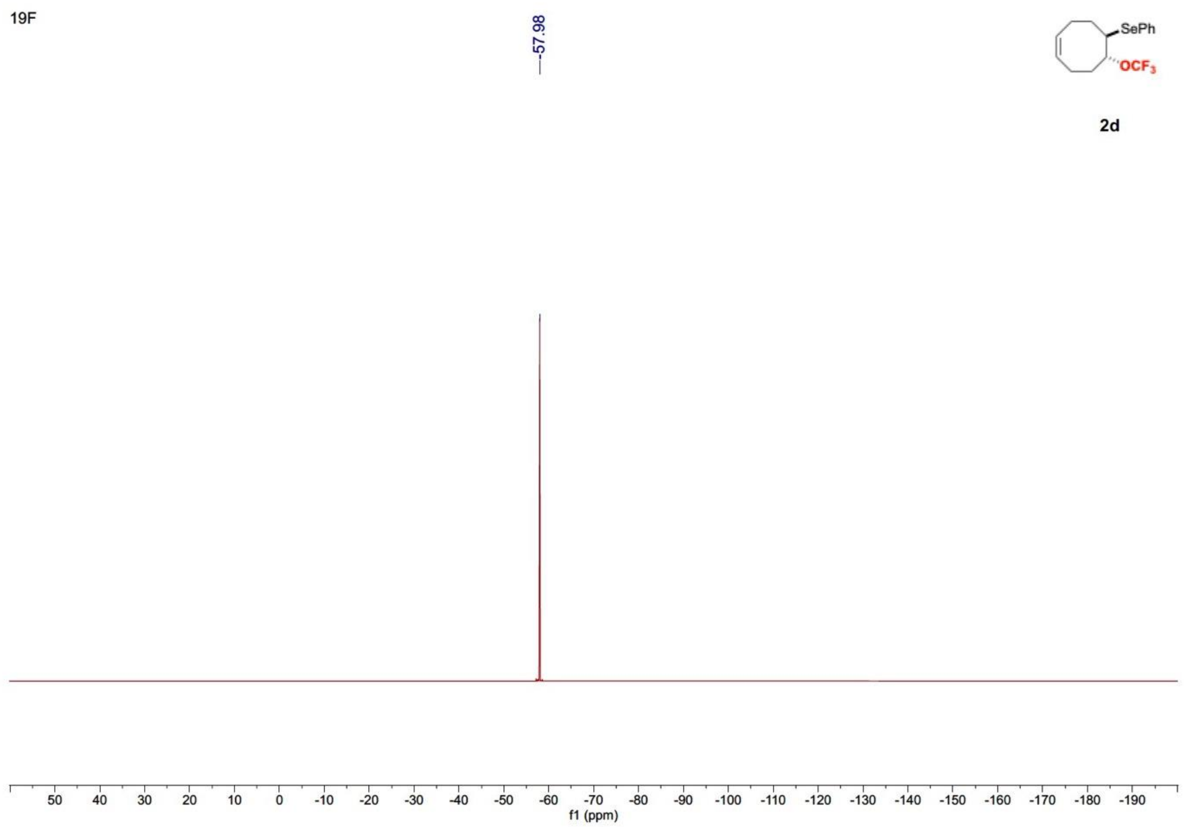
19F
-57.98
SePh
OCF3
2d
f1 (ppm)

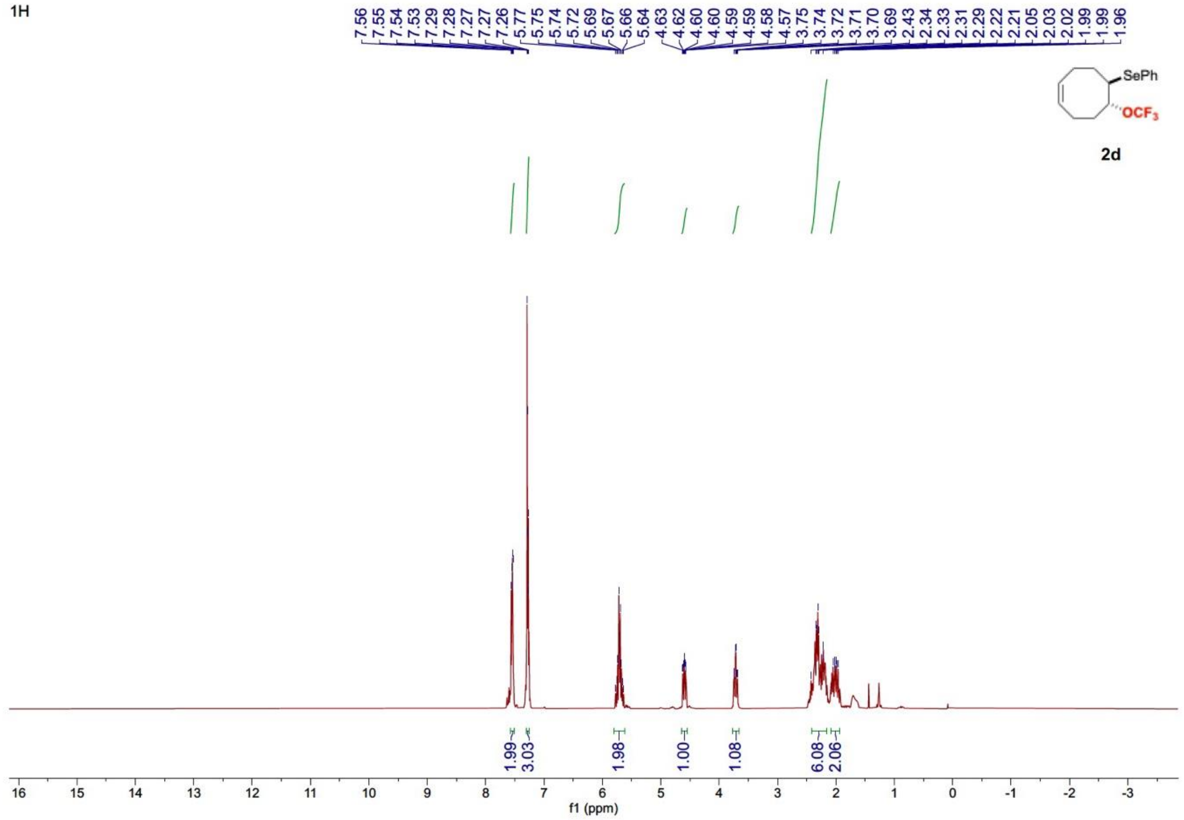
1H
7.56 7.55 7.54 7.53 7.29 7.28 7.27 7.27 7.26 5.77 5.75 5.74 5.72 5.69 5.67 5.66 5.64 4.63 4.62 4.60 4.60 4.59 4.58 4.57 3.75 3.74 3.72 3.71 3.70 3.69 2.43 2.34 2.33 2.31 2.29 2.22 2.21 2.05 2.03 2.02 1.99 1.96
SePh
OCF3
2d
1.99 3.03 1.98 1.00 1.08 6.08 2.06
f1 (ppm)

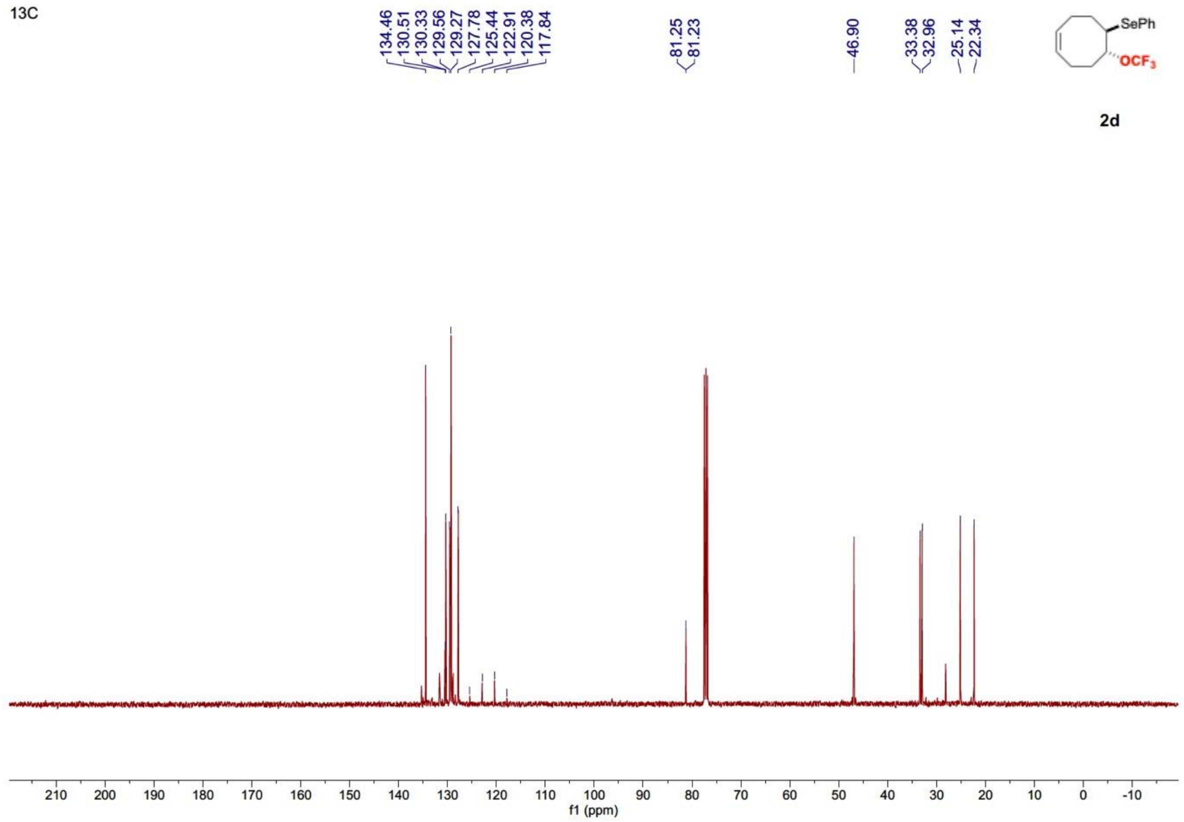
13C
134.46
130.51
130.33
129.56
129.27
127.78
125.44
122.91
120.38
117.84
81.25
81.23
46.90
33.38
32.96
25.14
22.34
SePh
OCF3
210 200 190 180 170 160 150 140 130 120 110 100 90 80 70 60 50 40 30 20 10 0 -10
f1 (ppm)

2d

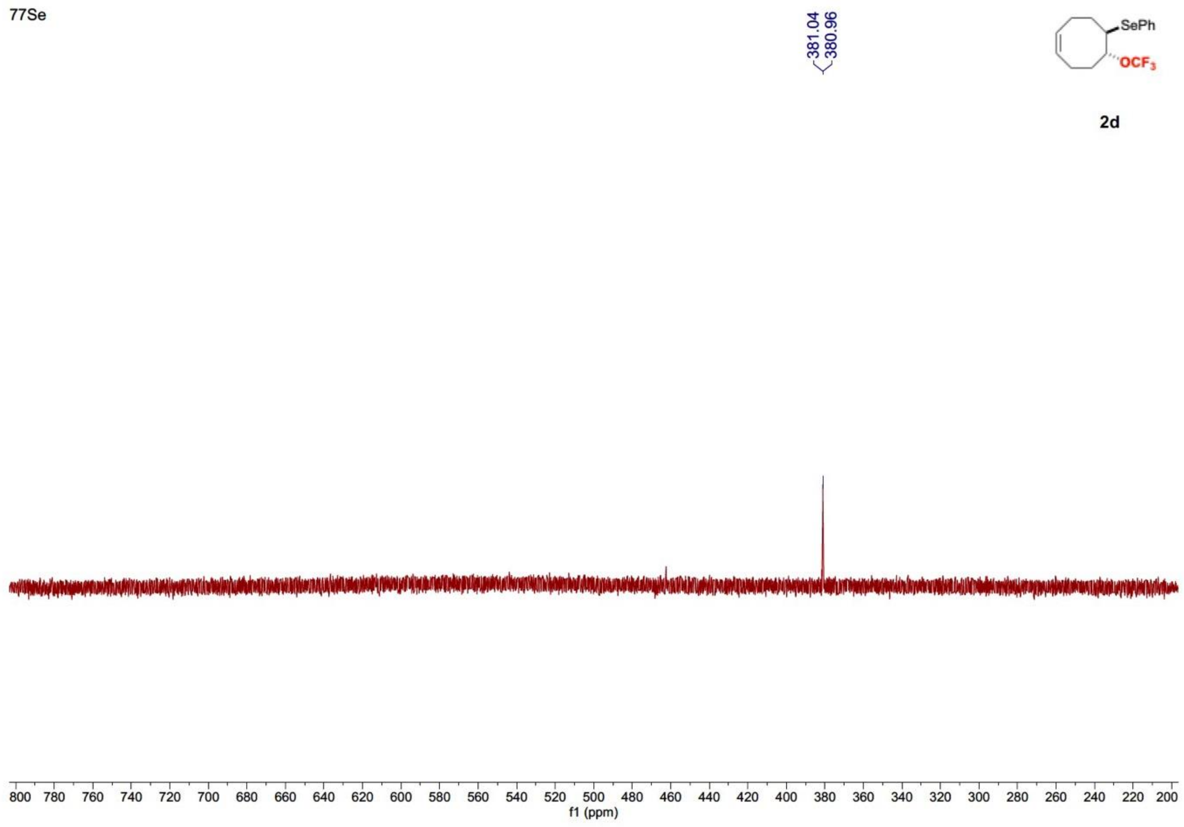
77Se
381.04
380.96
SePh
OCF3
800 780 760 740 720 700 680 660 640 620 600 580 560 540 520 500 480 460 440 420 400 380 360 340 320 300 280 260 240 220 200
f1 (ppm)

2d

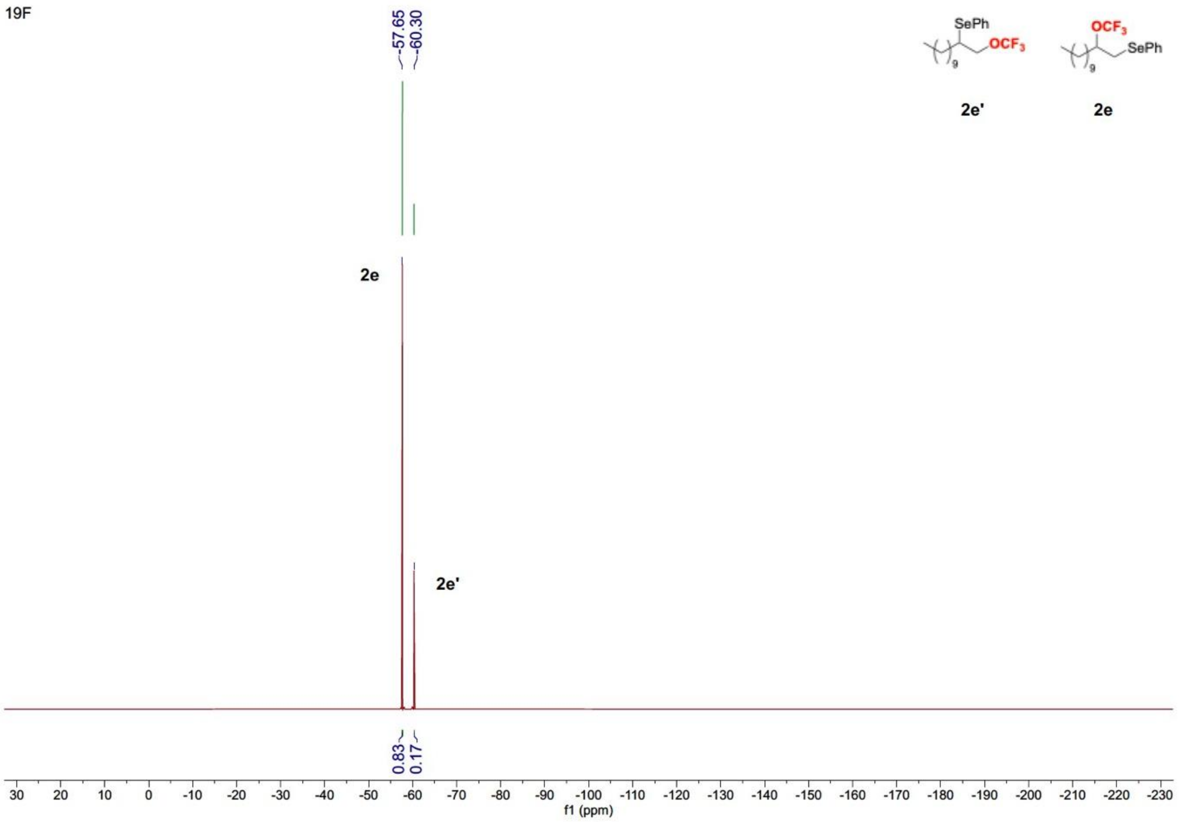
19F
-57.65
-60.30
SePh
OCF3
OCF3
SePh
2e'
2e
2e
2e'
0.83
0.17
f1 (ppm)

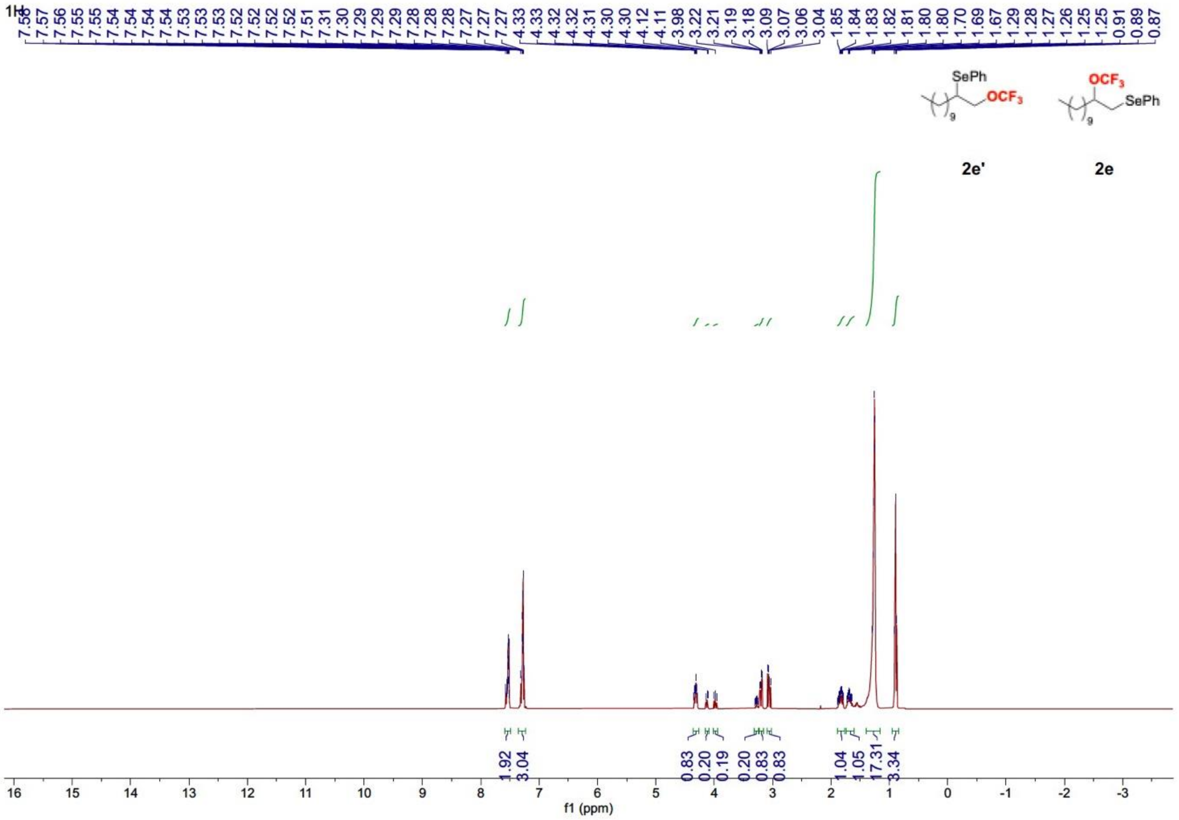
1H
SePh
OCF3
OCF3
SePh
2e'
2e
1.92
3.04
0.83
0.20
0.19
0.20
0.83
0.83
1.04
1.05
17.31
3.34
f1 (ppm)

13C

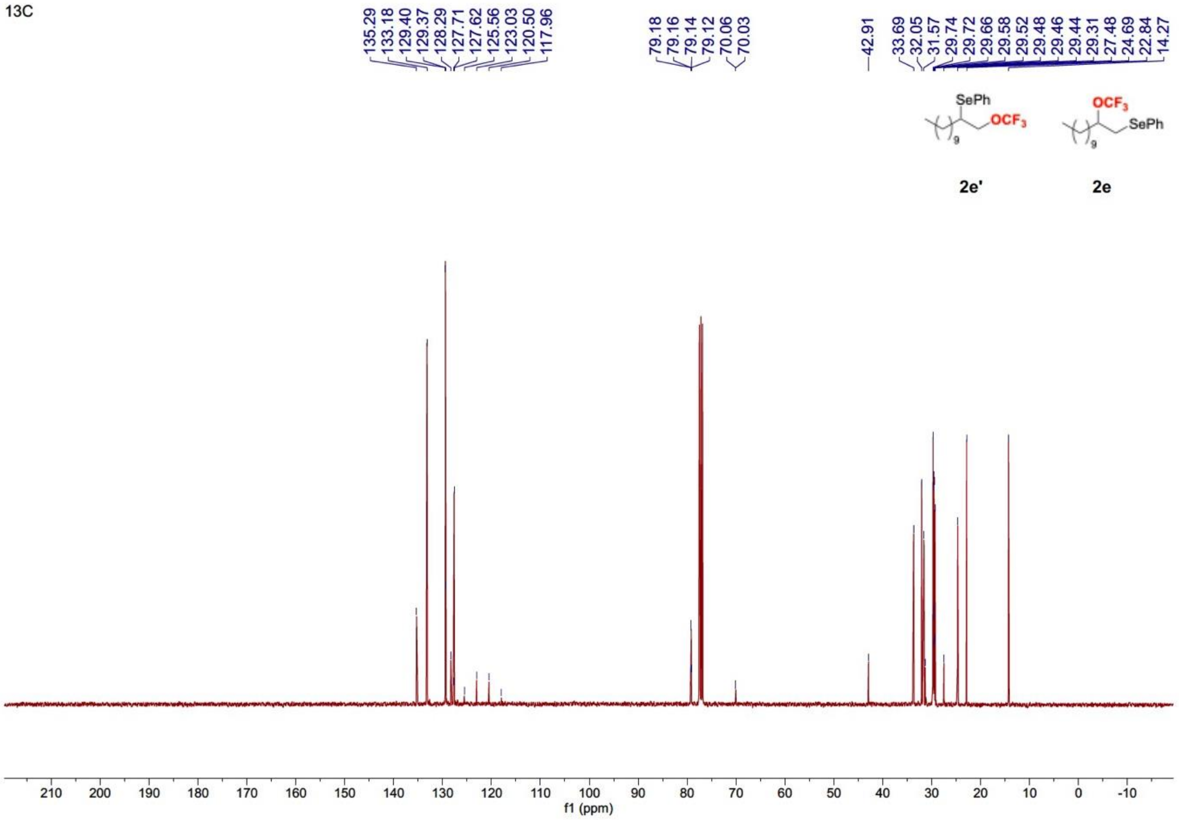
135.29
133.18
129.40
129.37
128.29
127.71
127.62
125.56
123.03
120.50
117.96
79.18
79.16
79.14
79.12
70.06
70.03
42.91
33.69
32.05
31.57
29.74
29.72
29.66
29.58
29.52
29.48
29.46
29.44
29.31
27.48
24.69
22.84
14.27
SePh
OCF3
9
2e'
OCF3
SePh
9
2e
f1 (ppm)

77Se

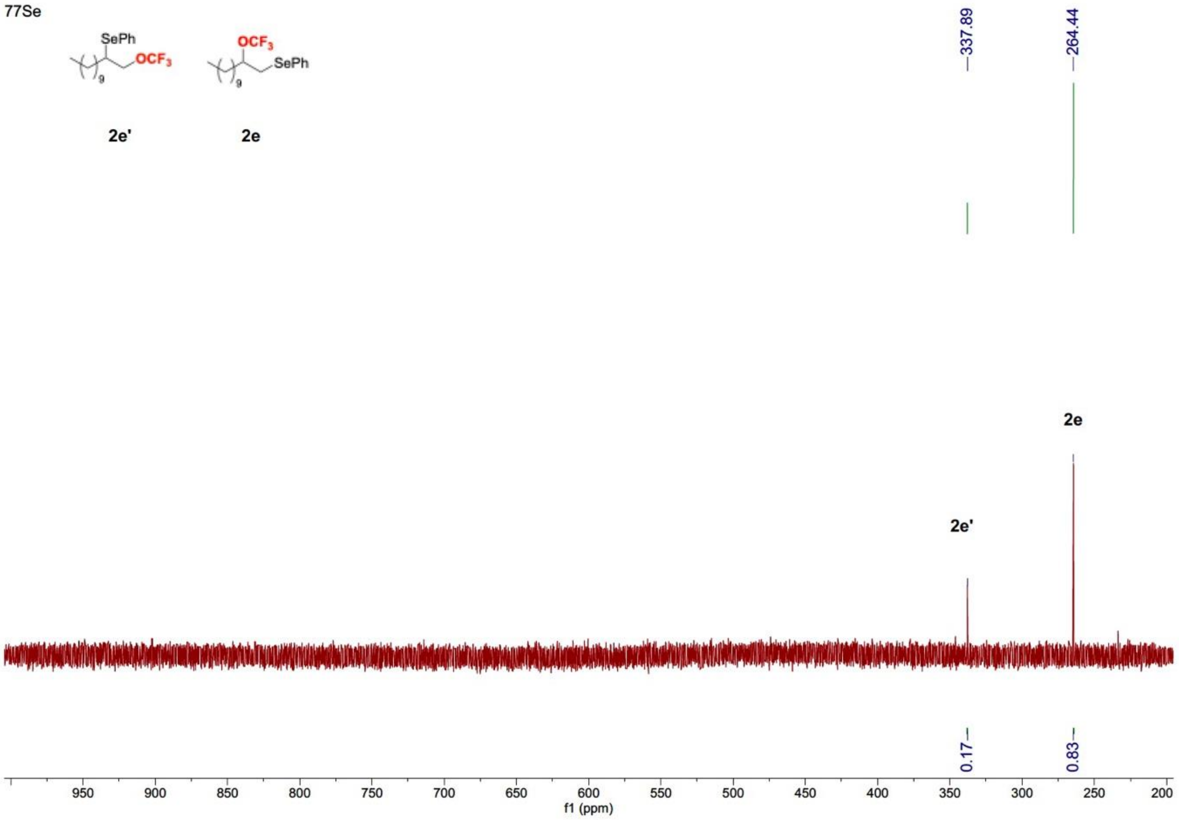
SePh
OCF3
9
2e'
OCF3
SePh
9
2e
337.89
264.44
2e
2e'
0.17
0.83
f1 (ppm)

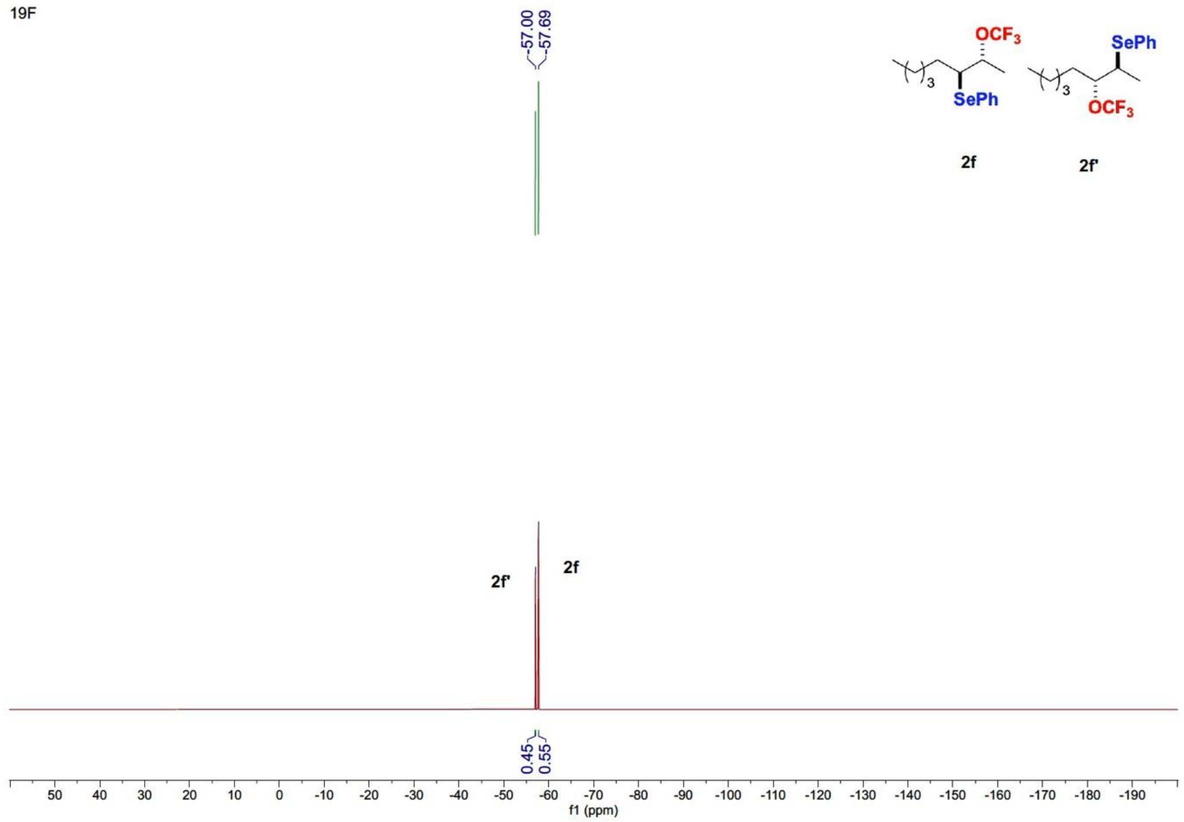
19F
-57.00
-57.69
OCF3
SePh
2f
2f'
0.45
0.55
50 40 30 20 10 0 -10 -20 -30 -40 -50 -60 -70 -80 -90 -100 -110 -120 -130 -140 -150 -160 -170 -180 -190
f1 (ppm)

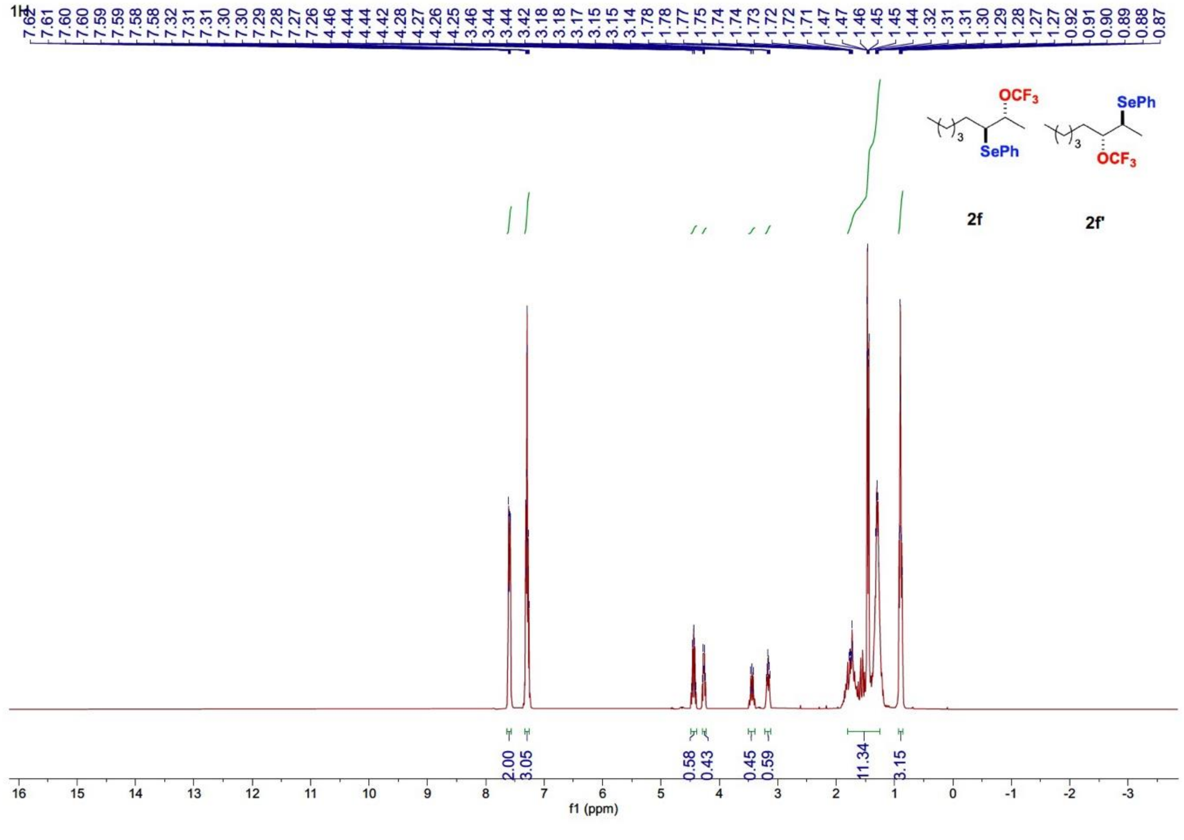
1H
7.62 7.61 7.60 7.59 7.59 7.58 7.32 7.31 7.31 7.30 7.30 7.29 7.28 7.27 7.26 4.46 4.44 4.44 4.42 4.28 4.27 4.26 4.25 3.46 3.44 3.44 3.42 3.18 3.17 3.15 3.15 3.14 1.78 1.78 1.77 1.75 1.74 1.74 1.73 1.72 1.72 1.71 1.47 1.47 1.46 1.45 1.45 1.44 1.32 1.31 1.31 1.30 1.29 1.28 1.27 1.27 0.92 0.91 0.90 0.89 0.88 0.87
OCF3
SePh
2f
2f'
2.00
3.05
0.58
0.43
0.45
0.59
11.34
3.15
16 15 14 13 12 11 10 9 8 7 6 5 4 3 2 1 0 -1 -2 -3
f1 (ppm)

13C

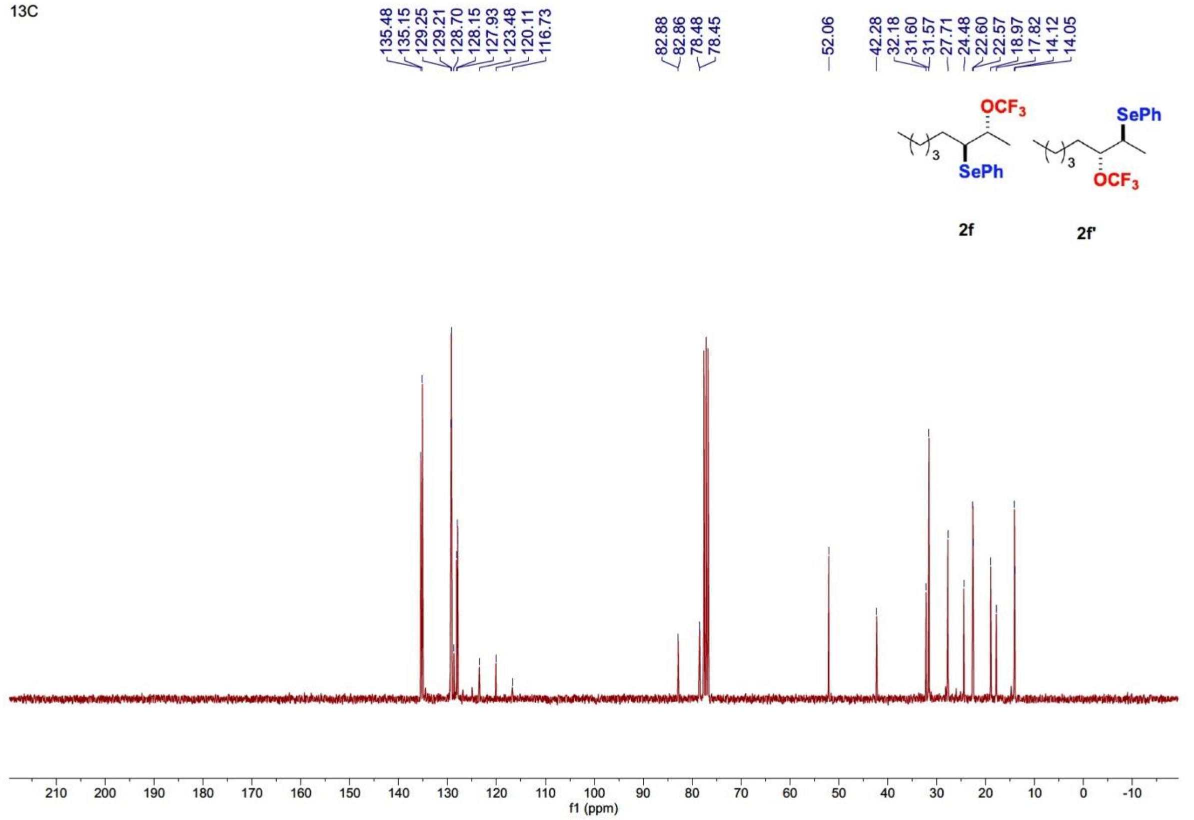

135.48
135.15
129.25
129.21
128.70
128.15
127.93
123.48
120.11
116.73
82.88
82.86
78.48
78.45
52.06
42.28
32.18
31.60
31.57
27.71
24.48
22.60
22.57
18.97
17.82
14.12
14.05
OCF3
SePh
2f
2f'
f1 (ppm)

77Se

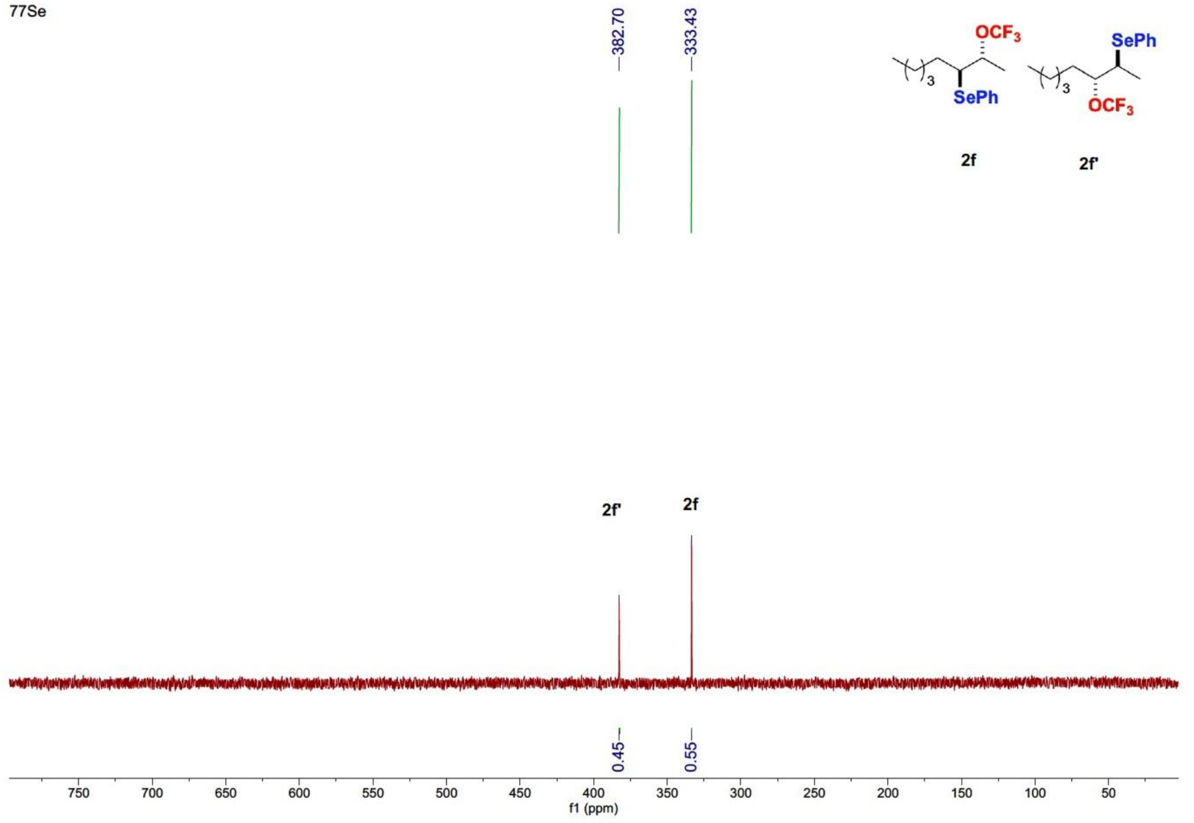

382.70
333.43
OCF3
SePh
2f
2f'
0.45
0.55
f1 (ppm)

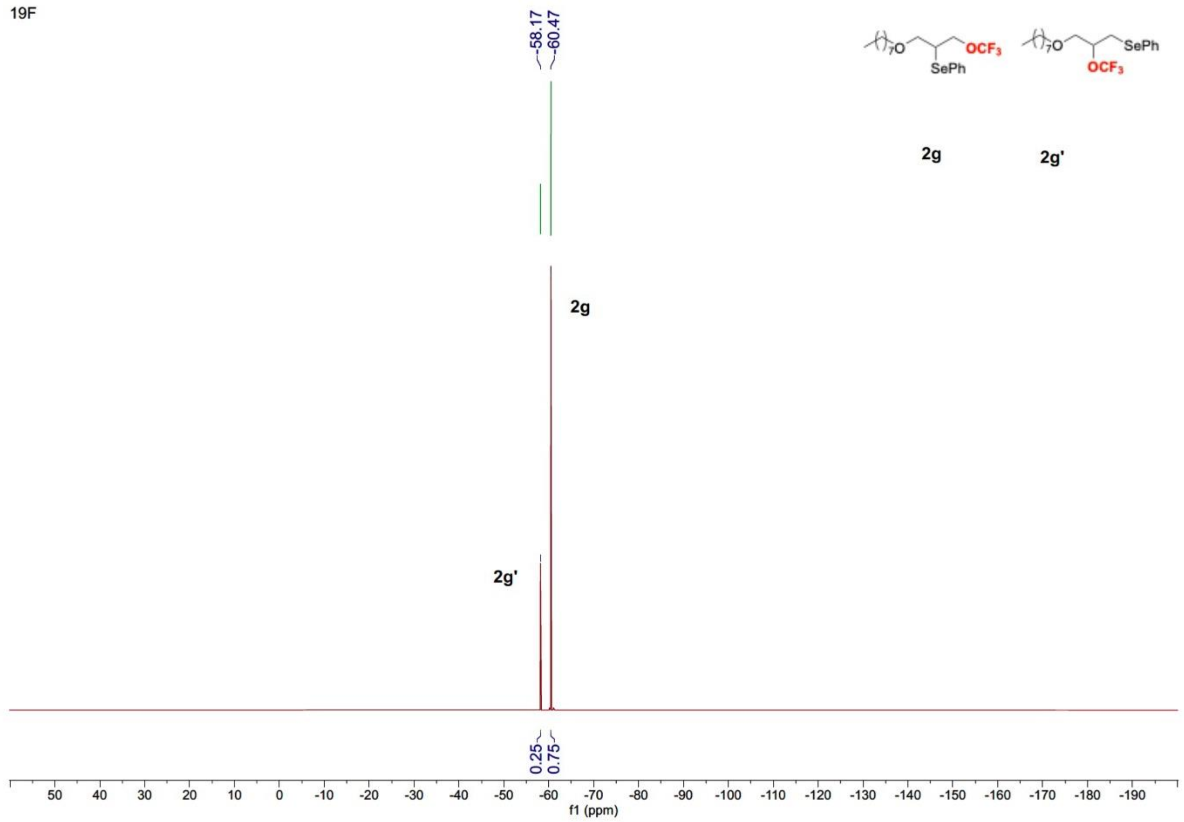
19F
-58.17
-60.47
OCF3
SePh
2g
2g'
0.25
0.75
f1 (ppm)

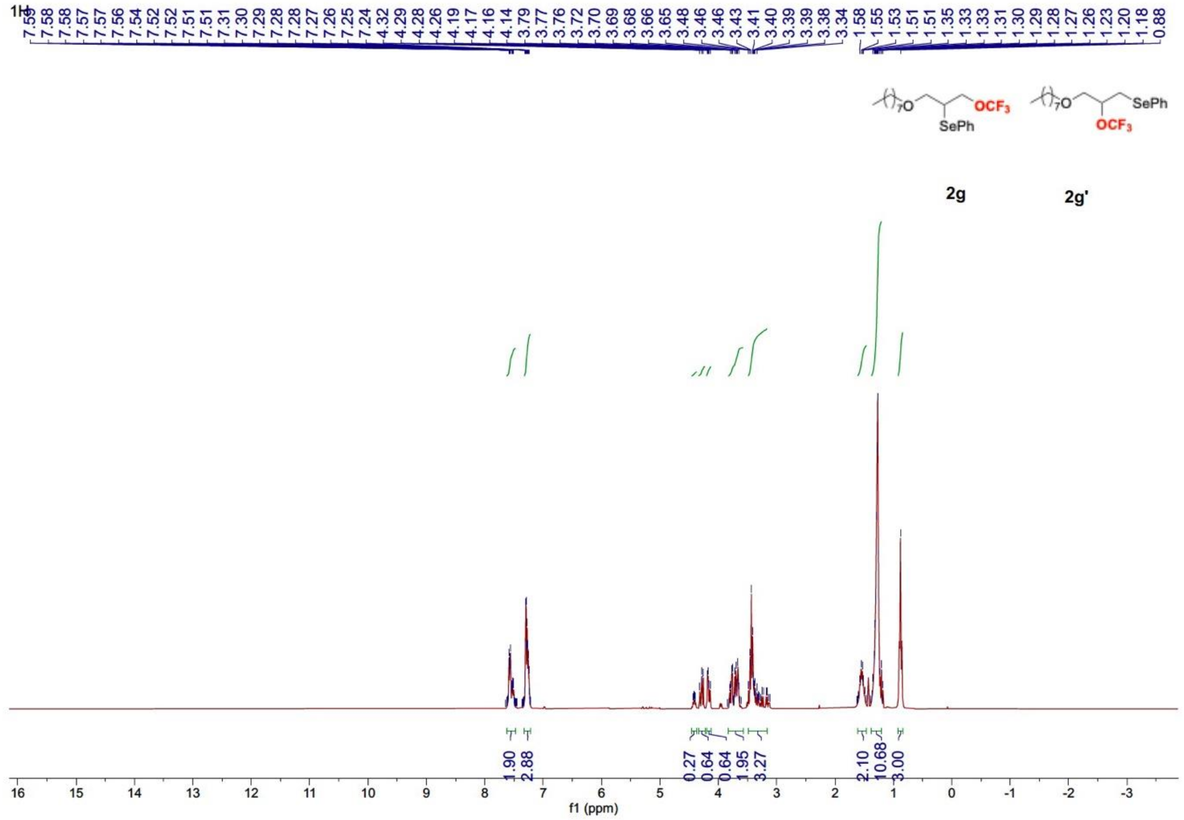
1H
7.59 7.58 7.57 7.57 7.56 7.54 7.52 7.52 7.51 7.51 7.31 7.30 7.29 7.28 7.28 7.27 7.26 7.25 7.24 4.32 4.29 4.28 4.26 4.19 4.17 4.16 4.14 3.79 3.77 3.76 3.72 3.70 3.69 3.68 3.66 3.65 3.48 3.46 3.46 3.43 3.41 3.40 3.39 3.38 3.34 1.58 1.55 1.53 1.51 1.51 1.35 1.33 1.31 1.30 1.29 1.28 1.27 1.26 1.23 1.20 1.18 0.88
OCF3
SePh
2g
2g'
1.90
2.88
0.27
0.64
0.64
1.95
3.27
2.10
10.68
3.00
f1 (ppm)

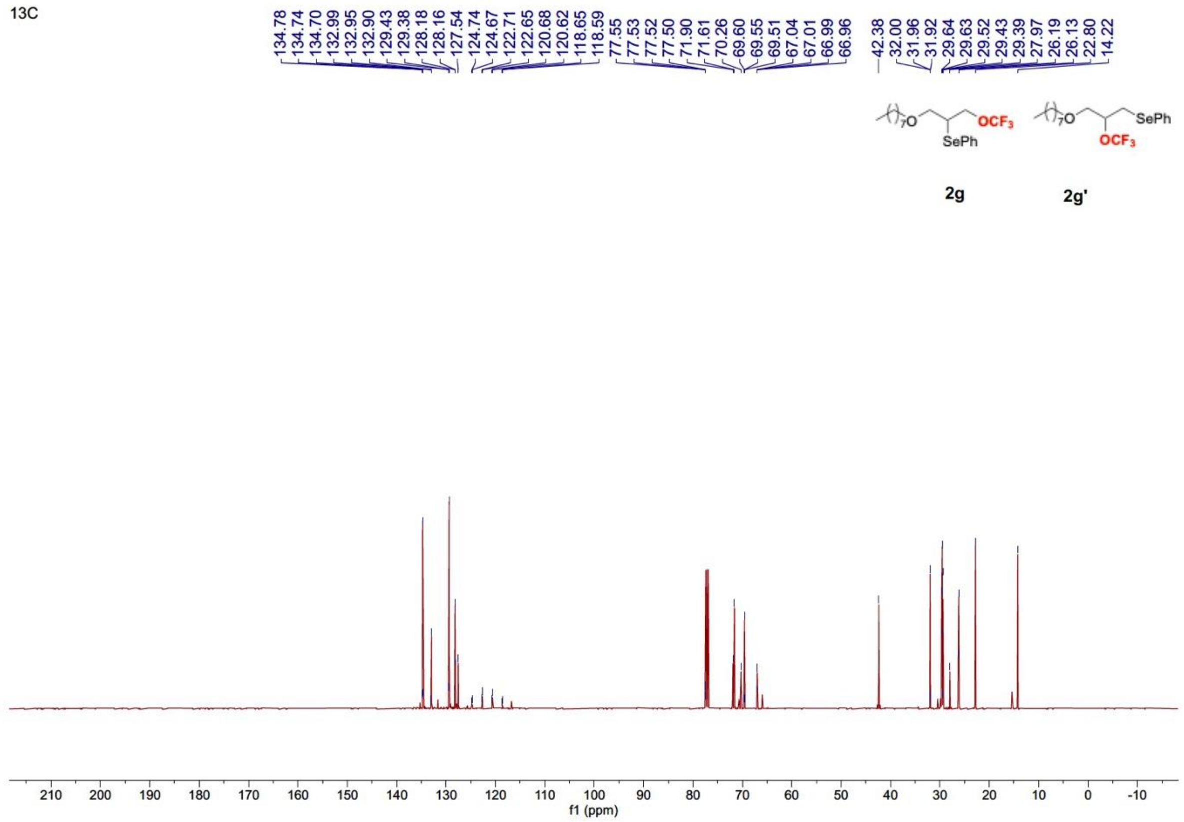
13C
134.78
134.74
134.70
132.99
132.95
132.90
129.43
129.38
128.18
128.16
127.54
124.74
124.67
122.71
122.65
120.68
120.62
118.65
118.59
77.55
77.53
77.52
77.50
71.90
71.61
70.26
69.60
69.55
69.51
67.04
67.01
66.99
66.96
42.38
32.00
31.96
31.92
29.64
29.63
29.52
29.43
29.39
27.97
26.19
26.13
22.80
14.22
OCF3
SePh
2g
2g'
f1 (ppm)

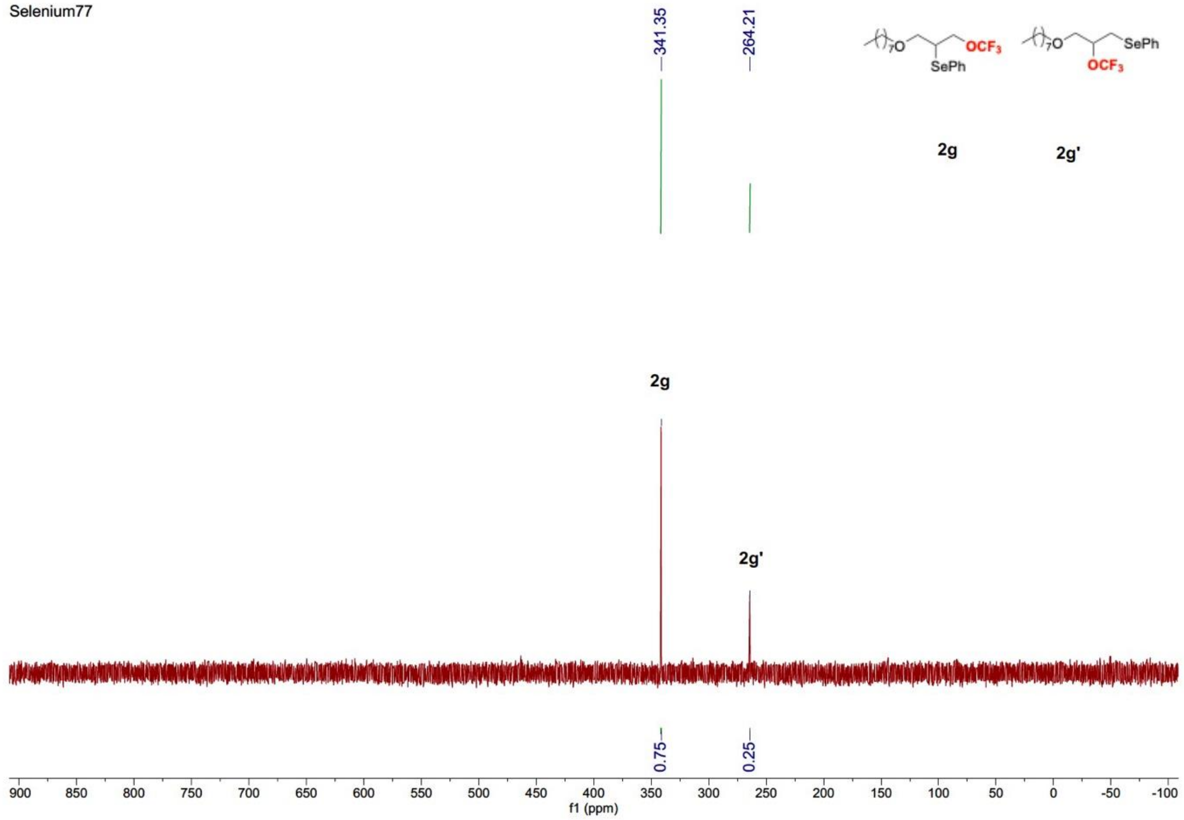
Selenium77
341.35
264.21
OCF3
SePh
2g
2g'
0.75
0.25
f1 (ppm)

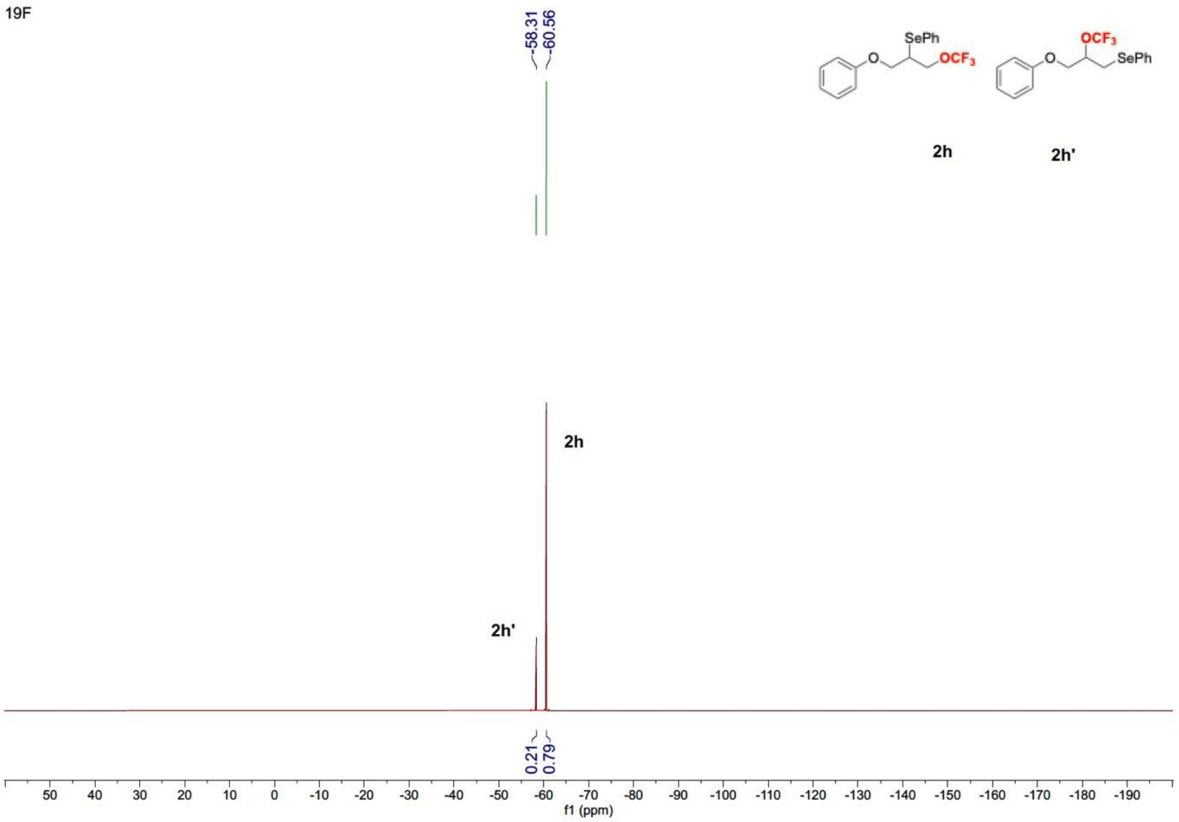
19F
-58.31
-60.56
SePh
OCF3
OCF3
SePh
2h
2h'
2h
2h'
0.21
0.79
50 40 30 20 10 0 -10 -20 -30 -40 -50 -60 -70 -80 -90 -100 -110 -120 -130 -140 -150 -160 -170 -180 -190
f1 (ppm)

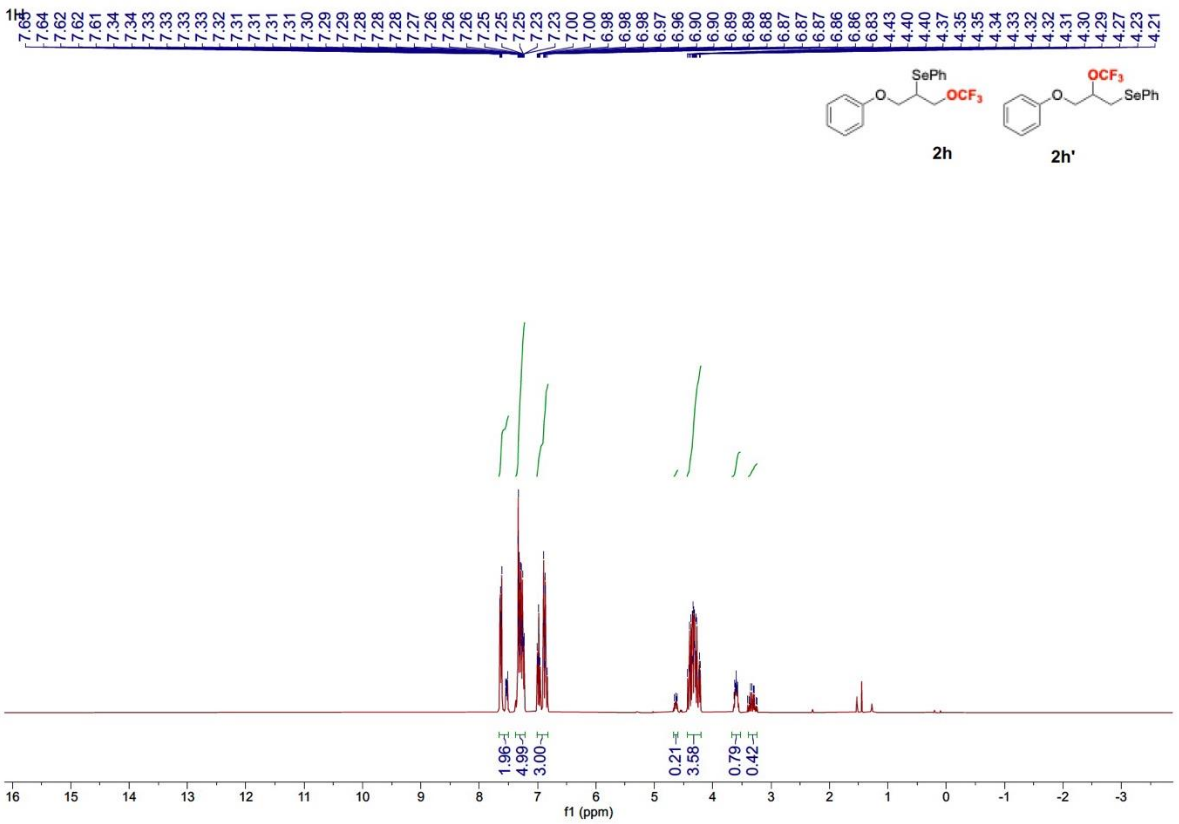
1H
7.65 7.64 7.62 7.61 7.34 7.34 7.33 7.33 7.33 7.32 7.31 7.31 7.31 7.30 7.29 7.29 7.28 7.28 7.27 7.26 7.26 7.25 7.25 7.25 7.23 7.23 7.00 7.00 6.98 6.98 6.97 6.96 6.90 6.90 6.89 6.88 6.87 6.87 6.86 6.83 4.43 4.40 4.37 4.35 4.34 4.33 4.32 4.32 4.31 4.30 4.29 4.27 4.23 4.21
SePh
OCF3
OCF3
SePh
2h
2h'
1.96
4.99
3.00
0.21
3.58
0.79
0.42
16 15 14 13 12 11 10 9 8 7 6 5 4 3 2 1 0 -1 -2 -3
f1 (ppm)

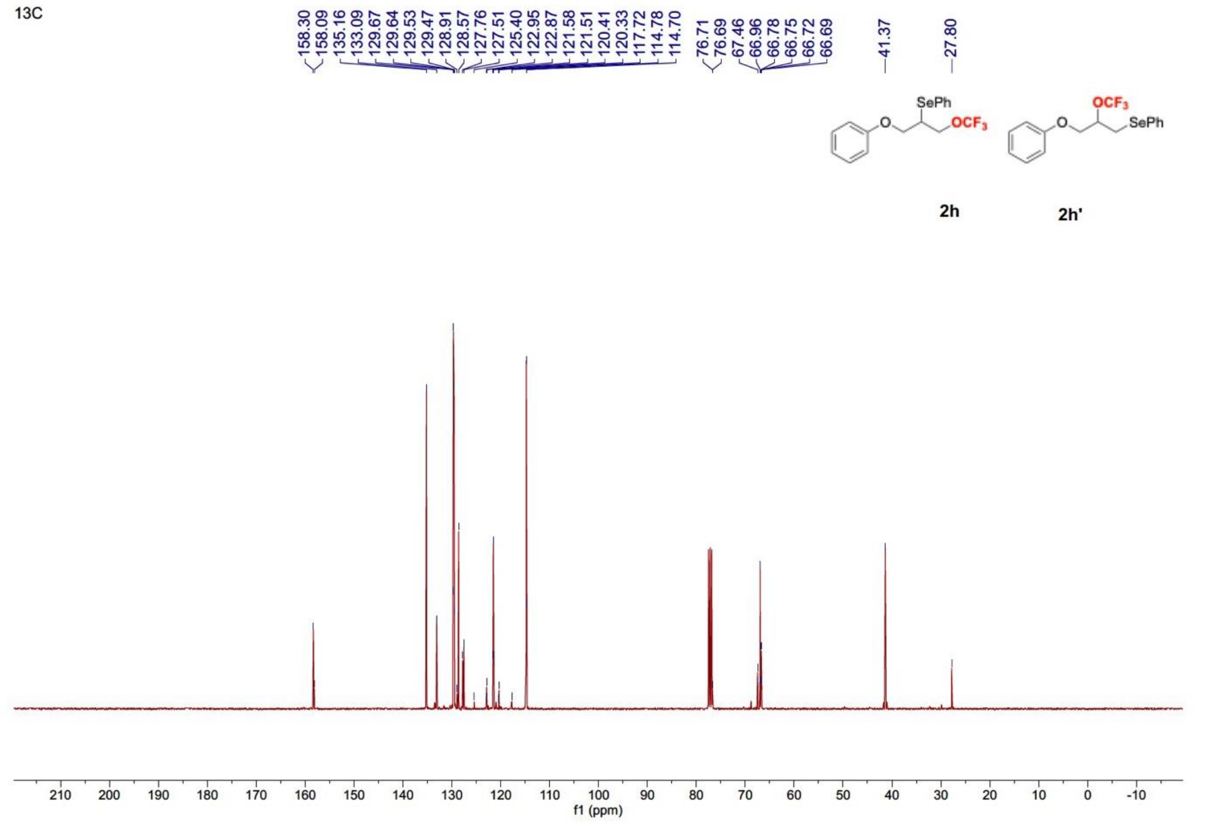

13C
158.30
158.09
135.16
133.09
129.67
129.64
129.53
129.47
128.91
128.57
127.76
127.51
125.40
122.95
122.87
121.58
121.51
120.41
120.33
117.72
114.78
114.70
76.71
76.69
67.46
66.96
66.78
66.75
66.72
66.69
41.37
27.80
SePh
OCF3
SePh
2h
2h'
210 200 190 180 170 160 150 140 130 120 110 100 90 80 70 60 50 40 30 20 10 0 -10
f1 (ppm)

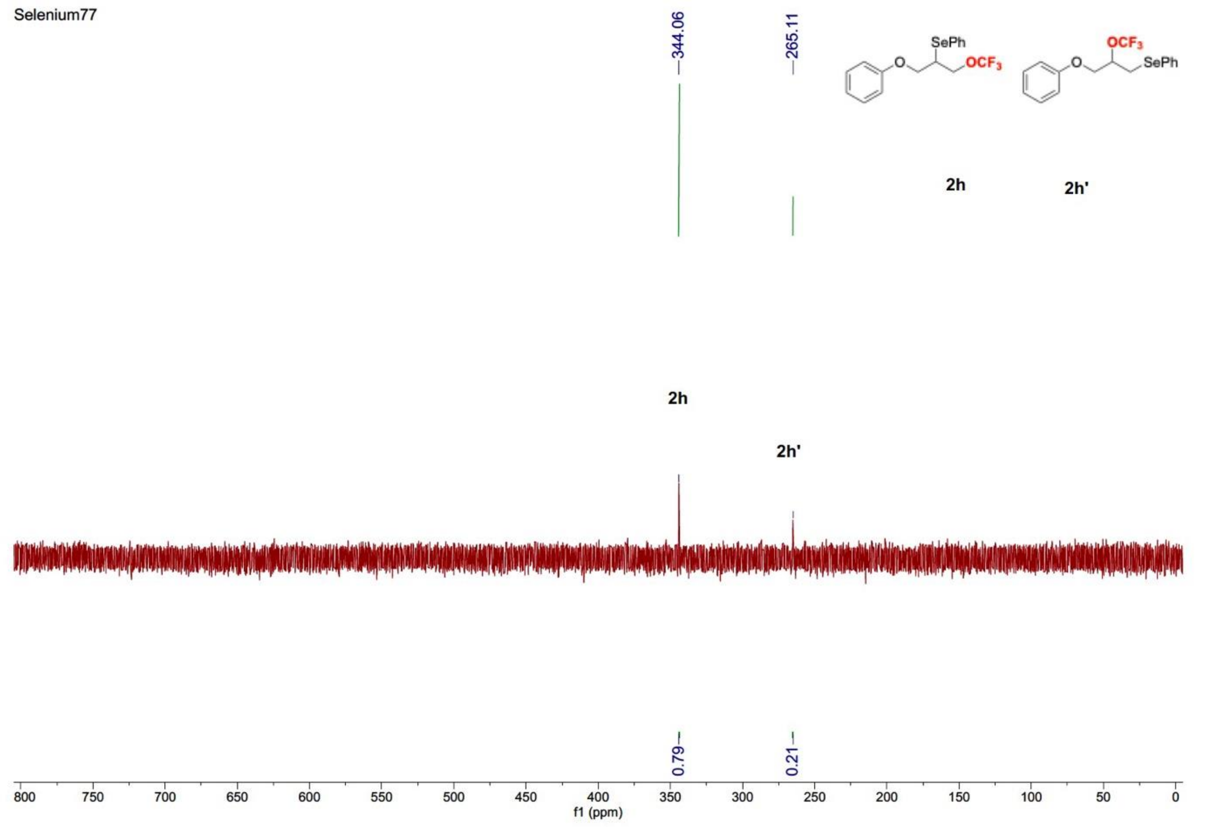

Selenium77
344.06
265.11
SePh
OCF3
SePh
2h
2h'
2h
2h'
0.79
0.21
800 750 700 650 600 550 500 450 400 350 300 250 200 150 100 50 0
f1 (ppm)

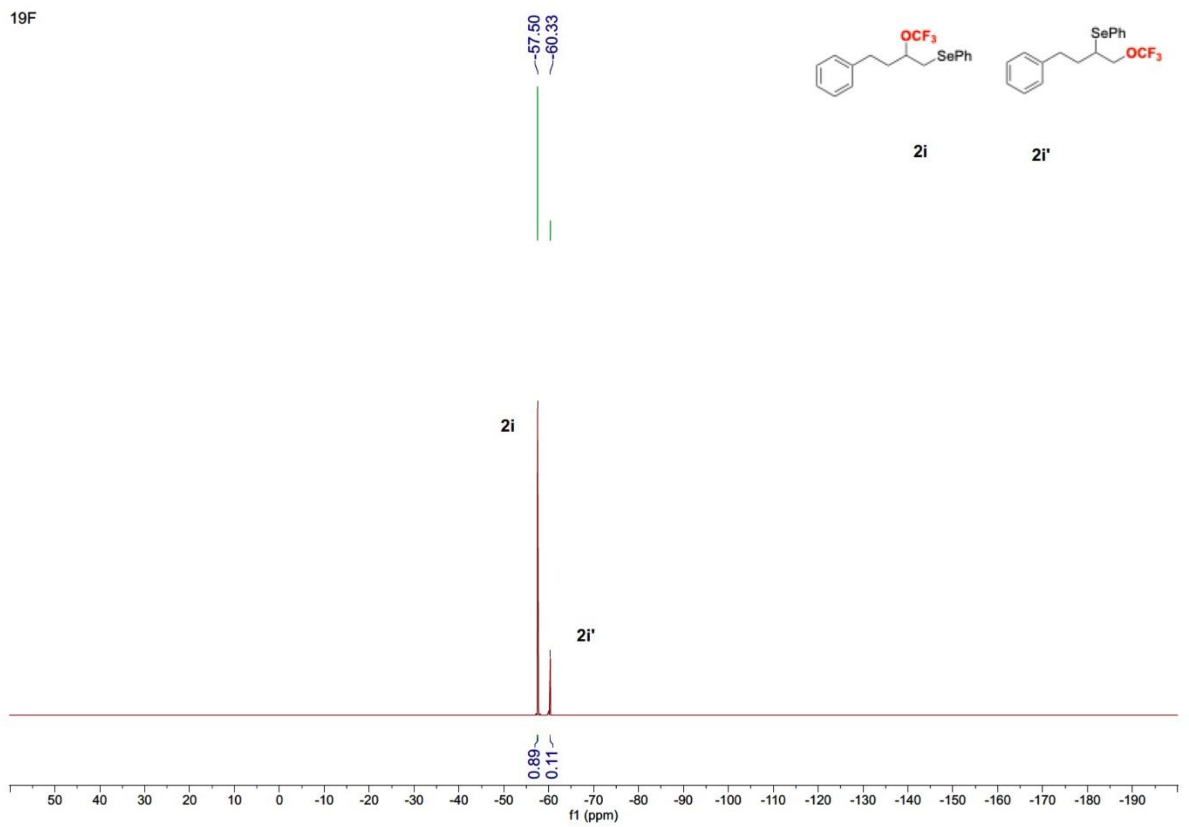
19F
-57.50
-60.33
OCF3
SePh
SePh
OCF3
2i
2i'
2i
2i'
0.89
0.11
f1 (ppm)

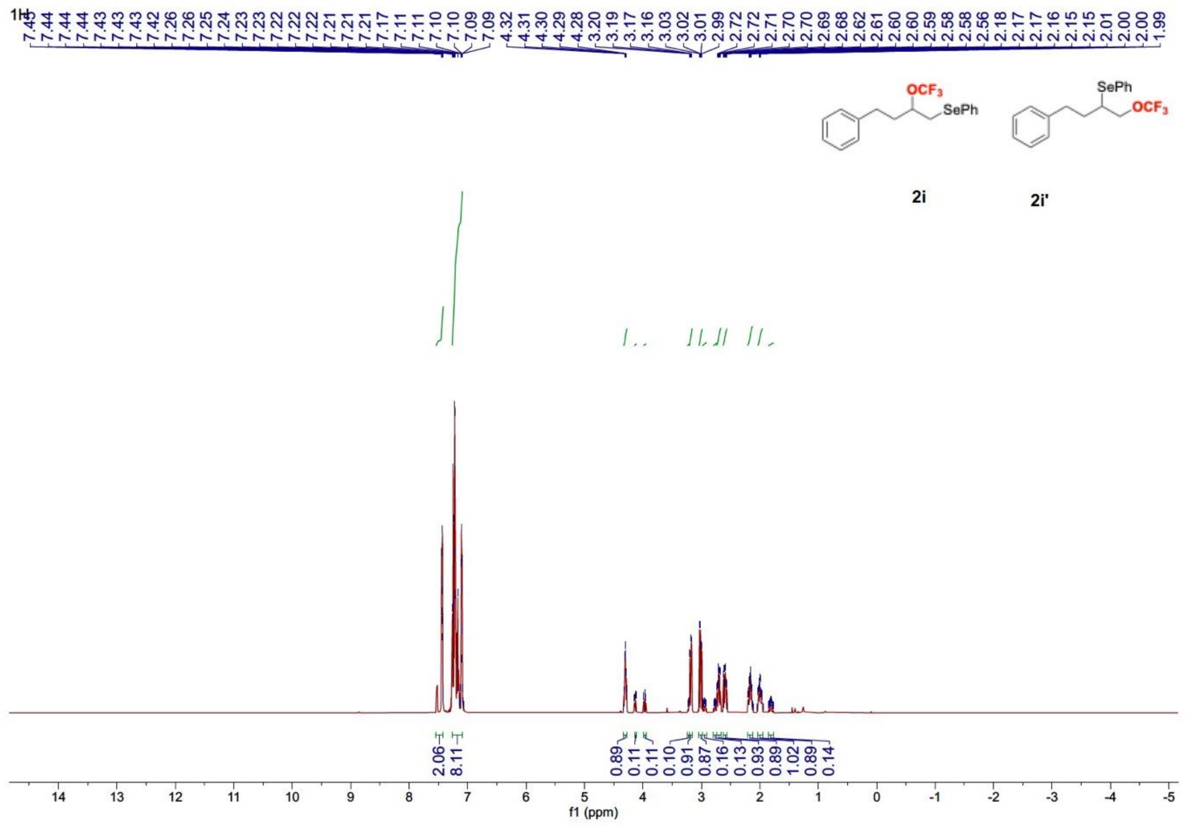
1H
7.45 7.44 7.44 7.43 7.43 7.42 7.26 7.26 7.25 7.24 7.23 7.22 7.22 7.21 7.21 7.17 7.11 7.11 7.10 7.09 7.09 4.32 4.31 4.30 4.29 4.28 3.20 3.19 3.17 3.16 3.03 3.02 3.01 2.99 2.72 2.71 2.70 2.69 2.68 2.62 2.61 2.60 2.60 2.59 2.58 2.58 2.56 2.18 2.17 2.17 2.16 2.15 2.15 2.01 2.00 2.00 1.99
OCF3
SePh
SePh
OCF3
2i
2i'
2.06
8.11
0.89
0.11
0.11
0.10
0.91
0.87
0.16
0.13
0.93
0.89
1.02
0.89
0.14
f1 (ppm)

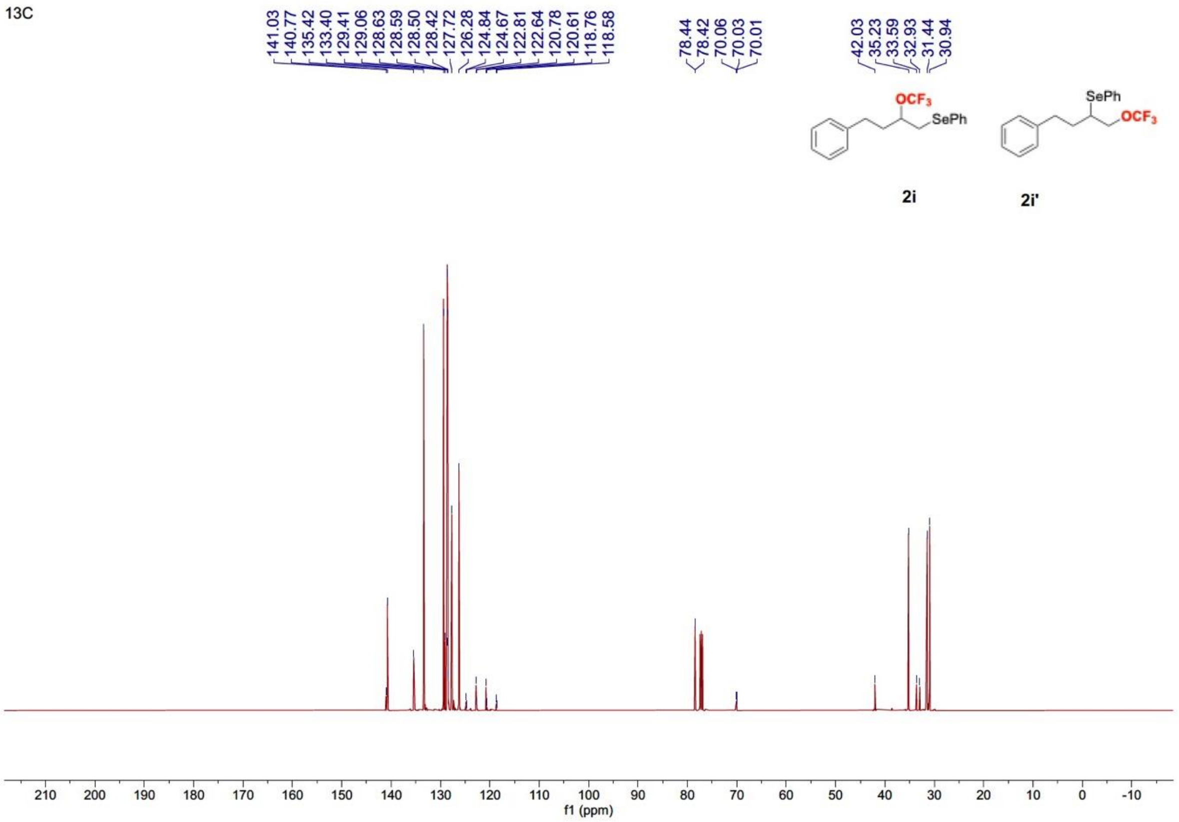
13C
141.03
140.77
135.42
133.40
129.41
129.06
128.63
128.59
128.50
128.42
127.72
126.28
124.84
124.67
122.81
122.64
120.78
120.61
118.76
118.58
78.44
78.42
70.06
70.03
70.01
42.03
35.23
33.59
32.93
31.44
30.94
OCF3
SePh
2i
2i'
f1 (ppm)

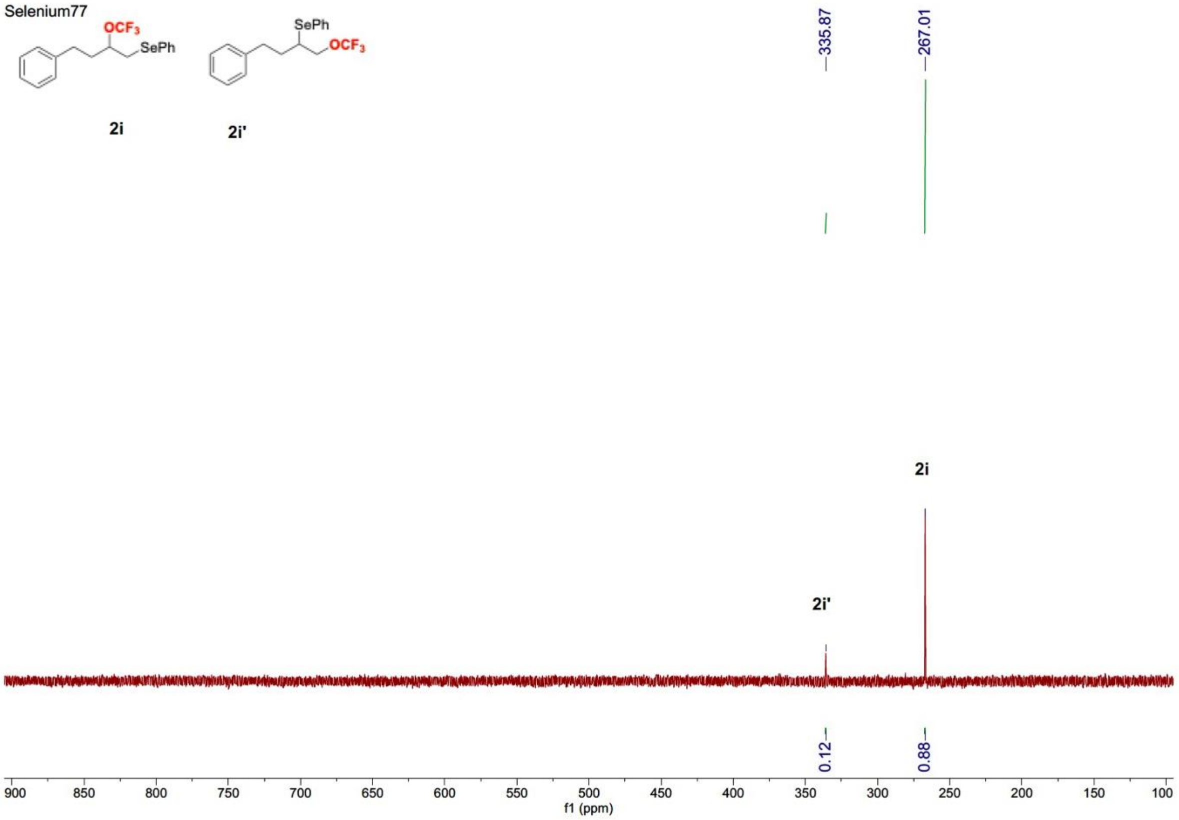
Selenium77
OCF3
SePh
2i
2i'
335.87
267.01
0.12
0.88
f1 (ppm)

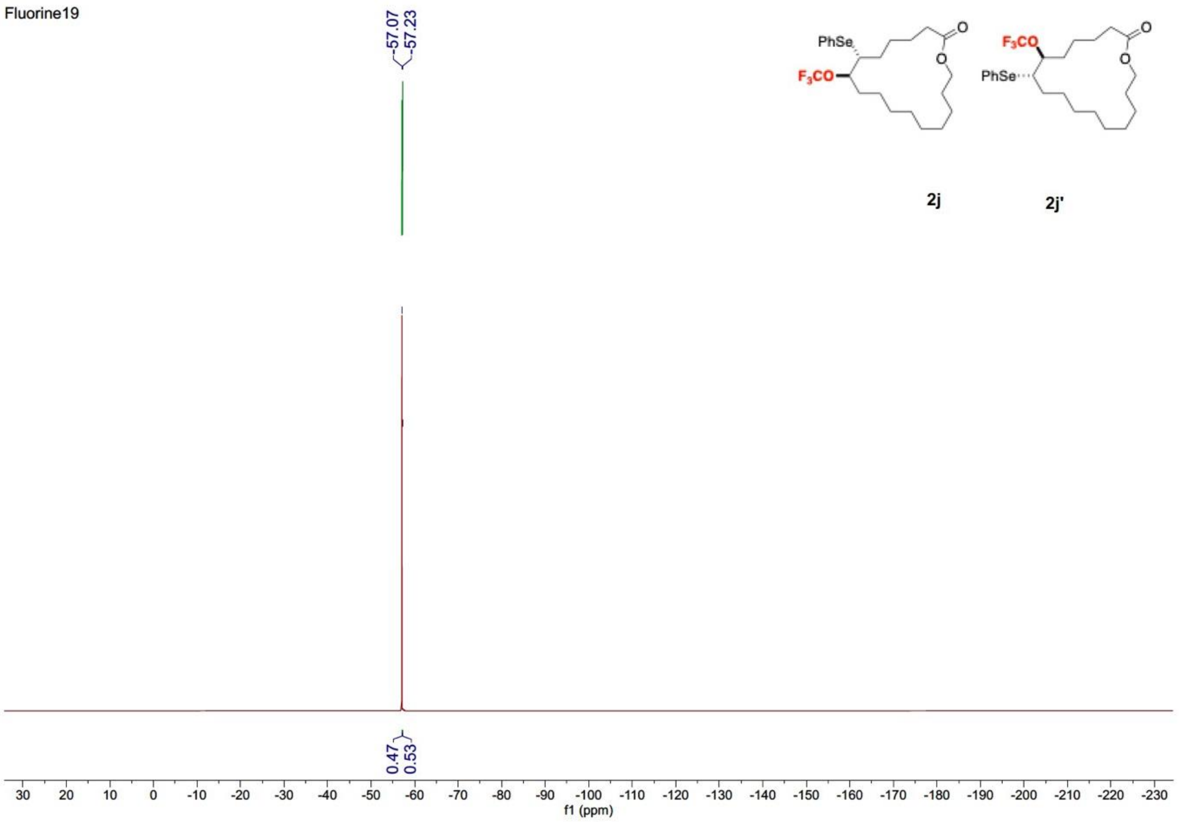
Fluorine19
-57.07
-57.23
PhSe
F3CO
2j
2j'
0.47
0.53
30 20 10 0 -10 -20 -30 -40 -50 -60 -70 -80 -90 -100 -110 -120 -130 -140 -150 -160 -170 -180 -190 -200 -210 -220 -230
f1 (ppm)

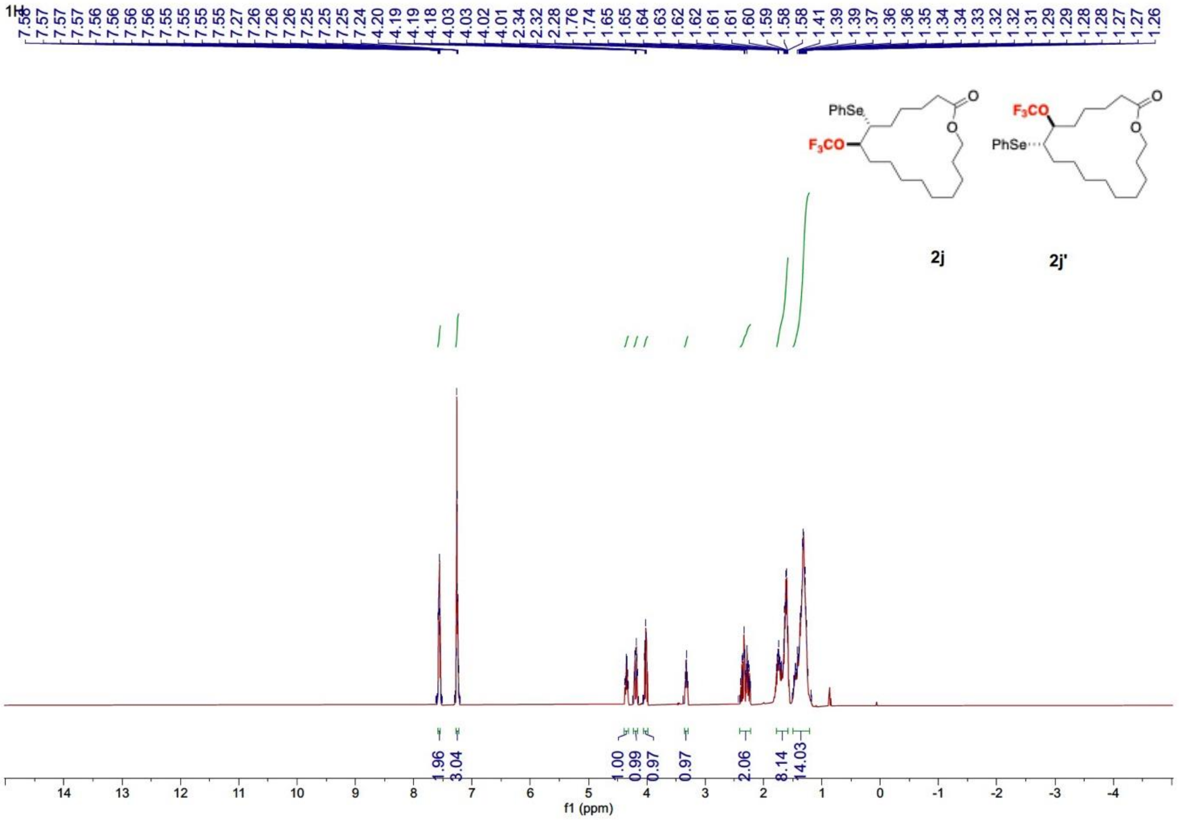
1H
7.58 7.57 7.57 7.56 7.56 7.56 7.55 7.55 7.55 7.27 7.26 7.26 7.25 7.25 7.24 4.20 4.19 4.18 4.03 4.02 4.01 2.34 2.32 2.28 1.76 1.74 1.65 1.65 1.64 1.63 1.62 1.62 1.61 1.61 1.60 1.59 1.58 1.58 1.41 1.39 1.39 1.37 1.36 1.36 1.35 1.34 1.34 1.33 1.32 1.32 1.31 1.29 1.29 1.28 1.28 1.27 1.27 1.26
PhSe
F3CO
2j
2j'
1.96
3.04
1.00
0.99
0.97
0.97
2.06
8.14
14.03
14 13 12 11 10 9 8 7 6 5 4 3 2 1 0 -1 -2 -3 -4
f1 (ppm)

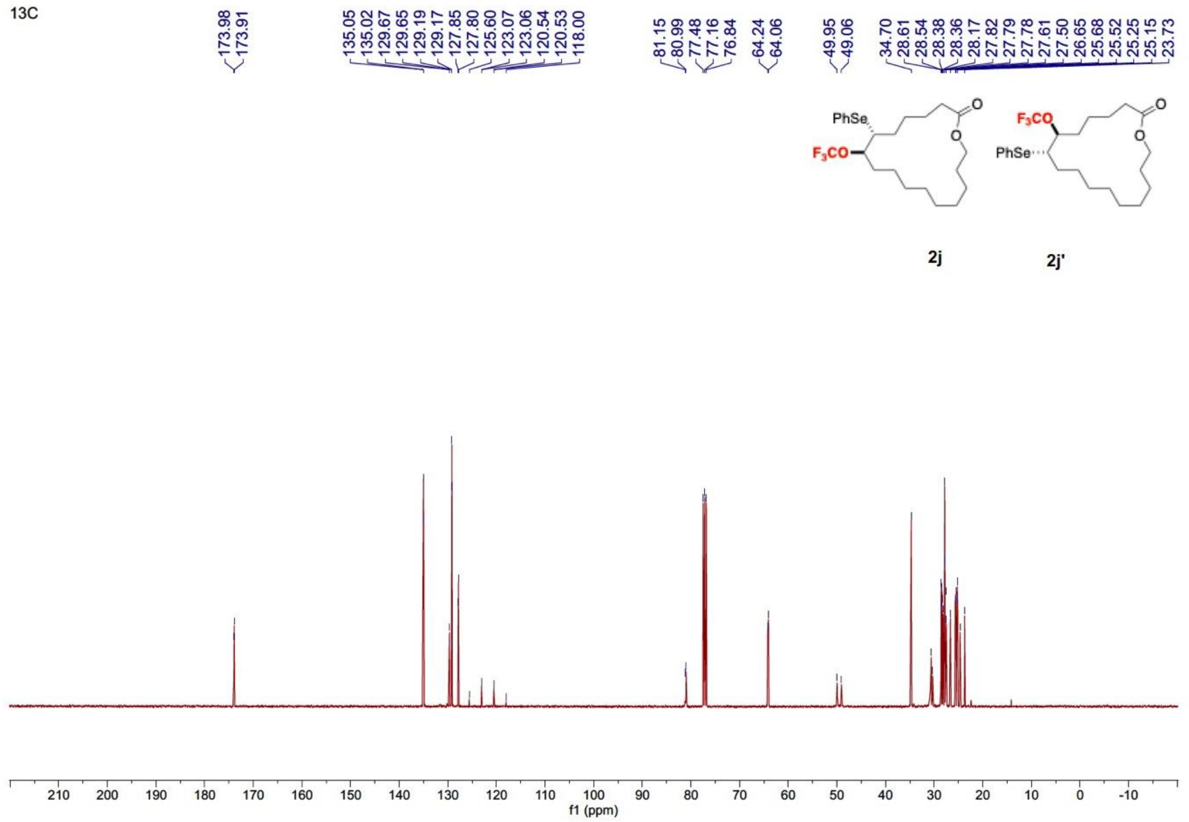
13C
173.98
173.91
135.05
135.02
129.67
129.65
129.19
129.17
127.85
127.80
125.60
123.07
123.06
120.54
120.53
118.00
81.15
80.99
77.48
77.16
76.84
64.24
64.06
49.95
49.06
34.70
28.61
28.54
28.38
28.36
28.17
27.82
27.79
27.78
27.61
27.50
26.65
25.68
25.52
25.25
25.15
23.73
PhSe
F3CO
F3CO
PhSe
2j
2j'
210 200 190 180 170 160 150 140 130 120 110 100 90 80 70 60 50 40 30 20 10 0 -10
f1 (ppm)

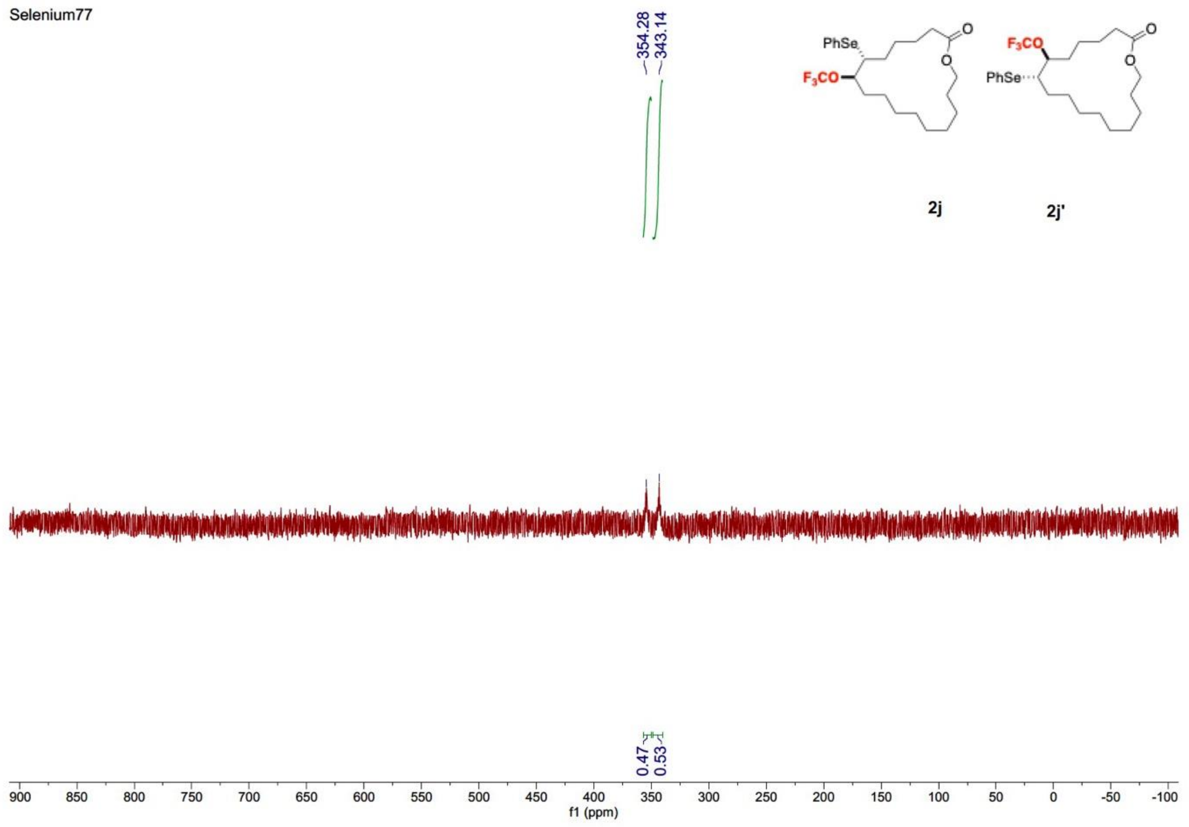
Selenium77
354.28
343.14
PhSe
F3CO
F3CO
PhSe
2j
2j'
0.47
0.53
900 850 800 750 700 650 600 550 500 450 400 350 300 250 200 150 100 50 0 -50 -100
f1 (ppm)

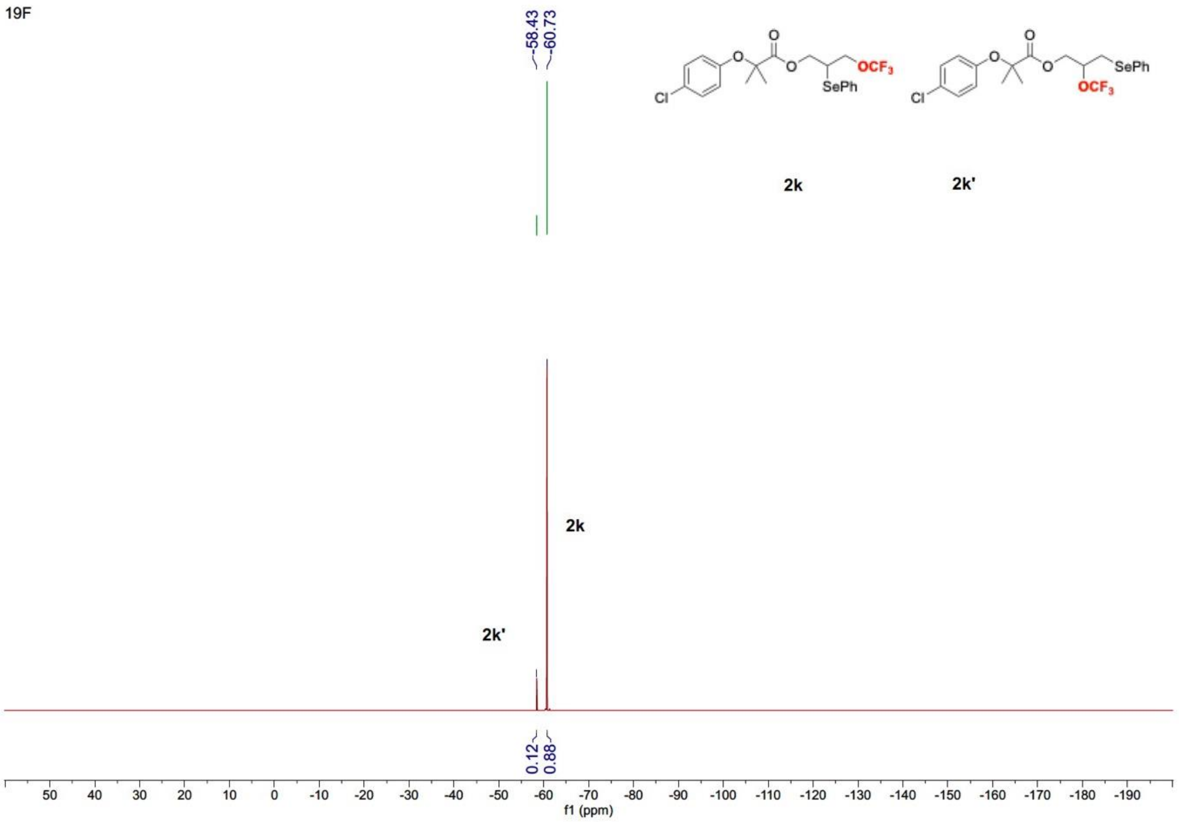
19F
-58.43
-60.73
OCF3
SePh
Cl
2k
2k'
0.12
0.88
f1 (ppm)

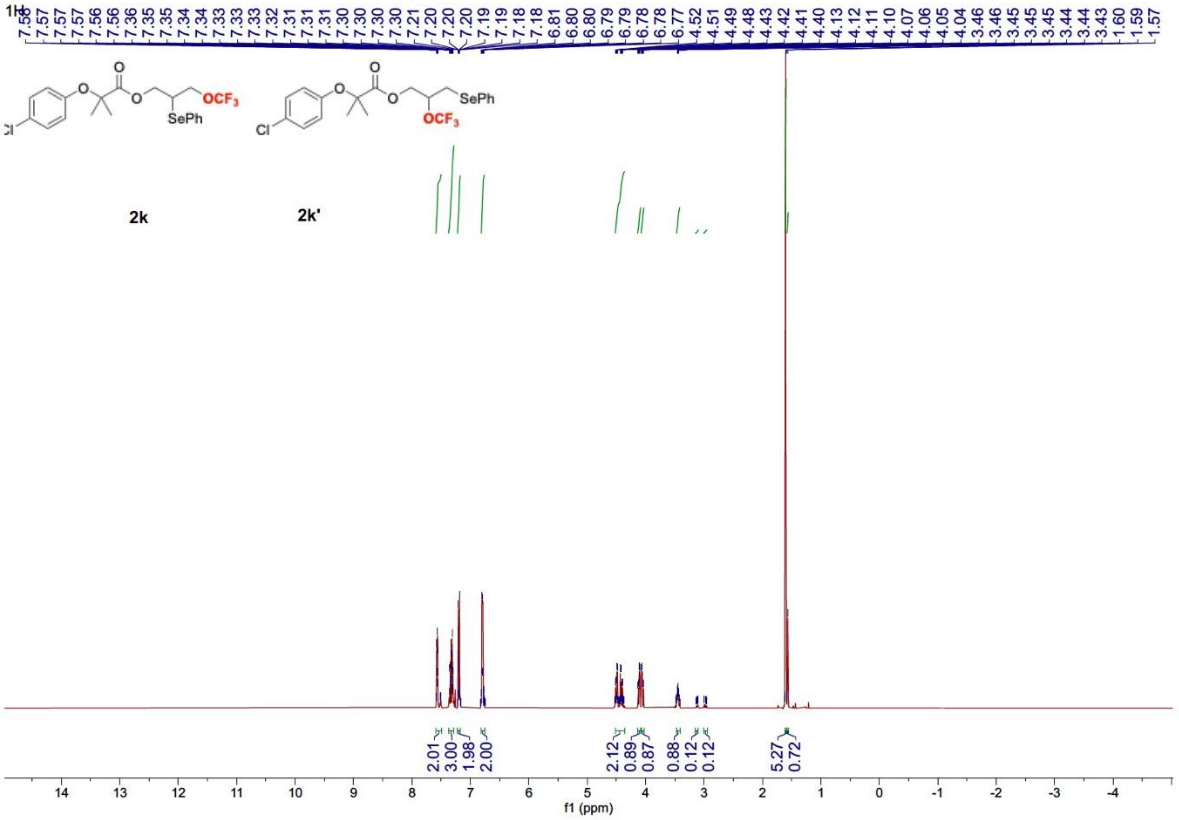
1H
OCF3
SePh
Cl
2k
2k'
2.01
3.00
1.98
2.00
2.12
0.89
0.87
0.88
0.12
0.12
5.27
0.72
f1 (ppm)

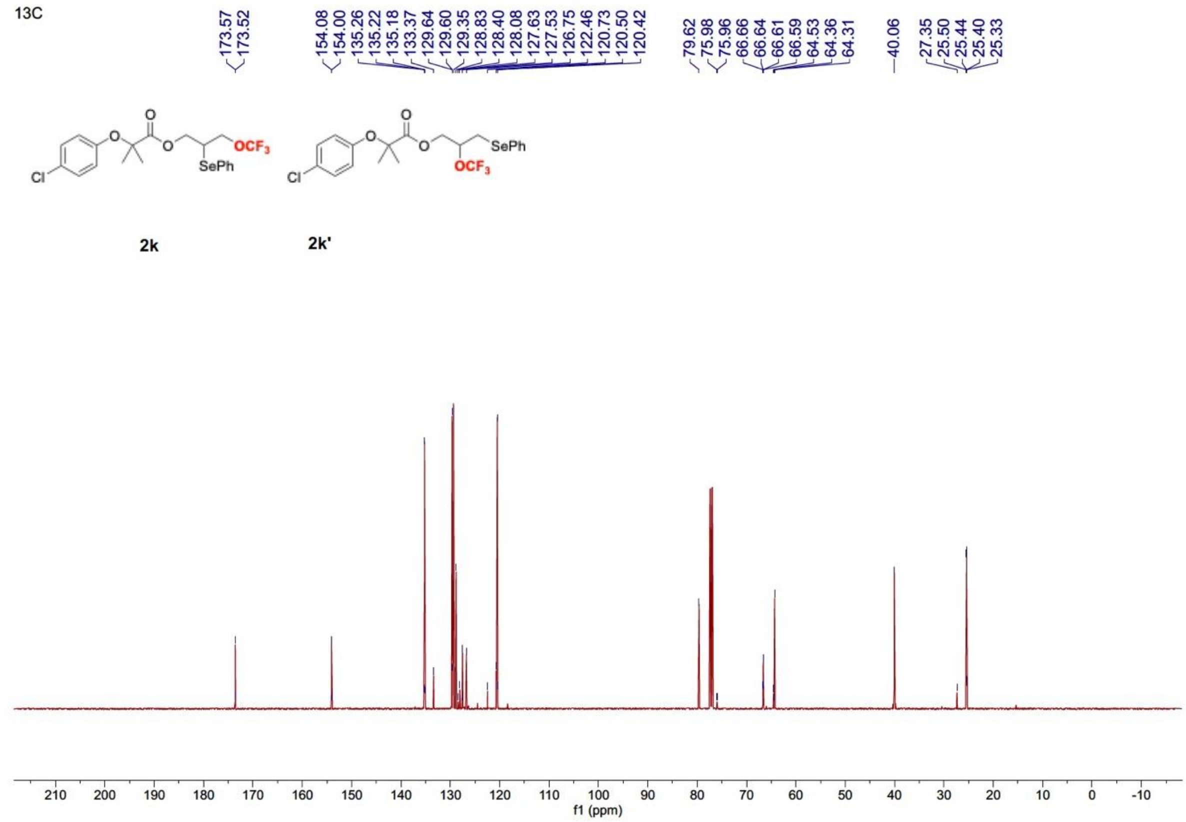
13C
173.57
173.52
154.08
154.00
135.26
135.22
135.18
133.37
129.64
129.60
129.35
128.83
128.40
128.08
127.63
127.53
126.75
122.46
120.73
120.50
120.42
79.62
75.98
75.96
66.66
66.64
66.61
66.59
64.53
64.36
64.31
40.06
27.35
25.50
25.44
25.40
25.33
OCF3
SePh
Cl
2k
2k'
f1 (ppm)

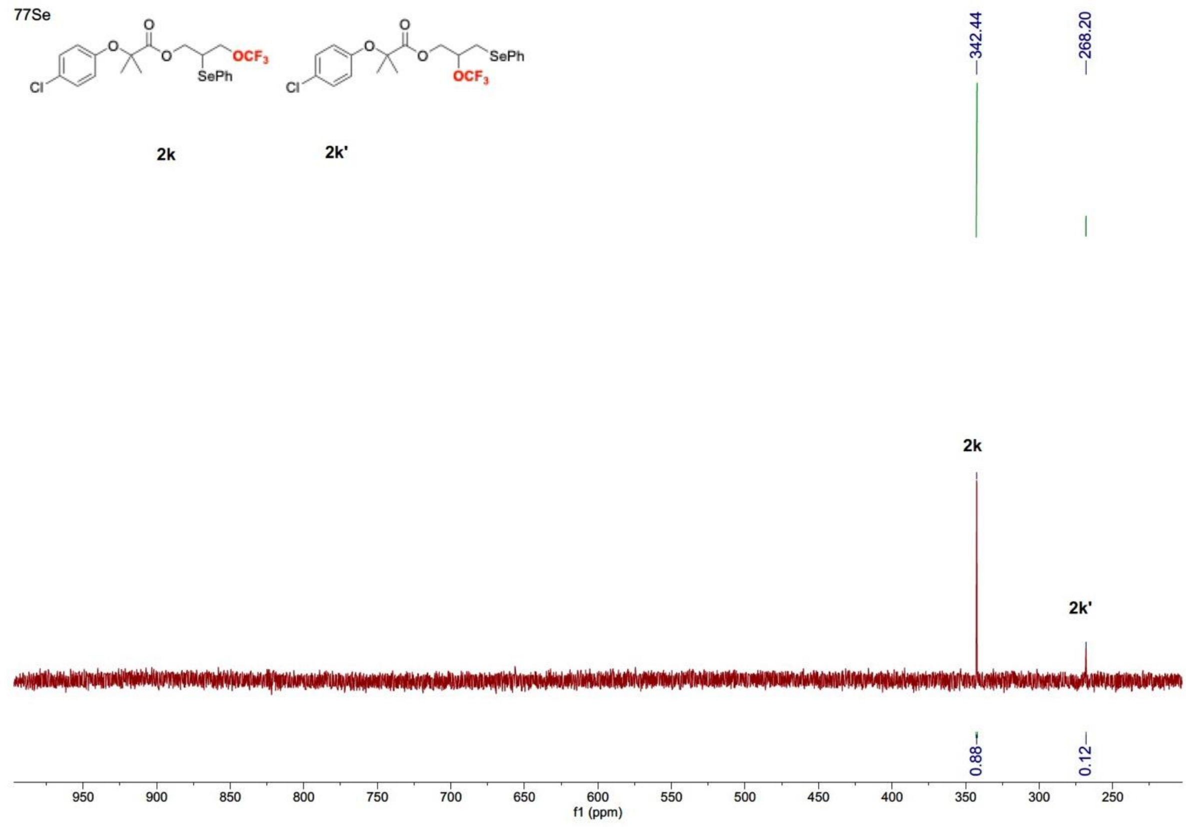
77Se
342.44
268.20
OCF3
SePh
Cl
2k
2k'
0.88
0.12
f1 (ppm)

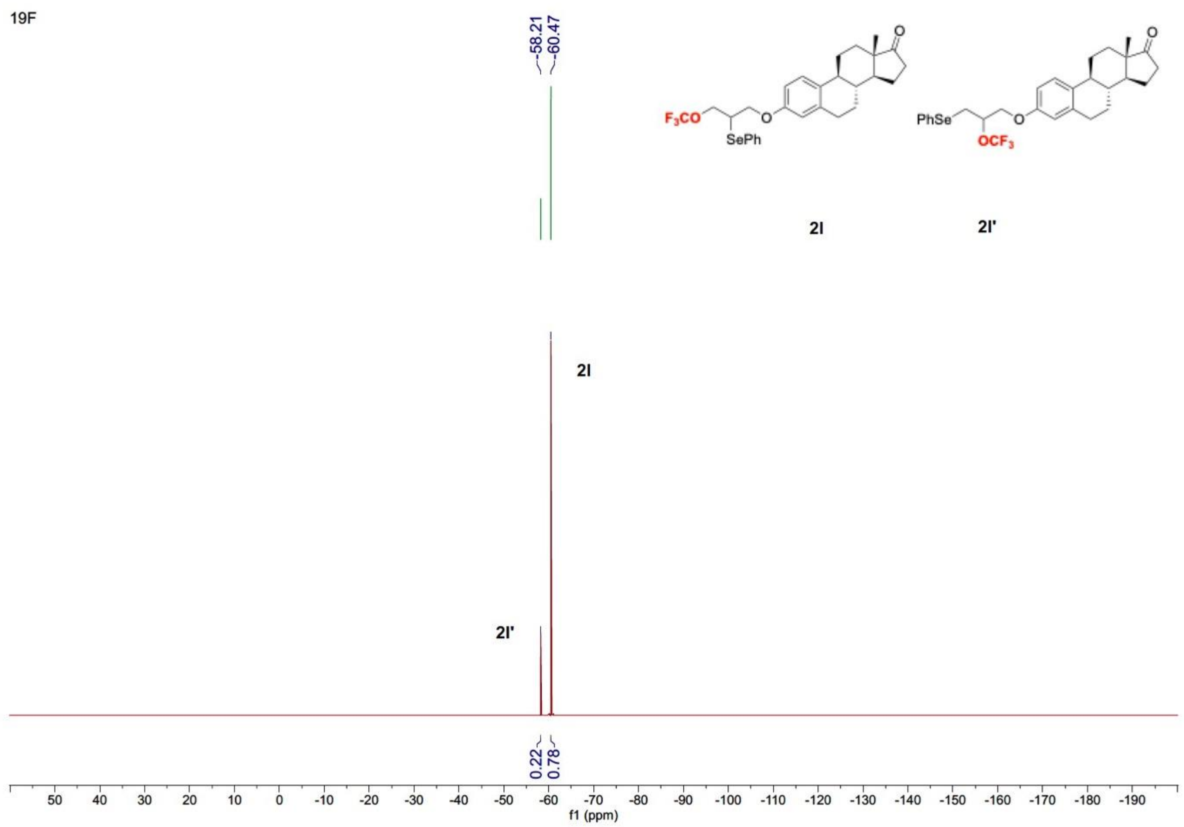
19F
-58.21
-60.47
F3CO
SePh
PhSe
OCF3
2l
2l'
2l
2l'
0.22
0.78
50 40 30 20 10 0 -10 -20 -30 -40 -50 -60 -70 -80 -90 -100 -110 -120 -130 -140 -150 -160 -170 -180 -190
f1 (ppm)

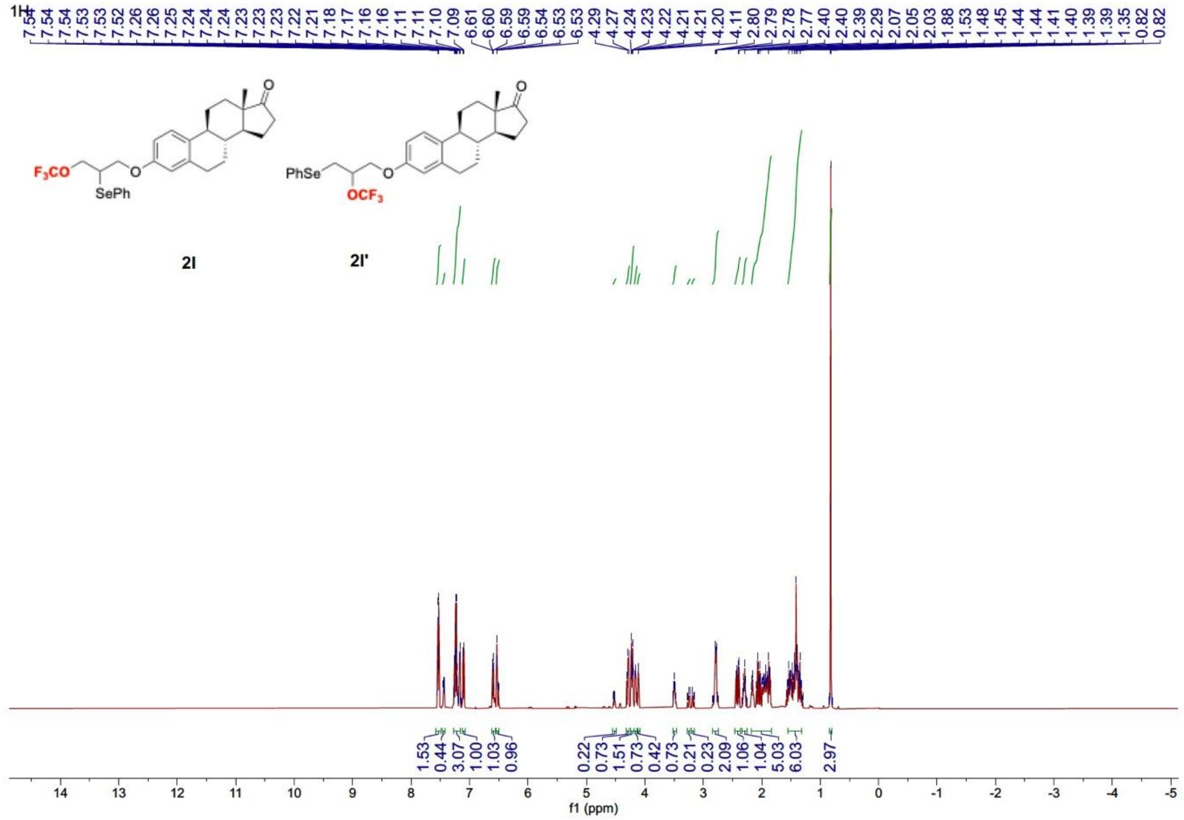
1H
7.54 7.54 7.53 7.53 7.52 7.26 7.25 7.24 7.24 7.24 7.23 7.23 7.22 7.21 7.18 7.17 7.16 7.11 7.10 7.09 6.61 6.60 6.59 6.59 6.54 6.53 4.29 4.27 4.24 4.23 4.22 4.21 4.21 4.20 4.11 2.80 2.79 2.78 2.77 2.40 2.40 2.39 2.29 2.07 2.05 2.03 1.88 1.53 1.48 1.45 1.44 1.41 1.40 1.39 1.35 0.82 0.82
F3CO
SePh
PhSe
OCF3
2l
2l'
1.53 0.44 3.07 1.00 1.03 0.96 0.22 0.73 1.51 0.73 0.42 0.73 0.21 0.23 2.09 1.06 1.04 5.03 6.03 2.97
14 13 12 11 10 9 8 7 6 5 4 3 2 1 0 -1 -2 -3 -4 -5
f1 (ppm)

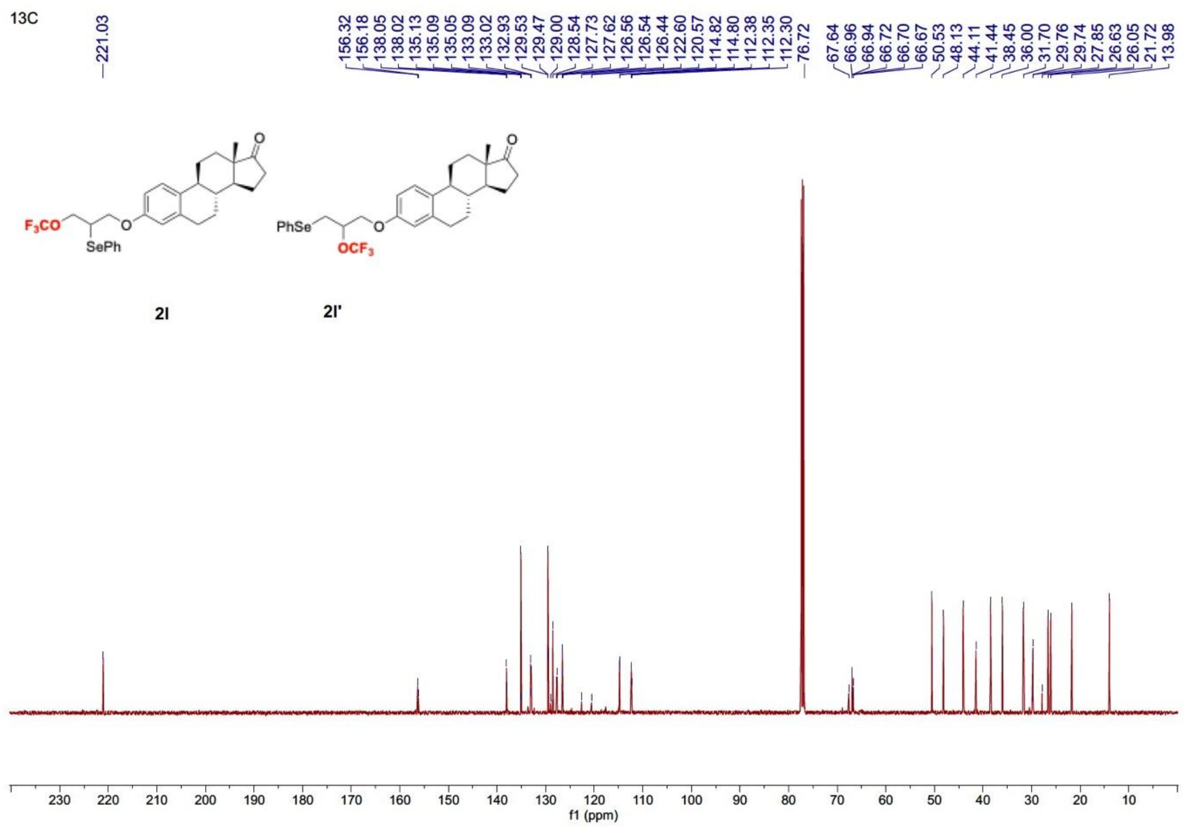
13C
221.03
156.32
156.18
138.05
138.02
135.13
135.09
135.05
133.09
133.02
132.93
129.53
129.47
129.00
128.54
127.73
127.62
126.56
126.54
126.44
122.60
120.57
114.82
114.80
112.38
112.35
112.30
76.72
67.64
66.96
66.94
66.72
66.70
66.67
50.53
48.13
44.11
41.44
38.45
36.00
31.70
29.76
29.74
27.85
26.63
26.05
21.72
13.98
F3CO
SePh
PhSe
OCF3
2l
2l'
f1 (ppm)

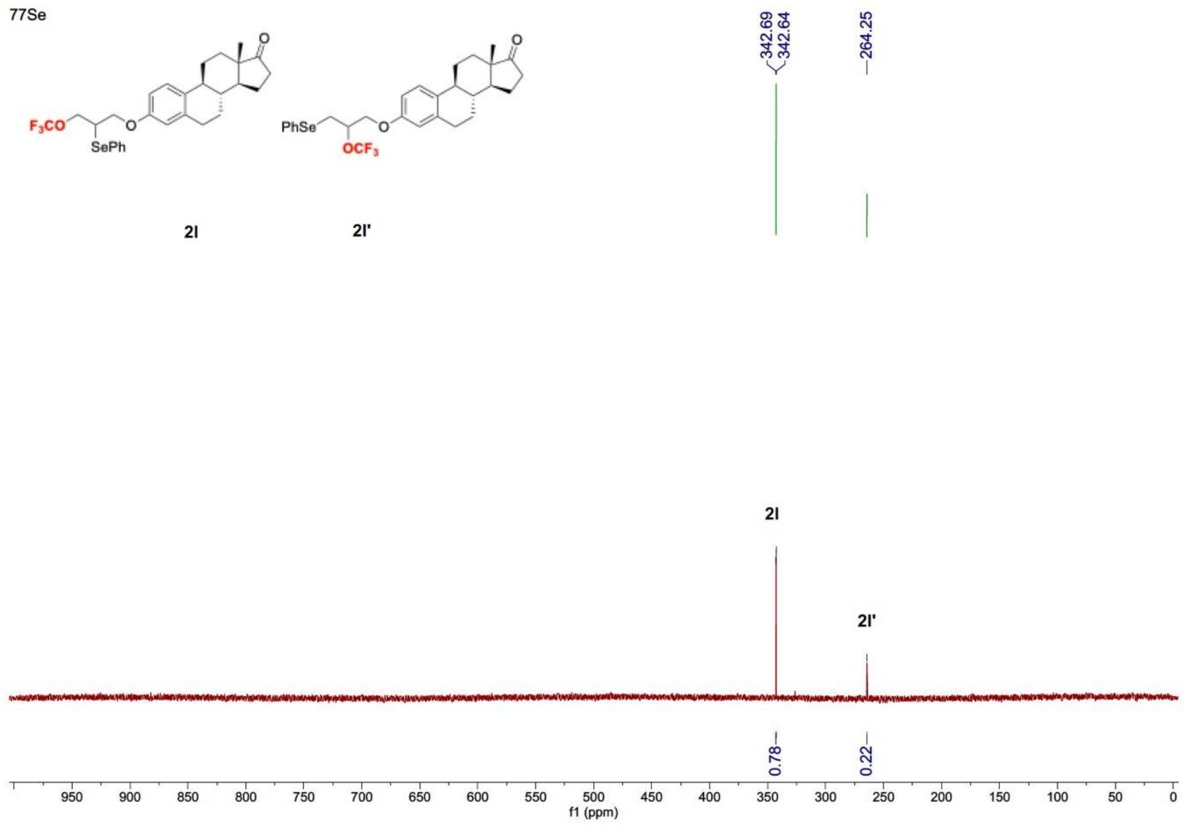
77Se
342.69
342.64
264.25
F3CO
SePh
PhSe
OCF3
2l
2l'
0.78
0.22
f1 (ppm)

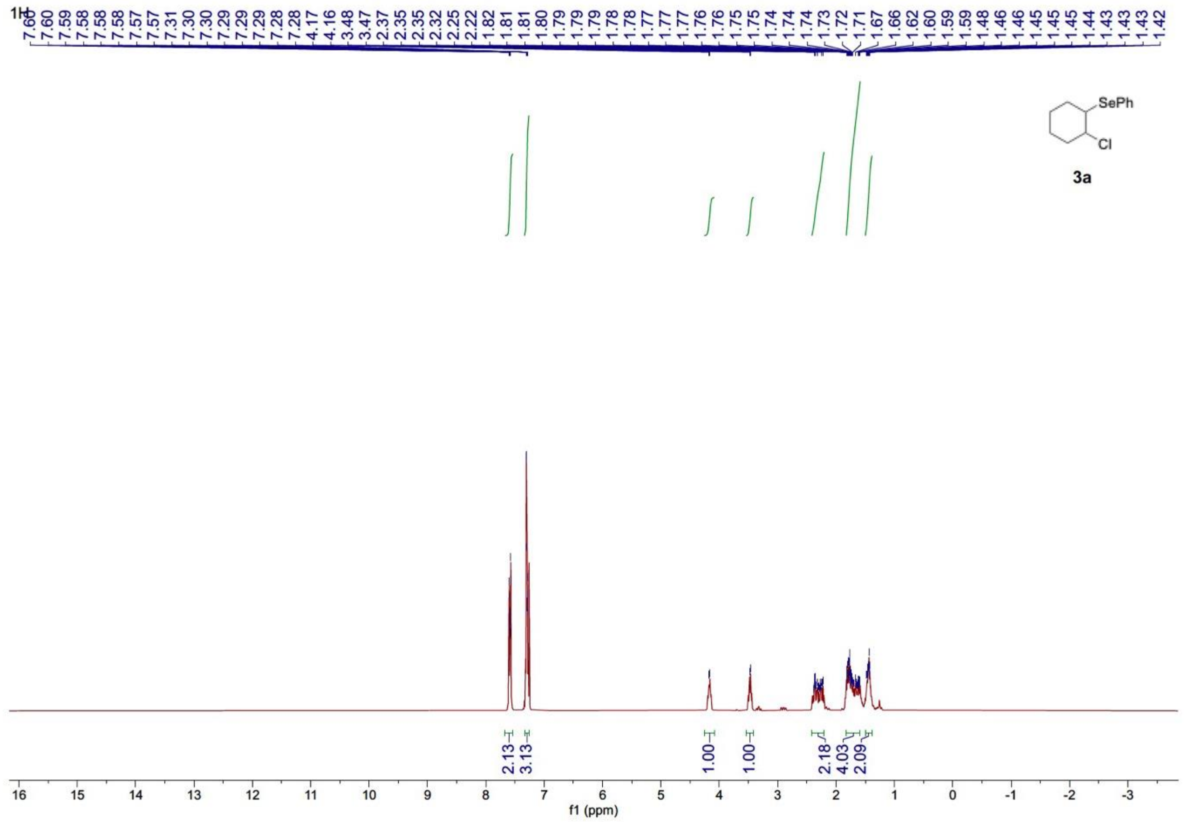
1H
SePh
Cl
3a
2.13
3.13
1.00
1.00
2.18
4.03
2.09
f1 (ppm)

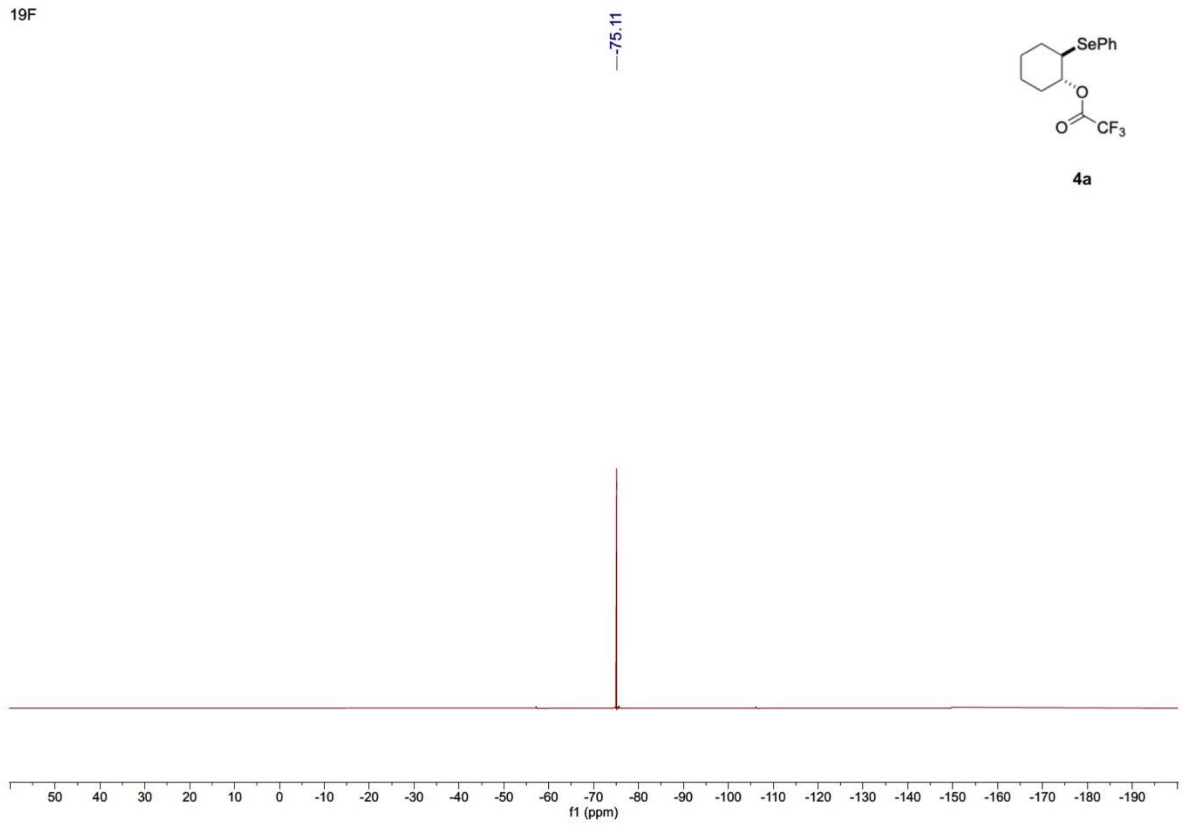
19F
-75.11
SePh
O
O
CF3
4a
f1 (ppm)

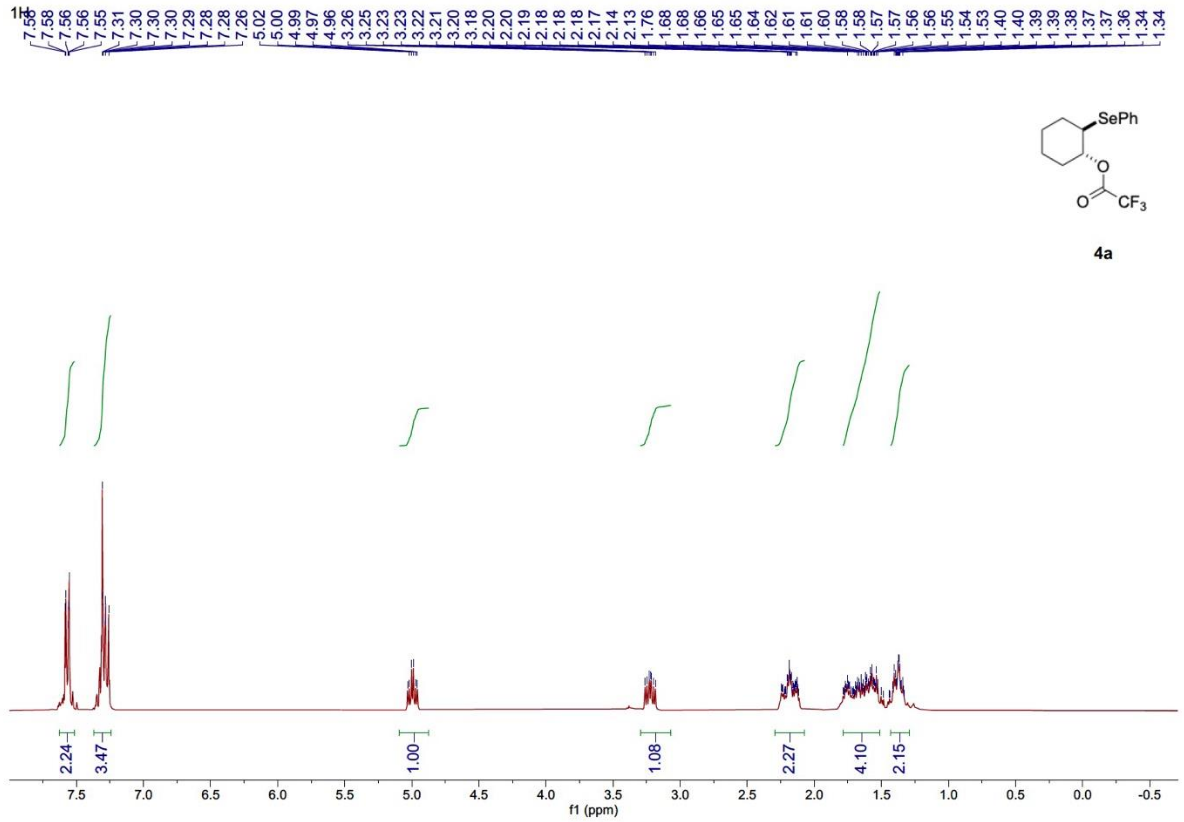
1H
SePh
O
O
CF$_3$
4a
2.24
3.47
1.00
1.08
2.27
4.10
2.15
f1 (ppm)

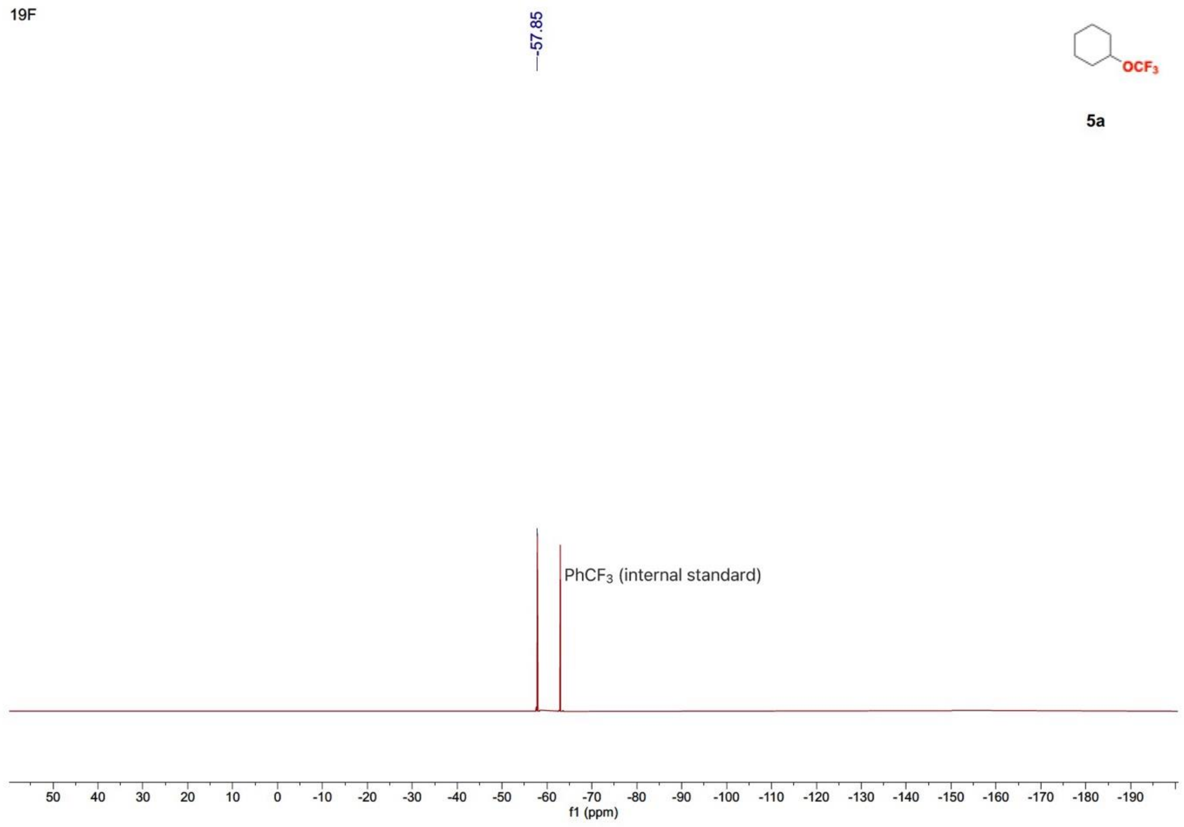
19F
-57.85
OCF$_3$
5a
PhCF$_3$ (internal standard)
f1 (ppm)

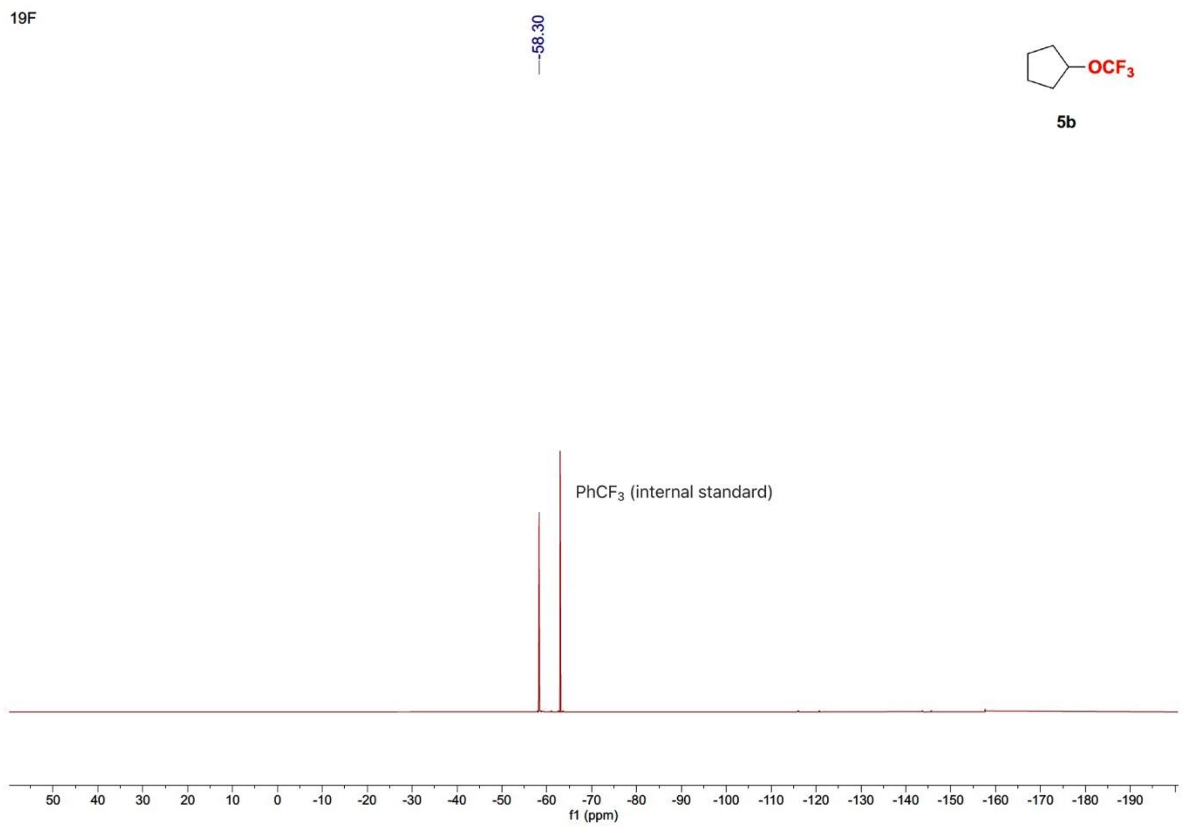
19F
-58.30
OCF3
5b
PhCF3 (internal standard)
50 40 30 20 10 0 -10 -20 -30 -40 -50 -60 -70 -80 -90 -100 -110 -120 -130 -140 -150 -160 -170 -180 -190
f1 (ppm)

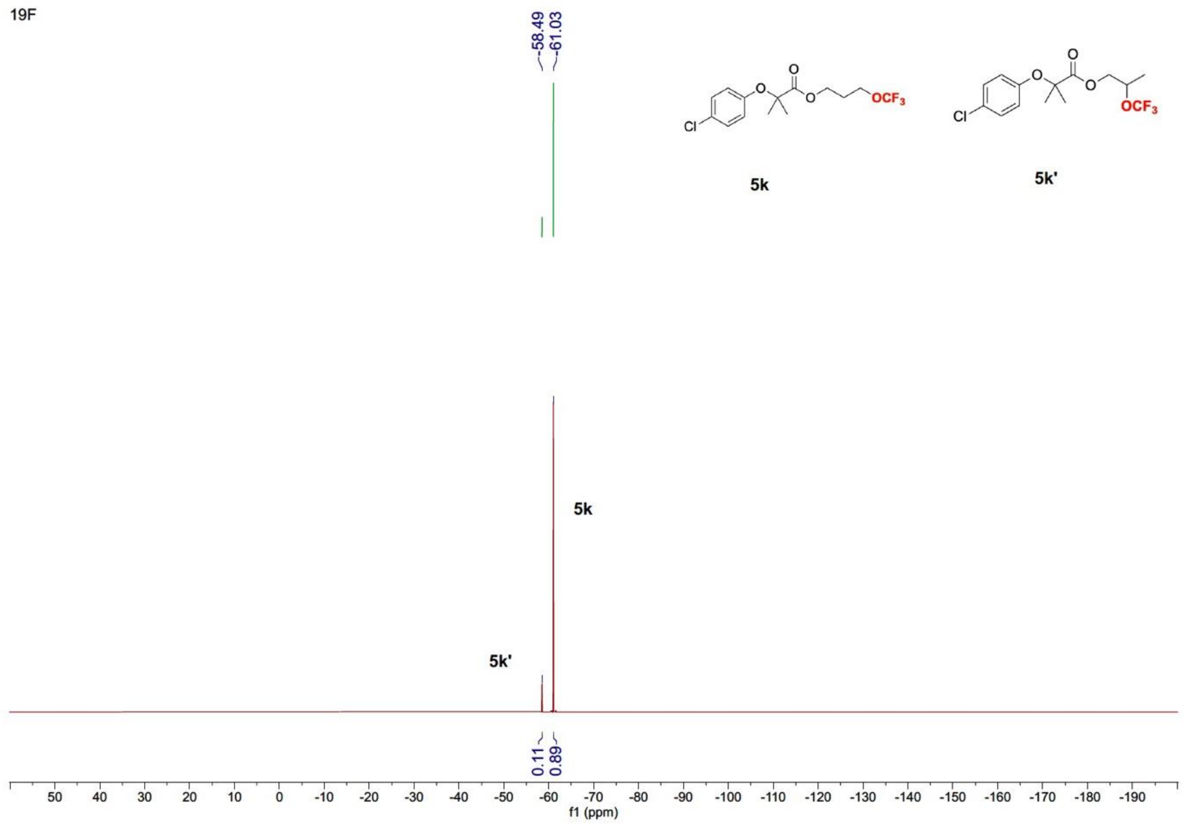
19F
-58.49
-61.03
Cl
O
O
O
OCF3
5k
5k'
0.11
0.89
50 40 30 20 10 0 -10 -20 -30 -40 -50 -60 -70 -80 -90 -100 -110 -120 -130 -140 -150 -160 -170 -180 -190
f1 (ppm)

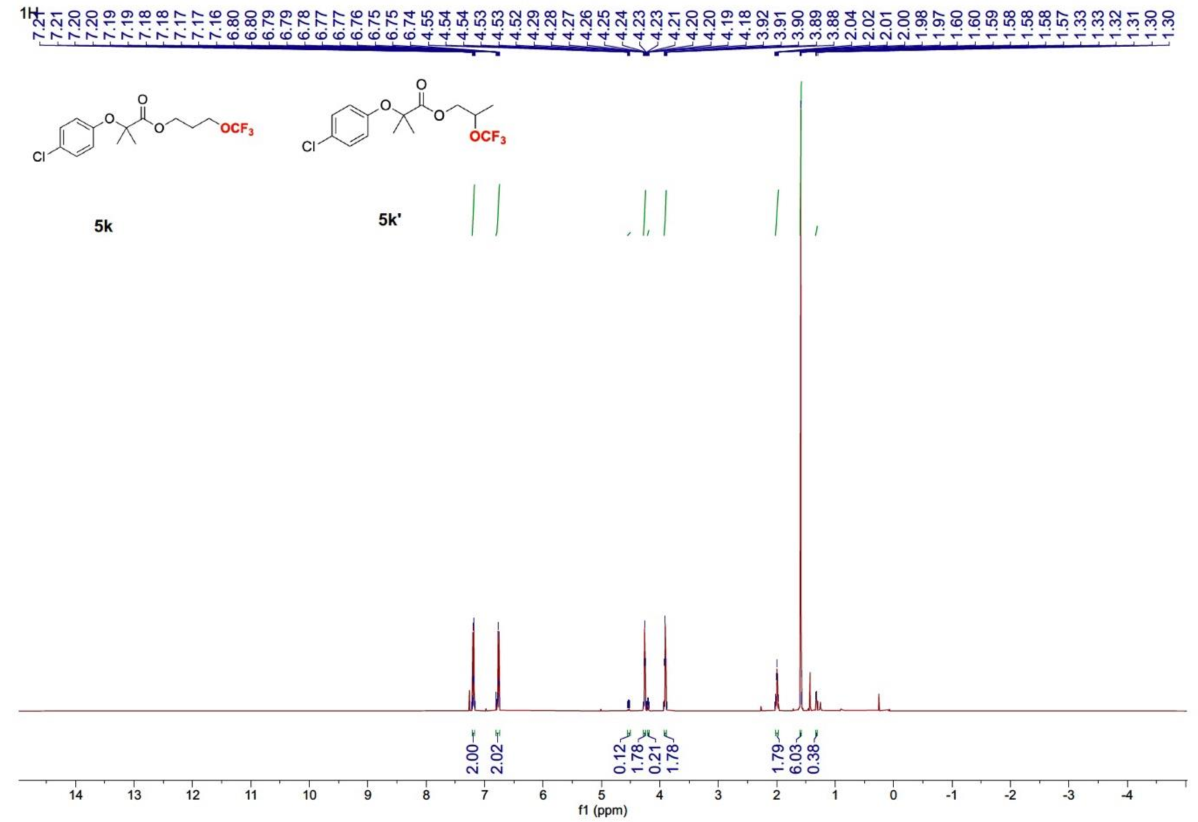

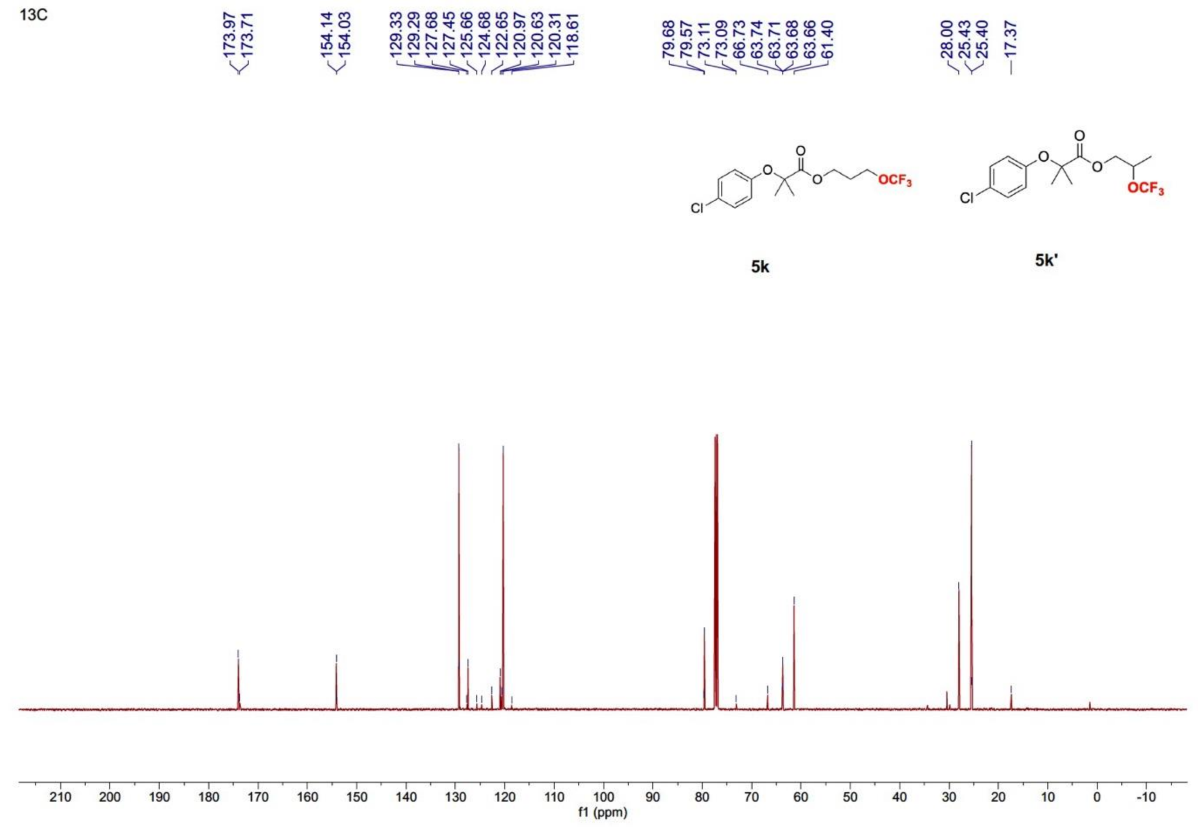

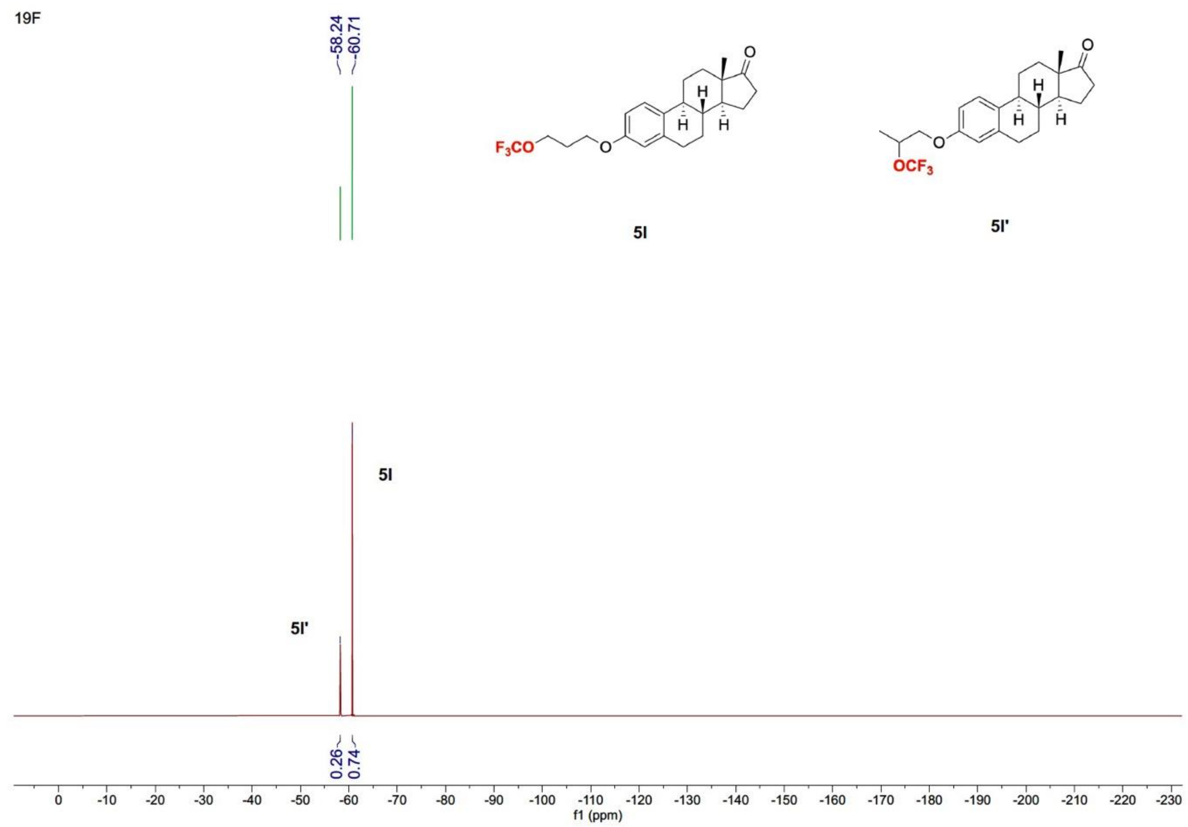
19F
-58.24
-60.71
F3CO
O
5l
OCF3
5l'
0.26
0.74
0 -10 -20 -30 -40 -50 -60 -70 -80 -90 -100 -110 -120 -130 -140 -150 -160 -170 -180 -190 -200 -210 -220 -230
f1 (ppm)

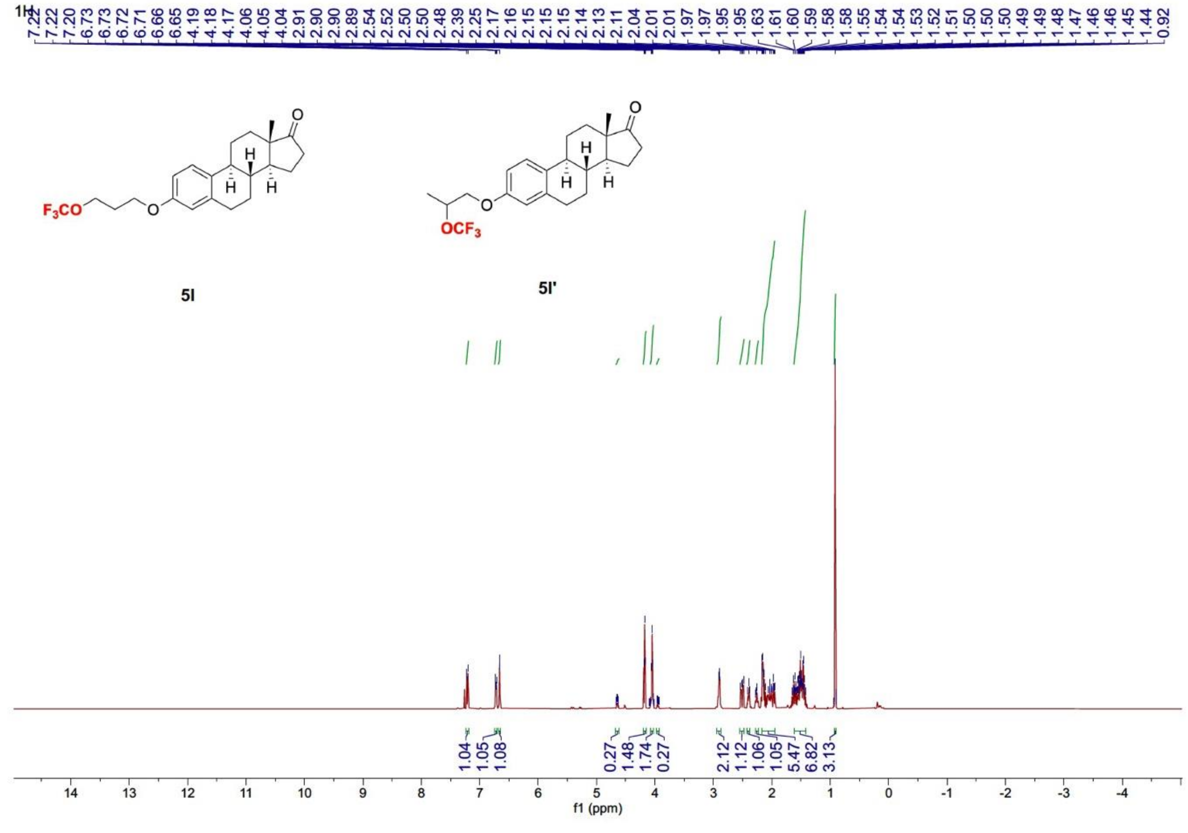
1H
7.22 7.22 7.20 6.73 6.73 6.72 6.71 6.66 6.65 4.19 4.17 4.06 4.05 4.04 2.91 2.90 2.90 2.89 2.54 2.52 2.50 2.50 2.48 2.39 2.25 2.17 2.16 2.15 2.15 2.15 2.14 2.13 2.11 2.04 2.01 2.01 1.97 1.95 1.95 1.63 1.61 1.60 1.59 1.58 1.58 1.55 1.54 1.54 1.53 1.52 1.51 1.50 1.50 1.50 1.49 1.48 1.47 1.46 1.45 1.44 0.92
F3CO
O
5l
OCF3
5l'
1.04 1.05 1.08 0.27 1.48 1.74 0.27 2.12 1.12 1.06 1.05 5.47 6.82 3.13
14 13 12 11 10 9 8 7 6 5 4 3 2 1 0 -1 -2 -3 -4
f1 (ppm)

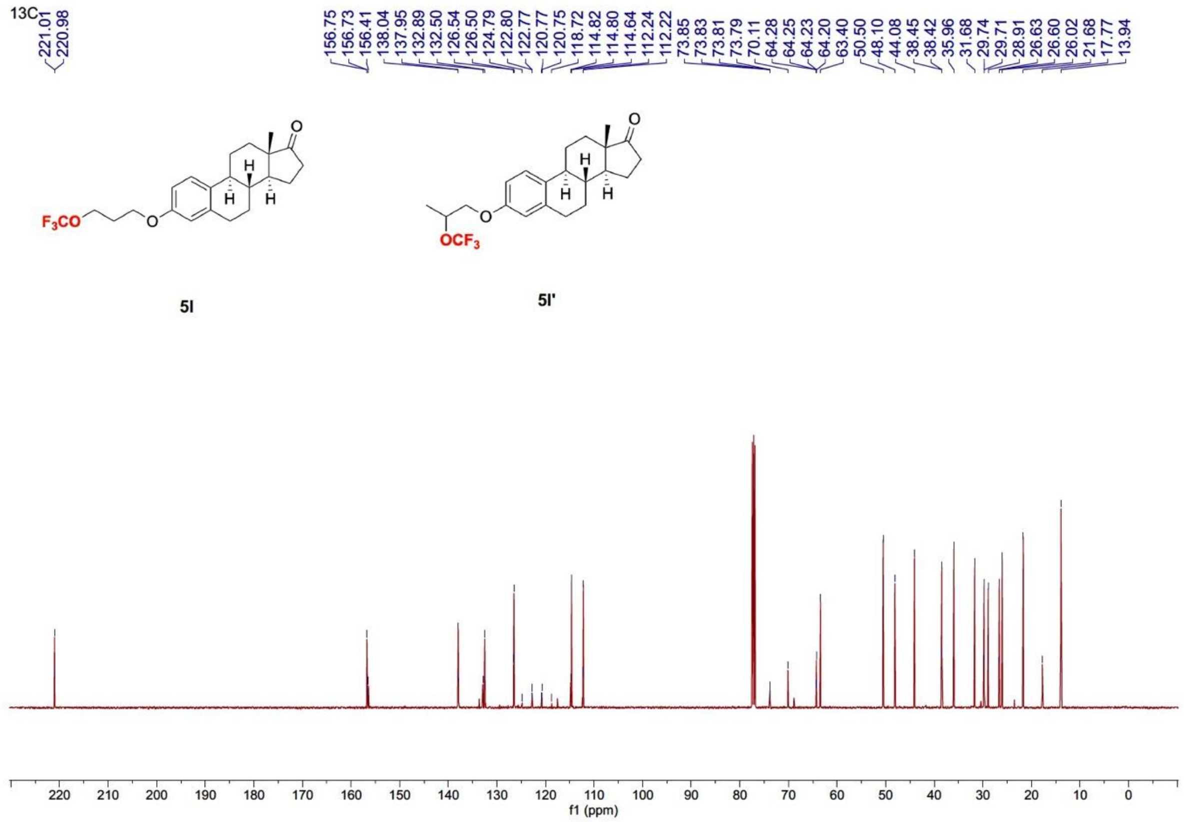
13C
221.01
220.98
156.75
156.73
156.41
138.04
137.95
132.89
132.50
126.54
126.50
124.79
122.80
122.77
120.77
120.75
118.72
114.82
114.80
114.64
112.24
112.22
73.85
73.83
73.81
73.79
70.11
64.28
64.25
64.23
64.20
63.40
50.50
48.10
44.08
38.45
38.42
35.96
31.68
29.74
29.71
28.91
26.63
26.60
26.02
21.68
17.77
13.94
F3CO
O
H
5l
OCF3
5l'
f1 (ppm)
